# Functionally impaired plasmacytoid dendritic cells and non-haematopoietic sources of type I interferon characterize human autoimmunity

**DOI:** 10.1038/s41467-020-19918-z

**Published:** 2020-12-01

**Authors:** Antonios Psarras, Adewonuola Alase, Agne Antanaviciute, Ian M. Carr, Md Yuzaiful Md Yusof, Miriam Wittmann, Paul Emery, George C. Tsokos, Edward M. Vital

**Affiliations:** 1 Leeds Institute of Rheumatic and Musculoskeletal Medicine, University of Leeds, Leeds, UK; 2 National Institute for Health Research (NIHR), Leeds Biomedical Research Centre, Leeds Teaching Hospitals NHS Trust, Leeds, UK; 3 Division of Rheumatology, Beth Israel Deaconess Medical Center, Harvard Medical School, Boston, MA USA; 4 Leeds Institute for Data Analytics, University of Leeds, Leeds, UK

**Keywords:** Autoimmunity, Plasmacytoid dendritic cells, SjÃ¶gren's disease, Systemic lupus erythematosus

## Abstract

Autoimmune connective tissue diseases arise in a stepwise fashion from asymptomatic preclinical autoimmunity. Type I interferons have a crucial role in the progression to established autoimmune diseases. The cellular source and regulation in disease initiation of these cytokines is not clear, but plasmacytoid dendritic cells have been thought to contribute to excessive type I interferon production. Here, we show that in preclinical autoimmunity and established systemic lupus erythematosus, plasmacytoid dendritic cells are not effector cells, have lost capacity for Toll-like-receptor-mediated cytokine production and do not induce T cell activation, independent of disease activity and the blood interferon signature. In addition, plasmacytoid dendritic cells have a transcriptional signature indicative of cellular stress and senescence accompanied by increased telomere erosion. In preclinical autoimmunity, we show a marked enrichment of an interferon signature in the skin without infiltrating immune cells, but with interferon-κ production by keratinocytes. In conclusion, non-hematopoietic cellular sources, rather than plasmacytoid dendritic cells, are responsible for interferon production prior to clinical autoimmunity.

## Abstract (web summary)

Type I interferon drives autoimmune pathology in SLE and has been assumed to come predominantly from plasmacytoid dendritic cells (pDCs). Here, the authors show that prior to the onset of SLE, pDCs lose multiple immunogenic functions and, instead, non-hematopoietic cells such as keratinocytes are a major source of type I interferons.

## Introduction

Systemic lupus erythematosus (SLE) and other autoimmune connective tissue diseases are a heterogeneous group of conditions. The pathogenesis of these diseases is incompletely understood. The most universal immune abnormality is the presence of autoantibodies targeting nuclear antigens. The dysregulation of the type I interferon (IFN) axis also has a fundamental role^1,2^. Many lupus susceptibility genes are related to the IFN pathway^3–7^. However, IFN activity is more variable; 60–80% of SLE patients exhibit increased expression of interferon-stimulated genes (ISGs) in peripheral blood^8–11^.

Autoimmune connective tissue diseases are now recognized to arise in a stepwise fashion from asymptomatic preclinical autoimmunity. Autoantibodies precede symptoms by years and are far more common than clinical autoimmune disease^12–16^. Hence, autoantibody-positive individuals constitute an At-Risk population of whom a minority will develop clinical autoimmunity. A key determinant of progression from At-Risk to established clinical autoimmune disease is the level of IFN activity^12^. In established SLE, IFN activity is particularly associated with cutaneous involvement. It also predicts future flares and severity^8,17–19^. In the present study, we, therefore, asked how IFN production is controlled and regulated at both the preclinical and established autoimmune disease stages.

Most hematopoietic and non-hematopoietic cells are capable of producing type I IFNs (IFN-α, -β, -κ, -ω, -ε) as the first line of defense against viral infections. Nevertheless, much previous research on IFN production has focused on plasmacytoid dendritic cells (pDCs). pDCs from otherwise healthy individuals produce particularly large amounts of type I IFNs upon recognition of viral antigens via endosomal toll-like receptor 7 (TLR7) and TLR9^20,21^. Engagement of TLRs within endosomal compartments with these ligands leads to activation of IRF7 and NF-κB pathways and eventually production of IFN-α and other pro-inflammatory cytokines (TNF, IL-6)^22^. Apart from this secretory function, pDCs exhibit antigen-presentation properties inducing both immunogenic and tolerogenic T-cell responses^23–28^. Conversely, the IFN-α-producing capacity of pDCs has been shown to be impaired in melanoma and ovarian cancer; tumor-infiltrating pDCs do not produce IFN-α but their presence actually promotes tumor growth^29–31^. Additionally, the hepatitis B virus can interfere with the TLR9 pathway by blocking MyD88-IRAK4 signaling and Sendai virus by targeting IRF7, while lymphocytic choriomeningitis virus (LCMV) compromises the capacity of pDCs to secrete type I IFNs^32–34^.

In the context of autoimmunity, endogenous nucleic acids forming immune complexes with autoantibodies have been proposed as a stimulus for pDC activation^35–38^. While it is therefore natural to assume that pDCs are dominant producers of IFN-α in SLE, in fact, the existing literature is complex and contradictory. Previous studies have reported both higher and lower numbers of pDCs in blood^39,40^. Unsorted PBMCs from SLE patients have been shown to produce lower levels of IFN-α in response to TLR9 stimulation, while other studies reported enhanced TLR7-mediated IFN-α production by pDCs of SLE patients^41,42^. It is not clear whether any alteration in the pDC phenotype is the result of chronic inflammation or therapy, nor what underlying mechanism determines their function in the early stages of human autoimmunity.

In order to resolve these contradictions, here we assess pDC phenotype, function, and transcriptomic profile in At-Risk individuals who are therapy-naive and do not have tissue inflammation as well as in established SLE and primary Sjögren’s Syndrome (pSS). We show that pDC numbers, TLR-mediated cytokine production, and T-cell-activating capacity are decreased prior to the onset of SLE. This impairment is unrelated to the type I IFN signature in the blood. Instead, pDCs have a transcriptomic profile indicative of cellular stress and senescence. By contrast, we show that non-hematopoietic tissue-resident cells in the skin are not passive targets. Rather, these cells actively contribute to aberrant type I IFN production in both preclinical and established autoimmunity. These findings provide unique insights into the source and regulation of type I IFNs in human autoimmune disease.

## Results

### pDC numbers are reduced in preclinical autoimmunity and SLE

**Fig. 1:**
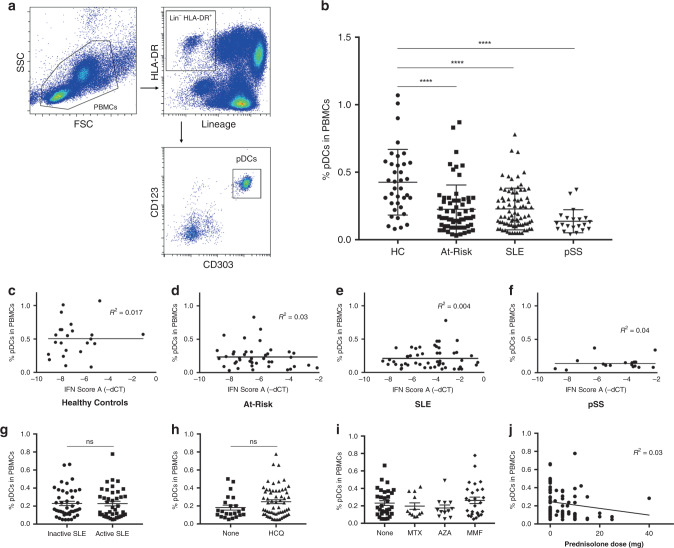
Decrease in circulating pDCs in autoimmunity is independent of disease activity and therapy. **a** Gating strategy to identify the pDC population within PBMCs: pDCs are characterized by the lack of expression of lineage markers (CD3, CD19, CD56, CD14, CD11c), intermediate to high expression of HLA-DR, high expression of CD123 (IL-3R) and CD303 (BDCA-2). **b** Average percentage of pDCs in PBMCs of age- and sex-matched healthy controls (HC; *n* = 37), At-Risk individuals (At-Risk; *n* = 64), patients with systemic lupus erythematosus (SLE; *n* = 81) and primary Sjögren’s Syndrome (pSS; *n* = 21). **c**–**f** Association between the percentage of pDCs in PBMCs and type I IFN activity in the blood (IFN score A) in HC, At-Risk, SLE, and pSS. **g** Percentage of pDCs in PBMCs in SLE patients with inactive and active disease. **h** Percentage of pDCs in PBMCs in SLE patients treated with or without hydroxychloroquine (HCQ). **i** Percentage of pDCs in PBMCs in SLE patients treated with other immunosuppressants (MTX = methotrexate, AZA = azathioprine, MMF = mycophenolate mofetil). **j** Association between the percentage of pDCs in PBMCs and the dose of prednisolone in patients with SLE. Data are represented as mean ± SEM. ns = not significant; ****P < 0.0001. Two-way ANOVA (**b**), nonlinear regression (**c**–**f** and **j**), unpaired two-tailed *t*-test (**g**–**i**).

Peripheral blood pDCs were enumerated and immunophenotyped from freshly isolated peripheral blood mononuclear cells (PBMCs) using flow cytometry. We analyzed samples from At-Risk individuals (defined by ANA+, ≤1 clinical criterion for SLE, symptom duration <12 months and treatment-naive; *n* = 64), patients with SLE (*n* = 81) and pSS (*n* = 21) as well as age- and sex-matched healthy controls (*n* = 37). Clinical characteristics and treatment of SLE patients can be seen in Supplementary Table 1. pDCs were gated as CD3^−^CD19^−^CD14^−^CD56^−^CD11c^−^HLA-DR^+^CD123^+^CD303^+^ cells (Fig. 1a). The average percentage of pDCs in PBMCs was significantly decreased in patients with SLE and pSS in comparison with healthy controls, a finding which was also observed in treatment-naive At-Risk individuals (Fig. 1b). The percentage and absolute numbers of pDCs in the peripheral blood of all samples analyzed were closely correlated (Supplementary Fig. 1a).

To determine whether the reduction of circulating pDCs was associated with blood ISG expression and other clinical features, we evaluated the expression of a previously validated IFN score (IFN score A) in PBMCs using TaqMan in all sample groups described above^8^. Increased type I IFN activity, as assessed by IFN score A, was observed in patients with SLE and pSS as well as At-Risk individuals compared to healthy controls, but the reduction of peripheral blood pDCs described above was not associated with the higher expression of IFN score A (Fig. 1c–f). Although IFN score A was associated with an increased number of extractable nuclear antigen (ENA) antibodies, no association was found between the percentage of pDCs and either IFN score A or ENA antibodies in any of the sample groups (Supplementary Fig. 1b, c). Additionally, in SLE patients the reduction of circulating pDCs was independent of disease activity (Fig. 1g), treatment with hydroxychloroquine (Fig. 1h), other immunosuppressants (Fig. 1i), or prednisolone (Fig. 1j). Apart from that, the reduction of circulating pDCs in At-Risk individuals, patients with SLE and pSS was not associated with the total lymphocyte count, which is commonly low in SLE patients (Supplementary Fig. 1d–f).

pDCs from healthy controls, At-Risk individuals, and SLE patients were analyzed for the surface expression of multiple molecules known to be important in regulating their immune functions (Supplementary Fig. 2a–f). pDCs in SLE patients showed no statistically significant difference in the expression of HLA-DR or BDCA-2 (CD303). On the other hand, CD123 (IL-3R) and ILT2 (CD85j) were found to be upregulated on pDCs of SLE patients compared to healthy controls (*P* < 0.001). Interestingly, CD317 (BST2; tetherin), a molecule is known to be induced by type I IFNs also presented higher expression on pDCs of SLE patients (*P* < 0.05); however, its ligand ILT7 (CD85g) appeared to be downregulated on pDCs of SLE patients (*P* < 0.05).

### TLR-stimulated pDCs produce less cytokines in autoimmunity

**Fig. 2:**
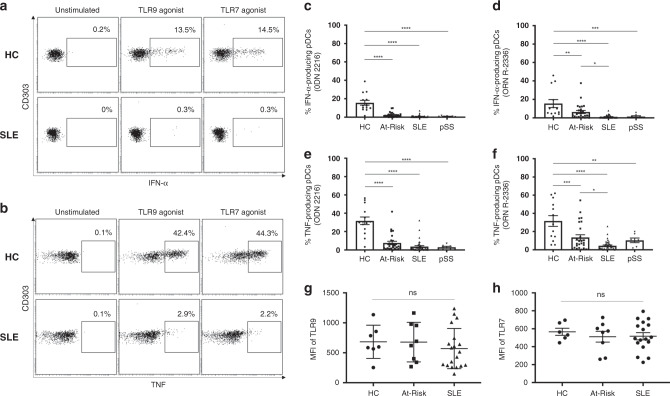
TLR-stimulated pDCs produce less IFN-α and TNF in autoimmunity. **a**, **b** Freshly isolated PBMCs were cultured in the absence or presence of TLR9 (ODN 2216) or TLR7 (ORN R-2336) agonists for 6 h, then IFN-α and TNF production by pDCs was measured using intracellular staining. Results shown are representative of healthy control (HC) and a patient with SLE. The average percentage of IFN-α produced by TLR9-stimulated (**c**) and TLR7-stimulated (**d**) pDCs in HC (*n* = 14), At-Risk (*n* = 26), SLE (*n* = 40), and pSS (*n* = 7) patients. The average percentage of TNF produced by TLR9-stimulated (**e**) and TLR7-stimulated (**f**) pDCs in HC (*n* = 14), At-Risk (*n* = 26), SLE (*n* = 40), and pSS (*n* = 7) patients. **g**, **h** Intracellular expression of TLR9 and TLR7 was measured using flow cytometry in HC (*n* = 7), At-Risk (*n* = 8), and SLE (*n* = 19) patients. Data are represented as mean ± SEM. *P < 0.05; **P < 0.01; ***P < 0.001; ****P < 0.0001. Two-way ANOVA (**c**–**h**).

The production of IFN-α and other pro-inflammatory cytokines (e.g. TNF) in response to TLR-mediated stimulation is the hallmark of normal pDC function. To evaluate the capacity of cytokine production by pDCs, we stimulated freshly isolated PBMCs from At-Risk individuals (*n* = 26), patients with established SLE (*n* = 40) and pSS (*n* = 7) alongside healthy controls (*n* = 14) for 6 h with TLR9 (ODN 2216) or TLR7 (ORN R-2336) agonists. We measured both IFN-α and TNF produced by CD3^−^CD19^−^CD14^−^CD56^−^CD11c^−^HLA-DR^+^CD123^+^CD303^+^ pDCs using intracellular staining. No IFN-α and/or TNF production by pDCs was detected in any of the samples without external stimulation. While pDCs from healthy controls produced large amounts of IFN-α in response to TLR9 or TLR7 agonists, pDCs from SLE patients showed little or no cytokine production (Fig. 2a). Furthermore, TLR9- and TLR7-mediated IFN-α production was diminished in pDCs from patients with pSS similarly to SLE (Fig. 2c, d). Although TLR9-mediated IFN-α production was similarly reduced in At-Risk individuals, their pDCs seemed to partially maintain some TLR7-mediated IFN-α production (Fig. 2d). TLR9- and TLR7-mediated TNF production was also significantly decreased in pDCs from patients with SLE and pSS compared to healthy controls (Fig. 2b), whilst pDCs from At-Risk individuals showed the same trend as for IFN-α production, partially maintaining some TLR7-mediated TNF production (Fig. 2e, f).

We next evaluated whether there was any association between IFN-α production by pDCs and overall blood type I IFN activity as measured by IFN score A. We found no association between the levels of TLR-mediated IFN-α production and the level of IFN score A in patients with SLE and pSS as well as At-Risk individuals (Supplementary Fig. 3a–d). To confirm that these findings were not due to differences in TLR expression, we measured the expression levels of both TLR9 and TLR7 using flow cytometry. pDCs from At-Risk individuals and SLE patients showed similar expression levels of both receptors compared to those of healthy controls (Fig. 2g, h). There was no correlation between IFN-α production and the intracellular expression of either TLR9 or TLR7 (Supplementary Fig. 3e, f).

Interestingly, while culturing PBMCs, we observed that a population within monocytes was characterized by no expression of HLA-DR, which is expressed by all pDCs, but a positive expression of CD303 (BDCA-2), which was previously thought to be a pDC-specific marker and used in immunohistochemistry. These cells showed no response to TLR stimulation, as neither IFN-α nor TNF production was detected (Supplementary Fig. 3g, h).

### IL-3 triggers TLR-independent production of IL-6 by pDCs

**Fig. 3:**
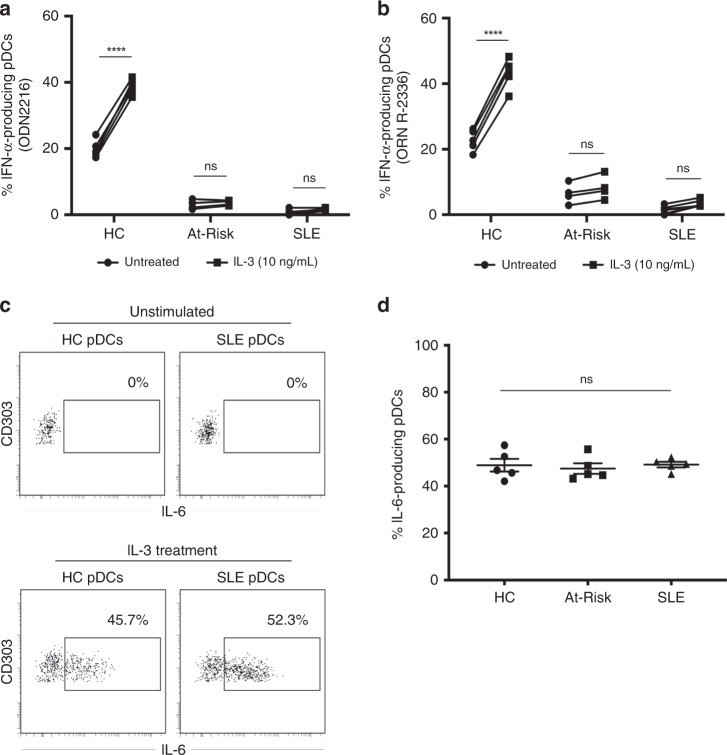
IL-3 triggers TLR-independent production of IL-6 by pDCs. PBMCs from healthy controls (HC; *n* = 6), At-Risk individuals (At-Risk; *n* = 4), and SLE patients (*n* = 7) were cultured for 18 h in the absence or presence of IL-3 (10 ng/mL). The cells were then stimulated by TLR9 (ODN 2216) or TLR7 (ORN R-2336) agonists for six additional hours. The production of cytokines was measured by intracellular staining. **a** IL-3 significantly enhanced TLR9-mediated IFN-α production by pDCs of healthy controls (*P* < 0.0001); this effect was not seen in pDCs of At-Risk (*P* = 0.94) and SLE (*P* = 0.53) patients. **b** IL-3 significantly enhanced TLR7-mediated IFN-α production by pDCs of healthy controls (*P* < 0.0001); this effect was not that prominent in pDCs of At-Risk (*P* = 0.71) and SLE (*P* = 0.43) patients. **c** Treatment with IL-3 (10 ng/mL) induced the production of IL-6 by pDCs of both healthy controls and SLE patients without exogenous TLR stimulation. **d** No difference was found in IL-6 production by the pDCs of healthy controls, At-Risk individuals, and SLE patients after stimulation with IL-3. The production of IL-6 was detected by intracellular staining. Data are represented as mean ± SEM. ***P < 0.001; ns = not significant. Two-way ANOVA (**a**–**d**).

IL-3 is known to maintain pDC survival in vitro and to enhance IFN-α production upon TLR-mediated stimulation^43,44^. We confirmed that pre-treatment for 24 h with IL-3 amplified IFN-α production by both TLR9- and TLR7-stimulated pDCs from healthy controls (*n* = 6). However, a statistically significant enhancement in IFN-α production was not seen in pDCs of At-Risk individuals (*n* = 4) and SLE patients (*n* = 7) (Fig. 3a, b). Furthermore, we discovered an additional function of pDCs; IL-3 triggered the spontaneous production of IL-6 by pDCs without any exogenous TLR-mediated stimulation. In contrast to the defective TLR-mediated IFN-α and TNF production in pDCs from SLE patients and At-Risk individuals we described above, this TLR-independent IL-6 production upon IL-3 stimulation was not impaired in pDCs of any of the groups tested (Fig. 3c, d).

### T-cell activation by pDCs is impaired in autoimmunity

**Fig. 4:**
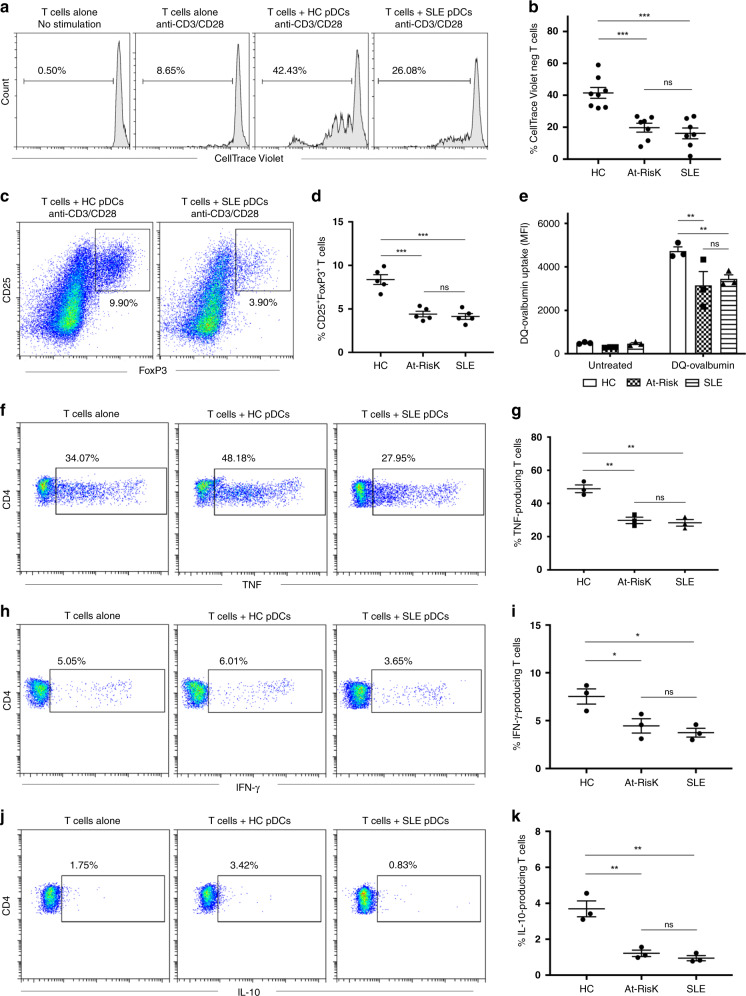
pDCs display impaired T-cell activation in autoimmunity. **a** Allogeneic naive CD4^+^ T cells were labeled with CellTrace Violet and cultured alone or with pDCs purified from healthy controls (HC) or patients with active SLE for 5 days in the presence of anti-CD3/CD38 beads at ratio 2:1 to avoid excessive T-cell activation and expansion. T-cell proliferation was analyzed by flow cytometry based on CellTrace Violet dilution. One representative experiment is shown out of four independent experiments. **b** Average percentage of proliferated CD4^+^ T cells co-cultured with pDCs from healthy controls (*n* = 8), At-Risk individuals (*n* = 7), and SLE patients (*n* = 7). **c** Induction of CD4^+^CD25^high^FoxP3^+^ T cells from naive CD4^+^ T cells co-cultured for 5 days with pDCs from healthy controls or SLE patients in the presence of anti-CD3/CD28 beads at ratio 2:1. One representative experiment is shown out of three independent experiments. **d** Percentage of CD4^+^CD25^high^FoxP3^+^ T cells derived from the co-culture with pDCs from healthy controls (*n* = 5), At-Risk individuals (*n* = 5), and SLE patients (*n* = 5). **f**–**k** Allogeneic naive CD4^+^ T cells were cultured alone or with pDCs from healthy controls or SLE patients for 5 days in the presence of anti-CD3/CD38 beads at ratio 2:1. On the fifth day, the cells were stimulated with PMA/Ionomycin, and the production of TNF (**f**), IFN-γ (**h**), and IL-10 (**j**) by CD4^+^ T cells was measured by intracellular staining. One representative experiment is shown out of three independent experiments. The average percentage of TNF (**g**), IFN-γ (**i**), and IL-10 (**k**) produced CD4^+^ T cells co-cultured with pDCs from healthy controls, At-Risk individuals, and SLE patients. Data are represented as mean ± SEM. *P < 0.05; **P < 0.001; ***P < 0.0001; ns = not significant. Two-way ANOVA (**b**, **d**, **e**, **g**, **i**, **k**).

Although pDCs possess antigen-presentation properties and can trigger T-cell responses, little is known about the capacity of pDCs in SLE to induce T-cell proliferation and activation. We co-cultured freshly isolated pDCs from patients with active SLE, At-Risk individuals, and healthy controls with CellTrace Violet-labeled allogeneic naive CD4^+^ T cells in the presence of low ratio anti-CD3/CD28 beads for 5 days (Fig. 4a). Although pDCs from all groups induced T-cell proliferation, pDCs from SLE patients and At-Risk individuals were substantially less efficient in inducing T-cell proliferation (Fig. 4b). pDCs are also known to trigger the induction of FoxP3^+^ T cells (a marker of T-cell activation). Following the same protocol as above, we found that fewer CD25^high^FoxP3^+^ cells were generated from naive CD4^+^ T cells after 5 days of co-culturing with pDCs from SLE patients and At-Risk individuals in comparison with pDCs from healthy controls (Fig. 4c, d).

For additional confirmation that pDCs from At-Risk individuals and SLE patients were not switching to an antigen-presenting phenotype, we tested antigen uptake from the cells using DQ Ovalbumin. First, pDCs were purified from freshly isolated PBMCs as described above and were then cultured alone or with 10 μg/mL DQ Ovalbumin. After 18 h, pDCs were harvested and DQ Ovalbumin processing was analyzed by flow cytometry based on the level of mean fluorescence intensity. Almost all pDCs from healthy controls were able to internalize and process DQ Ovalbumin as compared to the negative control. However, pDCs from both At-Risk individuals and SLE patients had significantly reduced antigen uptake, indicating impaired antigen processing and presenting properties (Fig. 4e).

To investigate the ability of pDCs to trigger cytokine production by T cells, we first co-cultured pDCs from patients with active SLE, At-Risk individuals, and healthy controls with allogeneic naive CD4^+^ T cells in the presence of low ratio anti-CD3/CD28 beads for 5 days before adding PMA/Ionomycin in the last 5 h of the culture. In comparison with T cells alone, pDCs from healthy controls enhanced the production of TNF (34.07% vs. 48.18%), IFN-γ (5.05% vs. 6.01%), and IL-10 (1.75% vs. 3.42%) from the co-cultured T cells (Fig. 4f–k). However, pDCs from SLE patients suppressed the production of all cytokines measured; TNF (34.07% vs. 27.95%), IFN-γ (5.05% vs. 3.65%), and IL-10 (1.75% vs. 0.83%). Similar results were observed for pDCs from At-Risk Individuals. In summary, pDCs from SLE patients and At-Risk individuals exhibit a decreased capacity for triggering T-cell proliferation and activation, whilst they actively inhibited cytokine production by T cells.

### pDCs cluster according to ISG expression in RNA-sequencing

To investigate disease-associated transcriptional changes in pDCs, we purified pDCs from healthy controls (*n* = 8), At-Risk individuals (*n* = 4) and SLE patients (*n* = 13) by negative selection then sorted the cells to achieve purity >99% based on CD304 (BDCA-4) expression. We sequenced the RNA extracted from sorted pDCs using Smart-seq2 for sensitive full-length transcriptomic profiling.

**Fig. 5:**
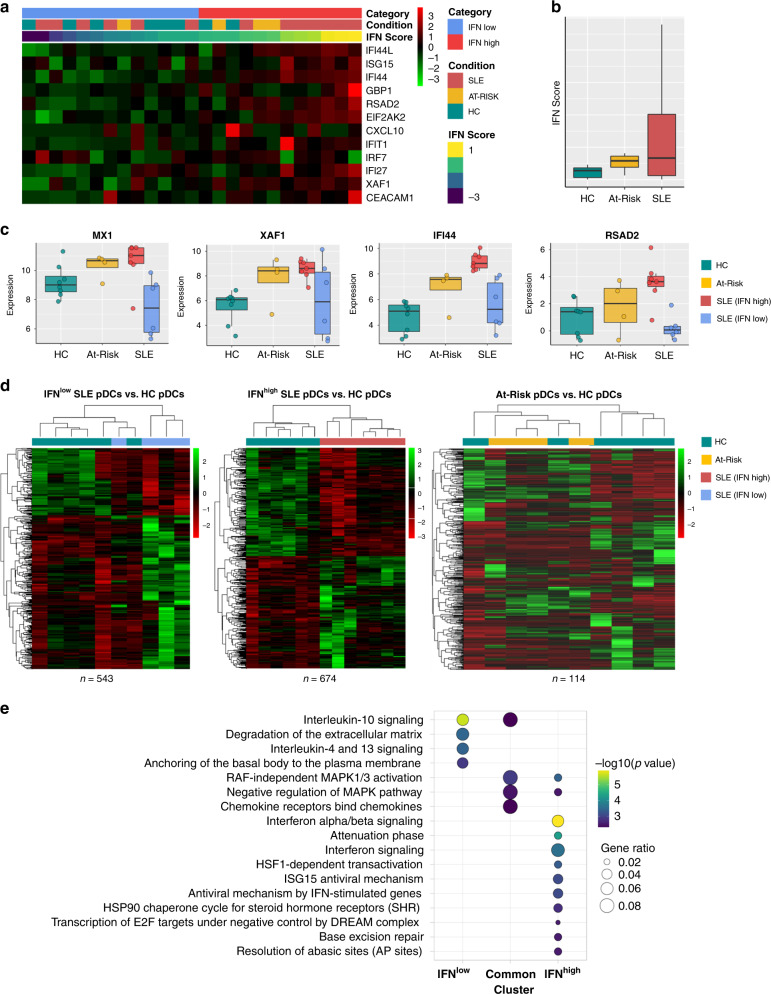
pDCs from IFN^low^ and IFN^high^ SLE patients have distinct transcriptomic profiles. **a** Sorted pDCs from HC (*n* = 7), At-Risk (*n* = 4), and SLE (*n* = 13) were classified according to the expression level of the IFN score described. **b** Average expression level of IFN score measured in samples described in **a**. **c** Expression level of representative ISGs in sorted pDCs from sample groups described in **a**. **d** Differentially expressed transcripts in IFN^low^ SLE pDCs (*n* = 543), IFN^high^ SLE pDCs (*n* = 674), and At-Risk pDCs (*n* = 114) compared to HC pDCs. **e** Summary of the Reactome Pathway Enrichment in differentially expressed genes of the pDCs from IFN^low^ and IFN^high^ subgroups compared to HC pDCs.

A major source of variability amongst the samples was due to the expression of ISGs. In the RNA-sequencing analysis, pDCs clustered according to ISG expression rather than according to the clinical diagnosis. In order to control for this variability, we therefore first scored each sample based on the expression profile of a core set of ISGs (IFN score). The expression level of IFN score in pDCs from healthy controls (95% CI) was used to assign each sample to IFN^low^ or IFN^high^ subgroups (Fig. 5a). As expected, pDCs from SLE patients were characterized by a range of IFN scores, but overall exhibited a higher IFN score than pDCs from healthy controls and At-Risk individuals (Fig. 5b). pDCs from most At-Risk individuals presented a higher IFN score compared to pDCs from healthy controls and three of the four were assigned to the IFN^high^ subgroup. pDC RNA-sequencing data was consistent with the blood IFN score used for categorization. Common ISGs (*MX1*, *XAF1*, *IFI44*, *RSAD2*) were found to be upregulated in the majority of pDCs in IFN^high^ SLE patients and At-Risk individuals, whilst pDCs of IFN^low^ SLE patients showed similar expression levels to those of healthy controls (Fig. 5c).

### pDCs of IFN subgroups have distinct transcriptional profiles

The analysis of IFN^low^ SLE patients revealed 543 transcripts that were significantly (FDR < 5%) differentially expressed (Fig. 5d). These were particularly enriched for IL-4 and IL-13 signaling, IL-10 signaling, cell migration, and pathogen interaction pathways, among others (Supplementary Fig. 4a). Amongst the upregulated genes were those corresponding to chemokines, for instance, *CXCL3*, *CXCL2,* and *CXCL16* (Supplementary Fig. 5a). A detailed table of the top differentially expressed genes in pDCs of IFN^low^ SLE patients can be found in Supplementary Table 2.

In IFN^high^ SLE patients, we found 674 transcripts that were significantly (FDR < 5%) differentially expressed (Fig. 5d). Unsurprisingly, these genes were found to be heavily enriched for IFN-response pathways (Supplementary Fig. 4b), but also pathways related to DNA repair and MAPK signaling. Several phosphatases are known to dephosphorylate MAP kinases (*DUSP1*, *DUSP2*, *DUSP5,* and *DUSP8*), transcriptional repressors associated with cell differentiation (*HESX1*, *ETV3*), and NF-κB inhibitors (*NFKBIA*, *NFKBID*) were found to be upregulated in IFN^high^ SLE patients (Supplementary Fig. 5b–d). A detailed table of the top differentially expressed genes in pDCs of IFN^high^ SLE patients can be found in Supplementary Table 3.

In At-Risk individuals, we found 114 transcripts that were significantly (FDR < 5%) differentially expressed compared to healthy controls (Fig. 5d). The main pathways enriched were associated with regulation of innate immune responses, response to type I IFN and viruses as well as response to reactive oxygen species and hydrogen peroxide (Supplementary Fig. 7a). A detailed table of the top differentially expressed genes in pDCs of At-Risk individuals can be found in Supplementary Table 5. Between At-Risk and IFN^low^ SLE, there were 285 differentially expressed transcripts (Supplementary Fig. 6a). A detailed table of the top differentially expressed of these is shown in Supplementary Table 6. Comparing At-Risk and IFN^high^ SLE, there were 356 differentially expressed transcripts (Supplementary Fig. 7b). Due to the fact that At-Risk pDCs were clustered into the IFN^high^ subgroup, IFN-response pathways were similarly enriched. A detailed table of the top differentially expressed genes in pDCs of At-Risk individuals and IFN^high^ SLE patients can be found in Supplementary Table 7. Amongst the commonly enriched pathways of At-Risk and SLE pDCs compared to healthy controls were regulation of MAPK activity, neutrophil regulation, and chemotaxis (Supplementary Fig. 7b).

Given that pDCs clustered according to IFN status rather than clinical diagnosis, we analyzed the differentially expressed transcripts in the IFN^high^ and IFN^low^ subgroups (including both At-Risk individuals and SLE patients) in comparison to the healthy controls. We also analyzed those pathways that were commonly involved in both subgroups (Fig. 5e). The pathways are common to both subgroups related to IL-10 signaling, RAF-independent MAPK 1/3 activation, negative regulation of MAPK pathways, and chemokine receptor binding (Supplementary Fig. 4c).

We also evaluated the expression levels of multiple TLRs in the pDCs of all samples (Supplementary Fig. 9). As expected, pDCs from healthy controls, At-Risk individuals, and SLE patients strongly expressed TLR9 and TLR7; however, no differences were found among the different groups regardless of IFN activity, in line with the intracellular expression of TLR9 and TLR7 at protein level (Fig. 2g, h). Low expression of TLR1, TLR6, and TLR10 was observed in pDCs with no difference among the groups. No expression of TLR2, TLR3, TLR4, TLR5, and TLR8 was found in any of the samples. Additionally, no transcripts for the 14 distinct subtypes of IFN-alpha were detected in the pDCs of any sample. Neither other type I IFN transcripts (IFN-beta, IFN-kappa, IFN-omega) nor transcripts for type III IFNs (IFN-lambda) were found to be expressed in any of the samples (Supplementary Fig. 10). Because it has been suggested that pDCs migrate into inflamed tissues, we checked their expression of chemokine receptors. No significant differences were detected in the expression of chemokines and their receptors apart from the ones described in pDCs of At-Risk individuals, IFN^low^ and IFN^high^ SLE patients (Supplementary Figs. 11 and 12).

### pDCs exhibit immune senescence and stress in autoimmunity

**Fig. 6:**
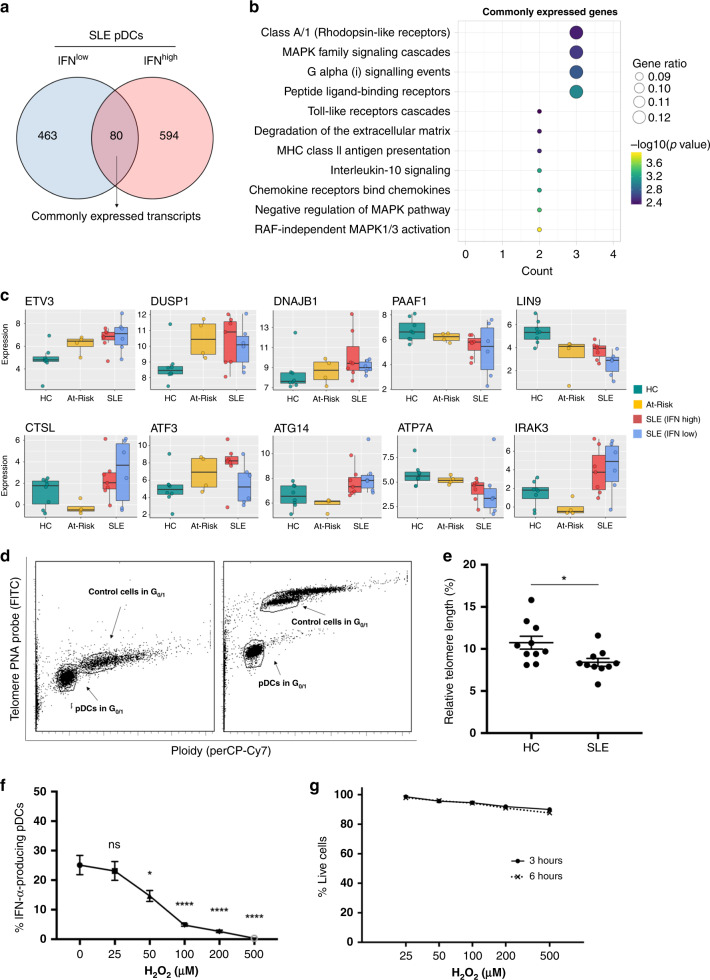
pDCs have features of cellular stress and immune senescence in autoimmunity. **a** Venn diagram showing the number of differentially expressed transcripts (*n* = 80) common to both IFN^low^ and IFN^high^ pDCs from SLE patients compared to pDCs from HC. **b** Reactome Pathway Enrichment in DEGs in differentially expressed genes in IFN^low^ and IFN^high^ pDCs from SLE patients shown in **a**. **c** Expression level of representative genes differentially expressed in both IFN^low^ and IFN^high^ pDCs from SLE patients in comparison with pDCs from HC. **d** Purified pDCs from freshly isolated PBMCs were hybridized without (**d**; right) or with (**d**; left) telomere PNA probe. Gates were set in G_0/1_ phase for both sample cells (pDCs) and tetraploid control cells (1301 cell lines). **e** Determination of the relative telomere length as the ratio between the telomere signal of pDCs purified from HC (*n* = 10) and SLE (*n* = 10) patients and the control cells (1301 cell line) with correction for the DNA index of G_0/1_ cells. **f** Viable cells were defined as double negative when stained for annexin V and 7-AAD; viability was assessed at 3 and 6 h after exposure to H_2_O_2_. One hundred percent of viable cells were defined by the number of cells alive of the non-H_2_O_2_-exposed cells (0 μM) at the two time points. **g** Freshly isolated PBMCs from healthy donors (*n* = 4) were exposed to H_2_O_2_ (0–500 μM) for 15 min. After H_2_O_2_ exposure, cells were washed thoroughly and resuspended in a culture medium before they were stimulated with 2 μM ODN 2216 for 6 h. The production of IFN-α by pDCs was measured in viable cells by intracellular staining. Data are represented as mean ± SEM. *ns* not significant; *P < 0.05; ****P < 0.001. The unpaired two-tailed *t*-test (**e**), 1-way ANOVA (**g**).

In vitro functional assays demonstrated that the decreased secretory and antigen-presenting function was universally observed in pDCs of SLE patients and At-Risk individuals, independently of the IFN activity in their PBMCs. To investigate which biological pathways contribute to this defective phenotype, we, therefore, studied the transcripts differentially expressed in pDCs of both IFN^low^ and IFN^high^ SLE patients compared to those of healthy controls. The little overlap between differentially expressed genes in pDCs of IFN^low^ and IFN^high^ SLE patients was detected—there were 80 shared transcripts (Fig. 6a). Reactome Pathway Enrichment of these transcripts showed that biological processes related to MAPK family signaling, TLR signaling, IL-10 signaling, and chemotaxis were significantly enriched (Fig. 6b). Amongst the 80 shared transcripts, there were upregulated genes involved in cellular senescence and stress (*ATG14*, *ATP7A*, *DNAJB1*), protein degradation in lysosomes (*CTSL*), negative regulation of TLR signaling (*IRAK3*), negative regulation of MAPK signaling (*DUSP1*, *DUSP8*) and negative regulation of non-canonical NF-κB pathway (*ZFP91*), which are all known to inhibit the production of type I IFNs and other pro-inflammatory cytokines (Fig. 6c). Moreover, the shared transcripts included upregulated genes for *CXCL2* and *CCL19* (Supplementary Fig. 5a). For At-Risk individuals, the expression of most of the shared transcripts showed the same trend as in SLE pDCs. In keeping with the functional experiments, we did not see substantial differences in the transcriptomic profile of the 80 shared transcripts when we compared At-Risk pDCs with those of IFN^low^ and IFN^high^ SLE patients (Supplementary Fig. 8). A detailed table of the genes commonly differentially expressed in pDCs of both IFN^low^ and IFN^high^ SLE patients can be found in Supplementary Table 4.

Since the transcriptomic data suggested pathways associated with cellular senescence, we further investigated whether this applied to the pDCs of SLE patients. Increased telomere erosion is known to be related to cellular senescence, a feature that has been found in other immune cells of patients with SLE but has not previously been described in pDCs^45^. We purified pDCs from healthy controls alongside SLE patients, which were then hybridized with telomere PNA probe before analysis by flow cytometry. The relative telomere length was calculated as the ratio between the telomere signal of pDCs and the tetraploid control cells (1301 cell line) with correction for the DNA index of G_0/1_ cells (Fig. 6d). The analysis confirmed that pDCs from SLE patients had shorter telomere length compared to pDCs from age- and sex-matched healthy controls (Fig. 6e).

RNA-sequencing data also suggested that genes related to cellular stress are amongst the 80 shared transcripts. Thus, we next sought to investigate the effect of oxidative stress on type I IFN production in TLR-stimulated pDCs. Freshly isolated PBMCs from healthy donors were exposed to increasing concentrations of H_2_O_2_ (0–500 μM) for 15 min before they were stimulated with ODN 2216. IFN-α production was measured in viable cells using flow cytometry. Cells showed high viability (>90%) indicated by double negativity for Annexin V and 7-AAD even at 6 h after exposure to high concentrations of H_2_O_2_ (Fig. 6f). We observed that oxidative stress—even at low concentrations of H_2_O_2_—negatively regulated TLR-mediated responses in pDCs leading to a gradual loss of their ability to produce IFN-α (Fig. 6g).

### IFN activity is enriched in the skin in preclinical autoimmunity

**Fig. 7:**
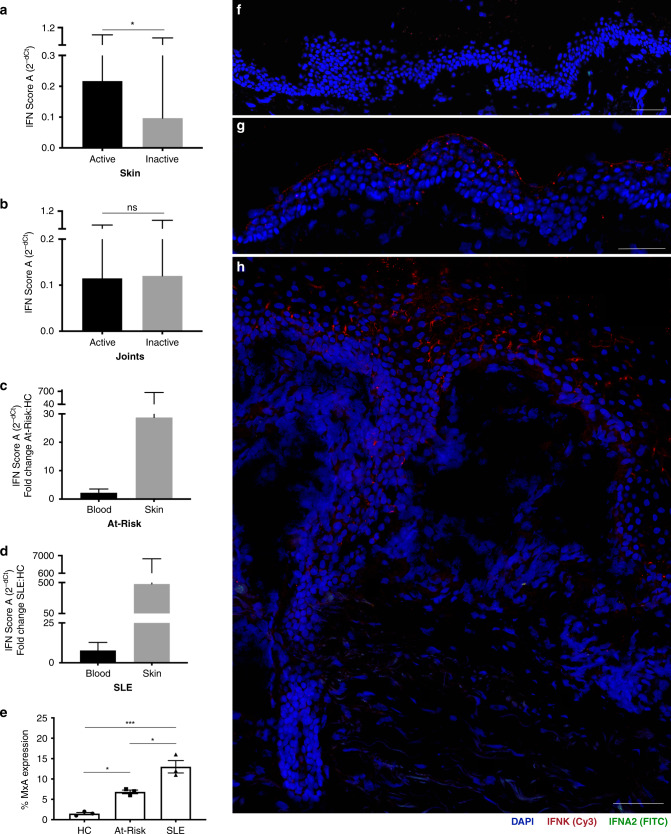
Patients with SLE and At-Risk individuals have a diffuse expression of epidermal *IFNK*. Association of IFN score A with an active and inactive mucocutaneous disease in SLE patients (**a**). Association of IFN score A with an active and inactive musculoskeletal disease in SLE patients (**b**). For graphical representation, mean and SEM were transformed using 2^−dCt^ so that higher expression was shown as a taller bar (**a**, **b**). Fold increase in IFN score A of At-Risk individuals (**c**) in blood (2.21; 95% CI 1.37, 3.53) and skin (28.74; 95% CI 1.29, 639.48) compared to healthy controls. Fold increase in IFN score A of SLE patients (**d**) in blood (7.80; 95% CI 4.75, 12.80) and skin (479.33; 95% CI 39.32, 5842.78) compared to healthy controls. **e** Percentage of MxA expression at the protein level in skin biopsies of healthy controls (HC; *n* = 3), non-lesional skin biopsies of At-Risk individuals (At-Risk; *n* = 3), and non-lesional skin biopsies of SLE patients (SLE; *n* = 3). Skin biopsies were hybridized using RNAscope in situ hybridization technology with custom-designed target probes for *IFNA2* and *IFNK*. Hybridization signals were amplified and detected using TSA Plus fluorescein (FITC) for *IFNA2* and TSA Plus Cyanine 3 (Cy3) for *IFNK*. Nuclei were highlighted using DAPI. Representative in situ hybridization images of **f** healthy control, **g** IFN^high^ At-Risk individual with no clinical or histopathological signs of inflammation, **h** IFN^high^ SLE patient with an active skin lesion. Data are represented as mean ± SEM. Scale bars: 100 µm. ns = not significant; *P < 0.05; ***P < 0.01; ns = not significant. The unpaired two-tailed *t*-test (**a**, **b**), two-way ANOVA (**e**).

Since professional IFN-α-producing cells such as pDCs were functionally impaired in SLE, the source of the aberrant type I IFN production seen in patients had yet to be identified. We compared the level of expression of IFN score A in blood with clinical disease activity in the two most common organ manifestations, defining active disease as BILAG-2004 A or B and inactive disease as BILAG-2004 C-E. We found that IFN score A was associated with mucocutaneous disease activity (fold difference 2.24 (95% CI 1.16–4.34); *P* = 0.017), but not with the musculoskeletal disease (fold difference 0.97 (95% CI 0.44–2.09); *P* = 0.927). For graphical representation, mean and SEM were transformed using 2^−dCt^ so that higher expression was shown as a taller bar (Fig. 7a, b).

Next, we compared the fold increase in IFN score A in paired blood and skin biopsies from At-Risk individuals and SLE patients compared to healthy controls (Fig. 7c, d). We analyzed blood samples from 114 SLE patients, 105 At-Risk individuals, and 49 healthy controls. We also analyzed lesional skin biopsies from 10 SLE patients and non-lesional skin biopsies from 10 At-Risk individuals as well as skin biopsies from 6 healthy controls. In At-Risk individuals compared to healthy controls, the mean fold increase in 2^−dCt^ of IFN score A in blood was 2.21 (95% CI 1.37, 3.53), while in non-lesional skin the fold increase was markedly higher at 28.74 (95% CI 1.29, 639.48). The differential increase in ISG expression in blood and skin was even more extreme in SLE patients compared to healthy controls; in some SLE patients, ISG expression in the skin was more than 5000 times higher than healthy controls. The mean fold increase was 7.80 (95% CI 4.75, 12.80) in blood compared to 479.33 (95% CI 39.32, 5842.78) in the skin. The increased epidermal expression of ISGs was further confirmed by increased protein expression of the interferon-induced GTP-binding protein MxA using immunohistochemistry (Supplementary Fig. 13). Non-lesional skin biopsies from SLE patients (*n* = 3) showed the highest expression of MxA followed by non-lesional skin from At-Risk individuals (*n* = 3) in comparison with skin biopsies from healthy controls (Fig. 7e).

### Diffuse epidermal expression of type I IFNs in autoimmunity

Given the extreme elevation of ISG expression in skin compared to blood, we further analyzed skin biopsies from healthy controls (*n* = 4), SLE patients (*n* = 6), and At-Risk individuals (*n* = 4). Skin biopsies were obtained from active lesions of SLE patients, whilst skin biopsies from At-Risk individuals had no clinical or histopathological signs of inflammation. We performed in situ hybridization using RNAscope technology to visualize the direct expression of type I IFNs transcripts (*IFNK*, *IFNA2*) at a cellular level in all skin biopsies obtained. Representative images of positive and negative controls for in situ hybridization can be seen in Supplementary Fig. 14.

As expected, skin biopsies from healthy controls with minimal IFN score A in blood showed no expression of either *IFNK* or *IFNA2* (Fig. 7f). In contrast, the epidermis of At-Risk individuals with high IFN score A in blood was also characterized by diffuse expression of *IFNK*, although unlike SLE patients, there were no clinical or histopathological features of inflammation (Fig. 7g). Active skin lesions from SLE patients with high IFN score A in blood also demonstrated diffuse expression of *IFNK* in the epidermis (Fig. 7h). Regarding *IFNA*2 expression, we detected expression in the dermis, possibly by fibroblasts as the *IFNA2* signal was located within dense connective tissue. However, notably, we did not see the expression of *IFNK* or *IFNA2* in areas of leucocyte infiltration (Supplementary Fig. 15).

To further evaluate the presence of pDCs in the skin biopsies, staining for BDCA-4 (CD304) was performed using immunofluorescence in non-lesional skin biopsies acquired from healthy controls (*n* = 3), At-Risk individuals (*n* = 3), and patients with SLE (*n* = 3). Although we validated the positivity of the pDCs for the marker in cells isolated from PBMCs, we detected no BDCA-4 positive cells in any of the non-lesional skin biopsies we analyzed (Supplementary Fig. 16).

Collectively, these results indicated that the high IFN activity observed in both non-lesional and lesional skin, including at a preclinical phase, was not mediated by infiltrating hematopoietic immune cells. Instead, the in situ hybridization suggested that non-hematopoietic cells, such as keratinocytes throughout histologically normal skin, were responsible for type I IFN production in scenarios where pDCs produced none.

### Keratinocytes express type I IFN in preclinical autoimmunity

**Fig. 8:**
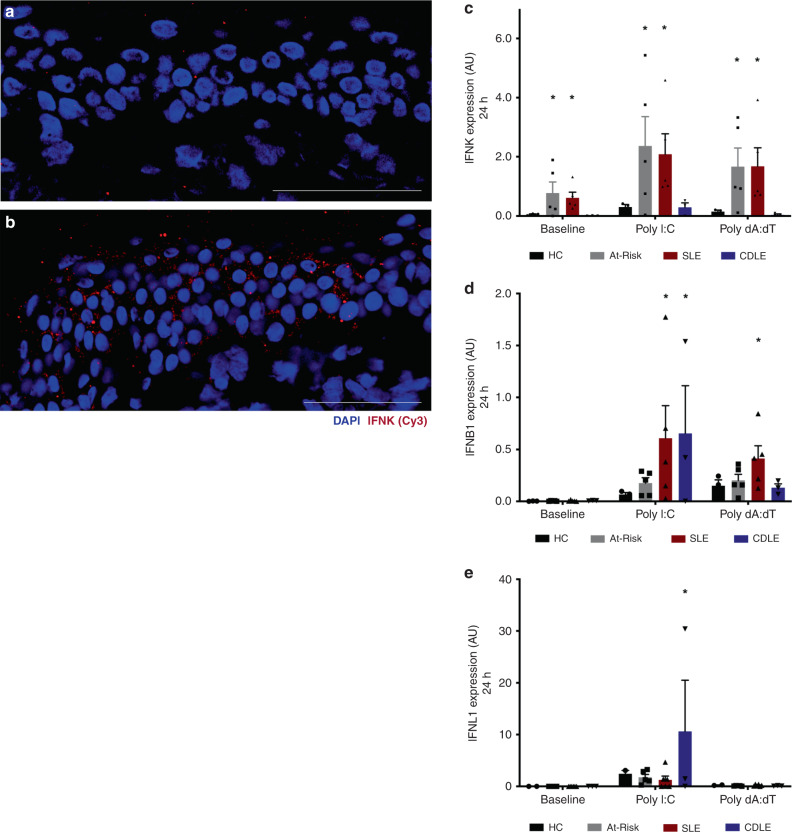
Stimulated keratinocytes have a high expression of type I IFNs in autoimmunity. **a** *IFNK* expression in the epidermis of SLE patient with the inactive disease before UV provocation. **b** *IFNK* expression in the epidermis of the same SLE patient after UV provocation. **c**–**e** Human keratinocytes were isolated from fresh skin biopsies and were then cultured in the absence or presence of Poly I:C (1 μg/mL) or Poly dA:dT (100 ng/mL). The expression level of *IFNK* (**c**), *IFNB1* (**d**), *IFNL1* (**e**) in keratinocytes from healthy controls (HC), At-Risk individuals (At-Risk), SLE patients (SLE), and patients with cutaneous discoid lupus erythematosus (CDLE) after in vitro culture for 24 h. Data are represented as mean ± SEM. Scale bars: 100 µm. *P < 0.05. Two-way ANOVA (**c**–**e**).

To test the response of keratinocytes to a known environmental trigger for cutaneous inflammation, we measured the expression of type I IFN transcripts in the non-lesional skin of a patient with clinically inactive SLE before and after UV provocation in vivo. In a biopsy obtained before UV provocation, there was low *IFNK* expression in the epidermis (Fig. 8a). We then obtained a second biopsy after a standard diagnostic UV provocation using a solar simulator at 1.5× minimal erythema dose on three consecutive days. Following UV provocation, we observed a striking diffuse increase in the expression of *IFNK* in the epidermis using in situ hybridization (Fig. 8b), similar to the expression observed in the non-lesional skin of At-Risk individuals.

In order to confirm this cellular source of IFN, we studied the IFN-producing capacity of keratinocytes in At-Risk individuals and SLE patients in vitro. We isolated human keratinocytes and dermal fibroblasts from non-lesional skin of healthy controls (*n* = 3), At-Risk individuals (*n* = 5), and SLE patients (*n* = 5). Keratinocytes isolated from lesional skin biopsies of patients with cutaneous discoid lupus erythematosus (CDLE), who were ANA-negative and had minimal IFN score A expression in blood, were also used as a disease control (*n* = 3). Cells were cultured and stimulated with TLR3 or RIG-I agonists, Poly(I:C) (1 μg/mL) or Poly(dA:dT) (100 ng/mL), respectively, for 6 and 24 h before the expression of three subtypes of type I IFNs (*IFNK, IFNA2*, and *IFNB1)* as well as type III IFN (*IFNL1)* was measured by qRT-PCR.

At baseline, without exogenous stimulation*, IFNK* was expressed by keratinocytes from At-Risk and SLE skin, but not from healthy controls or CDLE. After either Poly(I:C) or Poly(dA:dT) stimulation, this expression of *IFNK* by At-Risk and SLE keratinocytes was further increased (Fig. 8c). For *IFNB1*, there was no expression at baseline in any sample. However, after stimulation with Poly(I:C) there was a trend to increased expression for At-Risk keratinocytes and a significant increase for keratinocytes from SLE and CDLE patients. *IFNB1* expression was also increased in keratinocytes of SLE patients after Poly(dA:dT) stimulation but not in other conditions (Fig. 8d). In contrast, *IFNL1* expression was only observed in CDLE keratinocytes following Poly(I:C) stimulation but not in the other conditions or following Poly(dA:dT) stimulation (Fig. 8e). Finally, *IFNA2* expression by keratinocytes was not found in any sample or condition.

Matched dermal fibroblasts from the same skin biopsies of these donors (in all four patient groups) showed no expression of *IFNK*, *IFNA2*, *IFNL1* at baseline or after stimulation Poly(I:C) or Poly(dA:dT). Interestingly, only dermal fibroblasts from CDLE patients showed a significant increase in *IFNB1* expression after Poly(dA:dT) stimulation, whilst only a trend was observed for cells from At-Risk individuals and SLE patients (Supplementary Fig. 17).

## Discussion

The importance of type I IFNs in the pathogenesis of human autoimmune connective tissue diseases such as SLE is now generally accepted based on genetic and gene expression data and positive phase III clinical trials of IFN-blocking therapy^46^. But IFNs form a complex system with multiple ligands and receptors with overlapping functions, and all cell types potentially producing and responding to them. This complexity has left many unanswered questions about their cellular source, mechanism of dysregulation, and role in disease initiation and perpetuation. In this study, by including large cohorts of At-Risk individuals with functional, transcriptomic, and tissue data, we have been able to answer some of these questions. Several lines of evidence clearly indicated that, even in At-Risk individuals, pDCs have lost their immunogenic functions, do not correlate with other immunologic or clinical features of the disease, and are not active in tissue. However, simultaneously we found that in histologically normal skin keratinocytes produced type I IFNs with a marked concentration of IFN response at this site in the absence of infiltrating leucocytes. Our results, therefore, locate the IFN response in non-hematopoietic tissues prior to the onset of inflammation.

Plasmablasts, other B cell subsets, and follicular helper-like T cells are expanded in patients with active SLE, upregulating chemokine receptors, and correlating with disease activity^47–50^. In contrast, we reported a marked reduction of circulating pDC numbers even at a preclinical stage. Previous literature speculated that this may indicate the migration of pDCs to inflamed tissues^51,52^. This suggestion was based on the observation of CD123 or CD303 positive cells on immunohistochemistry^53^. Based on the hypothesis that pDC depletion would be effective for SLE, a recent phase I clinical trial evaluated the depletion of BDCA-2 (CD303) positive cells in SLE^54^. However, these markers are not specific for pDCs, as we demonstrated that differentiated monocytes express BDCA-2. Moreover, surface markers do not indicate whether these CD123 or CD303 positive cells produce type I IFNs.

If pDCs in SLE patients passed through an active phase, in which they produced type I IFNs, followed by a senescent phase, then we would expect to see both of these represented in our data by populations of high and low producing pDCs, with variation in these proportions according to the IFN score or disease activity. We did not observe this in any of the ANA-positive populations we studied, even in the At-Risk stage where there is no organ inflammation, no immunosuppressive therapy, and usually no progression to clinical autoimmunity. Instead, we found universally impaired pDC function in SLE and pSS patients and At-Risk individuals with loss of all immunogenic functions and a transcriptomic profile related to senescence. These pDCs did not exhibit any change in chemokine receptor expression, indicating migratory properties, whilst we found no BDCA-4 positive cells in skin biopsies of these groups. The numbers and function of blood pDCs bore no relationship with blood IFN activity, disease activity, or therapy.

Telomere shortening, as we observed in At-Risk and SLE pDCs, could indicate replicative senescence. However, this is unlikely to explain our results. pDCs are a terminally differentiated cell type. In normal circumstances, when they produce type I IFNs, they do not proliferate. Other mechanisms may cause shortening of telomeres and biological aging, such as mild oxidative stress, leading to a similar phenotype^55–57^. Oxidative stress is an important feature of lupus pathology, especially in T cells, contributing to a range of disease-associated changes^58^. Notably, age-induced cellular stress was shown to affect the IFN-α-producing capacity of human pDCs by impairing IRF7 and PI3K pathways^59–61^.

Other recent data had cast doubt on the contribution of pDCs to the type I IFN activity seen in SLE. While attomolar concentrations of IFN-α protein was detected in pDCs of the monogenic interferonopathy STING, this was not seen in SLE samples^62^. However, responses to TLR agonists were not tested^62^. In TREX1-deficient mice, there is a failure to regulate STING-mediated antiviral response leading to aberrant type I IFN production. This IFN response has been shown to initiate in non-hematopoietic cells, similarly to our findings in humans^63^. Moreover, experimental work on lupus-prone mice reported a gradual loss of pDC capacity to produce IFN-α at a late stage of disease^64,65^. Importantly, murine models of chronic viral infection maintained a pool of functionally exhausted pDCs, a similar state to what we describe in chronic autoimmunity in humans^66^.

Recent findings on systemic sclerosis reported the abnormal expression of TLR8 in pDCs that leads to IFN-α production suggesting a key pathological role of RNA-sensing TLR involvement in the establishment of fibrosis^67^. However, in our RNA-sequencing data in pDCs sorted from At-Risk individuals or SLE patients, we could not confirm the positive expression of TLR8 in any of the samples.

Within the pDC population, distinct subsets have been described mediating different immune functions^68^. Single-cell RNA-sequencing data revealed the diversification of human pDCs in response to the influenza virus into three phenotypes (P1-, P2-, P3-pDCs) with distinct transcriptional profiles and functions^69^. In our study, pDCs from SLE patients were mostly similar to the P1-phenotype, which represented the conventional secretory function and morphology of pDCs. This is consistent with our finding that SLE pDCs demonstrated decreased ability to induce CD4^+^CD25^high^FoxP3^+^ T cells, the numbers and function of which are known to be impaired in patients with active SLE^70–72^. Additionally, SLE pDCs differentially expressed genes that are well-known to be involved in cellular senescence and stress, negative regulation of TLR and MAPK pathways as well as IL-10 signaling downstream, which can inhibit cytokine production and survival of pDCs^73–75^. Similar to our findings in pDCs, kidney infiltrating T cells in murine lupus have recently been shown to exhibit an exhausted transcriptional signature and phenotype^76^.

Cutaneous disease activity in SLE had previously been shown to associate with a systemic ISG signature^77^. In our work, we extend this finding by analyzing paired blood and skin samples to demonstrate that the IFN score is markedly enriched in the skin, even in the absence of clinical inflammation and in At-Risk individuals. This is therefore suggestive of local production of IFN in the skin prior to the onset of systemic disease.

IFN-κ is predominantly produced by human keratinocytes with pleiotropic effects similar to IFN-α/β^78^. In SLE patients, keratinocytes have been implicated in the pathogenesis of skin injury by undergoing apoptosis or necrosis and eventually releasing autoantigens^79^. Previous studies demonstrated that keratinocytes from patients with cutaneous lupus erythematosus presented increased production of IL-6 compared to healthy controls, with type I IFNs enhancing this process^80^. In addition, *IFNK* expression was reported to be significantly increased in lesional skin of patients with cutaneous lupus erythematosus related to photosensitivity^81^. By visualizing the direct expression of type I IFN transcripts in human skin biopsies, we confirmed and extended these findings. Diffuse expression of *IFNK* was seen not only in the epidermis of lesional skin of SLE patients but, more importantly, in the epidermis of non-lesional skin of ANA-positive At-Risk individuals with high IFN activity in blood, who had no clinical or histopathological features of inflammation. The effect of UV light on inducing IFN-κ production by keratinocytes has previously been demonstrated in vitro^81^. Here, we demonstrated that UV provocation in vivo induced higher expression of *IFNK* in the epidermis of SLE patients with inactive disease. Notably, in vitro culture of keratinocytes showed high expression of *IFNK* at baseline, whilst stimulation with TLR3 and RIG-I agonists enhanced *IFNK* expression, even in At-Risk individuals. Further, the types of IFN (kappa, beta, lambda) produced varied between systemic and discoid lupus as well as different cellular stimuli. These results, therefore, indicate the production of type I IFNs by non-hematopoietic cells in the absence of production by pDCs or tissue leucocytes early in the initiation of autoimmunity and in a disease-specific manner.

In summary, while the importance of type I IFN in SLE is undeniable, the reasons for the failure of normal regulation of its production have never been clear. Our results indicate that tissues such as the skin are not mere passive targets for leucocyte-mediated immune processes but have an active role in generating an IFN response, which dominates over inert pDCs. Immune functions of non-hematopoietic tissues may have a role in determining the clinical presentation of these heterogeneous diseases. They may help to explain the resistance of tissue inflammation to commonly used therapies that target leucocytes, and instead point to therapeutic targets that lie outside the conventional immune system. Further study of these tissues may offer insights into the early events in the initiation of autoimmune disease and therefore how these might be targeted for disease prevention.

## Methods

### Patients and healthy volunteers

Peripheral blood and skin biopsies were obtained from healthy individuals and patients from different disease groups (SLE, pSS, At-Risk). Patients were recruited based on 2012 SLICC classification criteria for SLE, 2016 ACR/EULAR classification criteria for pSS, while At-Risk individuals were classified as ANA-positive, ≤1 SLE clinical criterion, symptom duration <12 months, and treatment-naive. Supplementary Table 1 summarizes the characteristics and treatment of SLE patients. Ethical approval was provided by Leeds East–National Research Ethics Committee (REC 10/H1306/88) and all participants gave written informed consent before participation.

### Isolation of human peripheral blood cells

Human PBMCs were separated from whole blood by a density gradient centrifugation method using Leucosep tubes (Greiner Bio-One). pDCs were purified from freshly isolated PBMCs by negative selection using the Diamond Plasmacytoid Dendritic Cell Isolation Kit II (Miltenyi Biotec). Naive CD4^+^ T cells were purified by negative selection using the Naive CD4^+^ T Cell Isolation Kit II (Miltenyi Biotec). Pre-enriched pDCs were sorted using an antibody to BDCA-4 (Miltenyi Biotec). Cell sorting was carried out at the SCIF Flow Cytometry and Imaging Facility of the Wellcome Trust Brenner Building, University of Leeds, with a BD Influx 6 Way Cell Sorter (BD Biosciences).

### Culture of human peripheral blood cells

Cells were cultured in RPMI medium 1640 with GlutaMAX supplement (ThermoFisher Scientific) containing 10% (vol/vol) FBS and 100 U/mL penicillin/streptomycin. For cytokine production, PBMCs were stimulated with 2 μM class A CpG (ODN 2216; Miltenyi Biotec) or 2 μM ORN R-2336 (Miltenyi Biotec). For pDC/T-cell co-cultures, the cells were purified as described above (isolation of human peripheral blood cells). Purified pDCs (1 × 105) were cultured with autologous or allogeneic naive CD4^+^ T cells (5 × 105) for 5 days in the absence or presence of anti-CD3/CD28 beads (T-cell activation/expansion kit; Miltenyi Biotec) at a bead-to-cell ratio of 1:2. Cytokine production was measured by intracellular staining.

### Flow cytometry

For cell surface staining, fluorochrome-conjugated monoclonal antibodies against human CD3, CD4, CD19, CD14, CD56, CD11c, HLA-DR, CD123, CD303, CD304, CD85g, CD85j, CD69, CD25 (Miltenyi Biotec), CD317 (BioLegend) and isotype controls were used. For intracellular staining, cells were first stained for surface markers and then fixed and permeabilized using the Intracellular Fixation & Permeabilization Buffer Set (eBioscience). Fluorochrome-conjugated monoclonal antibodies against human IFN-α, TNF, IL-6, IFN-γ, IL-10 (Miltenyi Biotec), TLR9 (BD Biosciences), TLR7 (R&D Systems), and isotype controls were used. For FoxP3 (Miltenyi Biotec) intracellular staining, cells were first stained for surface markers and then fixed and permeabilized using the FoxP3 Staining Buffer Set (Miltenyi Biotec). Cell proliferation was measured using the CellTrace Violet Cell Proliferation kit (ThermoFisher Scientific) according to the manufacturer’s instructions. Flow cytometry was performed on LSRII (BD Biosciences) or Cytoflex S (Beckman Coulter) and the data were analyzed using FACS DiVA (BD Biosciences) or CytExpert (Beckman Coulter) software. A detailed table of antibodies used for flow cytometry can be seen in Supplementary Table 8.

### Quantification of gene expression in peripheral blood

Total RNA was extracted from freshly isolated PBMCs using the Total RNA Purification Kit (Norgen Biotek) according to the manufacturer’s instructions. RNA was reverse transcribed to cDNA using the Fluidigm Reverse Transcription Master Mix buffer and gene expression was measured by TaqMan assays (Applied Biosystems, Invitrogen). The relative expression of specific transcripts (*ISG15, IFI44, IFI27, CXCL10, RSAD2, IFIT1, IFI44L, CCL8, XAF1, GBP1, IRF7, CEACAM1*) was normalized with respect to the internal standard (*PP1A*). IFN score A was calculated based on factor analysis as the median dCT of these transcripts^8^.

### RNA-sequencing data generation

RNA from sorted pDCs was extracted using PicoPure RNA Isolation Kit (ThermoFisher Scientific) and quantified using the Qubit RNA HS Assay Kit (ThermoFisher Scientific). RNA libraries were made by using SMART-Seq V4 ultra low Input RNA Kit (Takara Bio USA) and Nextera XT DNA Library Preparation Kit (Illumina) for NGS sequencing. Indexed sequencing libraries were pooled and sequenced on a single lane on the HiSeq 3000 instrument as 151 bp paired-end reads. Pooled sequence data was then demultiplexed using Illumina bcl2fastq software allowing no mismatches in the read index sequences.

### RNA-sequencing data processing and analysis

Raw paired-end sequence data in Fastq format was initially analyzed using FastQC software in order to identify potential issues with data quality. Cutadapt software was then used to remove poor-quality bases (Phred quality score <20) and contaminating technical sequences from raw sequenced reads. Contaminating technical sequences identified at the initial QC stage were as follows:

CTGTCTCTTATA – Next Era Transposase Sequence

GTATCAACGCAGAGTACT– SmartSeq Oligonucleotide Sequence

dT30 – SmartSeq 3′ CDS Primer II sequence

Reads trimmed to fewer than 30 nucleotides and orphaned mate-pair reads were discarded to minimize alignment errors downstream.

Reads were aligned to human hg38 analysis set reference sequences, obtained from UCSC database^82^ using splicing-aware STAR aligner^83^ for RNA-Sequencing data. STAR aligner was run in 2-pass mode, with known splice junctions supplied in GTF file format, obtained from the hg38 RefSeq gene annotation table from the UCSC database using Table Browser tool^84^. The resulting alignments in the BAM file format were checked for quality using QualiMap software^85^ and Picard tools^86^. Picard tools were used to mark PCR/Optical duplicate alignments. Custom code was used to filter out contaminating ribosomal RNA alignments, using ribosomal RNA coordinates for hg38 analysis set reference obtained using the UCSC Table Browser tool. The final alignment files were sorted and indexed using Samtools software^87^ and visualized using IGV browser^88^.

Bioconductor R package RSubread^89^ was used to extract raw sequenced fragment counts per transcript using the RefSeq hg38 transcript annotation set, as before. Paired-end reads were counted as a single fragment and multi-mapping read pairs were counted as a fraction of all equivalent alignments. Raw count data were normalized for library size differences using median ratio method^90^, as implemented in DESeq2 R Bioconductor package^91^. DESeq2 was also used to perform additional data QC steps and differential expression analyses. Differentially expressed gene expression was visualized as clustered heatmaps using Pheatmap R package^92^ using log-transformed normalized gene expression values as input. Gene functional and pathway enrichment analyses were performed using R Bioconductor packages clusterProfiler^93^ and ReactomePA^94^. Additionally, KEGG^95^ pathways were visualized using Pathview package^96^.

### Measurement of relative telomere length

Relative telomere length was measured using the Telomere PNA Kit/FITC for Flow Cytometry (Agilent) according to the manufacturer’s protocol. Briefly, on a single-cell suspension consisting of purified pDCs and control cells (1301 cell line; Sigma-Aldrich), the sample DNA was denatured for 10 min at 82 °C either in the presence of hybridization solution without probe or in hybridization solution containing fluorescein-conjugated PNA telomere probe. Then hybridization took place in the dark at room temperature overnight. The sample was then resuspended in an appropriate buffer for further flow cytometric analysis. The data obtained were used for the determination of the relative telomere length as the ratio between the telomere signal of each sample (pDCs) and the control cell (1301 cell line) with correction for the DNA index of G_0/1_ cells.

### Oxidative stress assay

Freshly isolated PBMCs from healthy donors were exposed to H_2_O_2_ (0–500 μM) for 15 min. After exposure, cells were washed thoroughly and resuspended at 1 × 10^6^ in a culture medium before they were stimulated with 2 μM ODN 2216 (Miltenyi Biotech) for 6 h. The production of IFN-α (Miltenyi Biotec) by pDCs was measured by intracellular staining as described above. The viability of pDCs was assessed by staining the cells for Annexin V (Miltenyi Biotec) and 7-AAD (Miltenyi Biotec) before data analysis using flow cytometry.

### Measurement of antigen uptake

pDCs were isolated from PBMCs (Miltenyi Biotec) and were then cultured in RPMI medium 1640 with GlutaMAX supplement (ThermoFisher Scientific) containing 10% (vol/vol) FBS and 100 U/mL penicillin/streptomycin in a 96-well plate. Purified pDCs were cultured with or without 10 μg/mL DQ Ovalbumin (Molecular Probes). After 18 h, the cells were collected and then washed twice before data were analyzed using flow cytometry. Samples from healthy controls (*n* = 3), At-Risk individuals (*n* = 3), and SLE patients (*n* = 3) were used.

### UV provocation

UV provocation was performed based on a published protocol designed for use in clinical trials^97,98^. Briefly, a solar simulator was used in routine clinical practice, which replicated the protocol of UV-A and UV-B provocation in a single exposure. On day 1, four 1.5 cm^2^ areas of skin were exposed to solar simulated radiation depending on skin type; 4, 8, 12, 16 J/cm^2^ for skin types I and II, and 6, 12, 18, 24 J/cm^2^ for skin types III–VI. On day 2, the minimal erythema dose was then determined. A 10 cm^2^ non-sun exposed area of skin was exposed to minimal erythema dose × 1.5 on three consecutive days. A biopsy of the pre-exposed and exposed area of skin was obtained when a reaction was seen clinically (mean time to a positive reaction to provocation was 7 (±6) days, and rarely more than 14 days).

### Tissue section

Skin biopsies were obtained from healthy individuals and patients, then snap-frozen in liquid nitrogen within 5 min, embedded in OCT, and stored in a −80 °C freezer. Fresh frozen skin biopsies were cryosectioned to 10–20 μM, placed on superfrost plus slides (ThermoFisher Scientific), and used for in situ hybridization.

### In situ hybridization and fluorescence microscopy

In situ hybridization of type I IFNs transcripts in skin samples was performed using RNAscope Multiplex Fluorescent Reagent Kit v2 (Advanced Cell Diagnostics) according to manufacturer’s instructions. Briefly, cryosections were fixed and dehydrated before exposure to hydrogen peroxide and protease treatment followed by hybridization for 2 h with custom-designed target probes (*IFNA2*, *IFNK*). Appropriate positive and negative controls were provided by the manufacturer. Hybridization signals were amplified and detected using TSA Plus fluorescein and TSA Plus Cyanine 3 (Perkin Elmer) according to the manufacturer’s protocol. Nuclei were highlighted using 4′,6-diamidino-2-phenylindole (DAPI). Slides were mounted using Prolong Gold Antifade mounting medium (ThermoFischer Scientific) and dried overnight in the dark at 4 ^o^C. Images were acquired on a Nikon A1R confocal laser scanning microscope system at ×20–×40 magnification. Images were analyzed in the Nikon NIS Elements software.

### In situ immunohistochemistry

Immunohistochemistry was utilized to detect the protein expression of MxA protein. Human skin biopsies from healthy controls (*n* = 3), At-Risk individuals (*n* = 3), and SLE patients (*n* = 3) were frozen unfixed in optimal cutting temperature (OCT) compound (Tissue-Tek) for cryosectioning and stored at 80 °C for future use. 5 µm-thick-frozen sections were cut using a Leica CM3050S cryostat (Leica Microsystems Ltd) and mounted on Polysine (poly-l-lysine coated) slides (ThermoFisher Scientific). Just before use, sections were defrosted at room temperature for 3–5 min, fixed in ice-cold acetone for 7–10 min, and subsequently rehydrated in 1x PBS for 5 min. Sections were then permeabilized in a solution of 1× PBS containing 0.05% Tween-20 (Sigma-Aldrich) for 10 min. Endogenous peroxidase activity was blocked by incubation in 3% H_2_O_2_ in (vol/vol) methanol/de-ionized water for 30 min at room temperature followed by a brief rinse in PBS. Nonspecific bindings were blocked using 2% Bovine Serum Albumin (Sigma) in PBS for 30 min at room temperature, followed by incubation with 1:1000 human anti-MxA antibody (rabbit polyclonal, ab95926) in 1% normal donkey serum (Sigma-Aldrich) in PBS overnight at 4 °C in a moist chamber to avoid dehydration. After washing twice in PBS for 5 min, with an intermediate wash in 0.05% solution of Tween-20 in PBS, sections were incubated with anti-rabbit (SAB3700930) polyclonal biotin-conjugated secondary antibody (Sigma-Aldrich) diluted 1:100 in 1% normal donkey serum in PBS for 30 min at RT. Sections were washed as described above following by the application of Streptavidin peroxidase (Sigma-Aldrich, S2438) at a dilution of 1:100 in PBS for 30 min at RT. After a washing procedure, antibody binding was visualized by the addition of the peroxidase substrate, 3-amino-9-ethylcarbazole (AEC Substrate Kit SK-4200, Vector Laboratories). Chromogen formation was observed under a light microscope, and the reaction was stopped by placing the slides in distilled water when sufficient color was developed. Sections were dried and mounted with Aquamount (VWR International). Potential nonspecific binding due to the secondary antibody was checked by the replacement of the primary antibodies with 1% normal donkey serum (negative control). The resulting staining was examined and photographed using a Nikon Eclipse 80i light microscope with a Nikon ACT-2U photographic system (Nikon).

### In situ immunofluorescence

Slides containing frozen sections of skin biopsies from healthy controls (*n* = 3), At-Risk individuals (*n* = 3), and SLE patients (*n* = 3) were defrosted at room temperature and fixed in ice-cold methanol for 10 min at −20 ^o^C and rehydrated in PBS for 5 min. Sections were then blocked in 10% fetal calf serum (FBS) blocking solution in 1 % PBS for 90 min at room temperature. This step was followed by incubation with rabbit monoclonal (ab81321) to BDCA-4 (Abcam) at 1:20 dilution of 1% FBS overnight at 4 °C. After washing twice in PBS for 5 min and an intermediate wash in PBS/Tween-20 (0.05%), sections were incubated with a PE-conjugated donkey anti-rabbit secondary antibody (Jackson ImmunoResearch Laboratories) at a dilution of 1:100 with 1% FBS, for 1 h at room temperature. Slides were washed as above and were dried and mounted in Vectashield Mounting Medium with 4,6-diamino-2-phenylindole (DAPI) (Vector Laboratories). Finally, sections were viewed with a Leica DMIRB/E fluorescence microscope (Leica Microsystems Ltd).

### Culture of human keratinocytes and dermal fibroblasts

Human keratinocytes and dermal fibroblasts were isolated from 3 mm punch skin biopsies. The epidermal component of the biopsy was placed in a T75 flask and cultured at 37 °C in low glucose DMEM (Fischer Scientific) containing 10% (vol/vol) FBS (Fischer Scientific) and 1% penicillin/streptomycin. The cells were passaged and sub-cultured into a growth medium (PromoCell) for continuous culture. Keratinocytes and dermal fibroblasts were passaged and plated in 24-well plates for subsequent stimulation. At 90% confluence, cells were either untreated or treated with 1 μg/ml Poly I:C (InvivoGen) or 100 ng/mL Poly dA:dT (InvivoGen) for 6 or 24 h.

### Quantitative RT-PCR for keratinocytes and dermal fibroblasts

RNA was extracted from keratinocytes and dermal fibroblasts using Quick-RNA MiniPrep kit (Zymo Research) according to the manufacturer’s instructions. Extracted RNA was reverse transcribed using the First Strand cDNA Synthesis kit (ThermoFisher Scientific). The cDNA was then used in a qRT-PCR assay using QuantiFast SYBR Green PCR kit (Qiagen). For the assay, the following QuantiTech primers were used: *IFNK* (QT00197512; Qiagen), *IFNB1* (QT00203763; Qiagen), *IFNL1* (QT00222495; Qiagen), *IFNA2* (QT00212527; Qiagen), *U6snRNA* (forward—5′-CTCGCTTCGGCAGCACA-3′; reverse—5′-AACGCTTCACGAATTTGC-3′; Sigma-Aldrich). For gene expression analysis, ddCt method was used and all samples were normalized to the housekeeping gene (*U6snRNA)*.

### Statistical analysis

Statistical analyses were carried out with Prism software (GraphPad). Continuous variables were compared using either Student’s *t*-test or ANOVA followed by pairwise Tukey tests. Pearson’s correlation was used for associations. A *P*-value of ≤ 0.05 was considered significant (ns, not significant; *P < 0.05; **P < 0.01; ***P < 0.001; ****P < 0.0001). In all figures, error bars indicate SEM unless it is stated otherwise.

### Reporting summary

Further information on research design is available in the Nature Research Reporting Summary linked to this article.

## Supplementary information

Supplementary Information

Reporting Summary

## Data Availability

RNA sequence data have been deposited in BioProject under accession code PRJNA645252. All other data are available in the article and Supplementary files or from the corresponding author upon reasonable request. Raw data are provided as a Source Data file.

## Source data

Source Data

## References

[1] Psarras A, Emery P, Vital EM (2017). Type I interferon-mediated autoimmune diseases: pathogenesis, diagnosis and targeted therapy. Rheumatology.

[2] Crow MK (2014). Type I interferon in the pathogenesis of lupus. J. Immunol..

[3] Deng Y, Tsao BP (2010). Genetic susceptibility to systemic lupus erythematosus in the genomic era. Nat. Rev. Rheumatol..

[4] Sigurdsson S (2005). Polymorphisms in the tyrosine kinase 2 and interferon regulatory factor 5 genes are associated with systemic lupus erythematosus. Am. J. Hum. Genet..

[5] Niewold TB (2012). IRF5 haplotypes demonstrate diverse serological associations which predict serum interferon alpha activity and explain the majority of the genetic association with systemic lupus erythematosus. Ann. Rheum. Dis..

[6] Salloum R (2010). Genetic variation at the IRF7/PHRF1 locus is associated with autoantibody profile and serum interferon-alpha activity in lupus patients. Arthritis Rheum..

[7] Robinson T (2011). Autoimmune disease risk variant of IFIH1 is associated with increased sensitivity to IFN-alpha and serologic autoimmunity in lupus patients. J. Immunol..

[8] El-Sherbiny YM (2018). A novel two-score system for interferon status segregates autoimmune diseases and correlates with clinical features. Sci. Rep..

[9] Ytterberg SR, Schnitzer TJ (1982). Serum interferon levels in patients with systemic lupus erythematosus. Arthritis Rheum..

[10] Bennett L (2003). Interferon and granulopoiesis signatures in systemic lupus erythematosus blood. J. Exp. Med..

[11] Baechler EC (2003). Interferon-inducible gene expression signature in peripheral blood cells of patients with severe lupus. Proc. Natl Acad. Sci. USA.

[12] Md Yusof MY (2018). Prediction of autoimmune connective tissue disease in an at-risk cohort: prognostic value of a novel two-score system for interferon status. Ann. Rheum. Dis..

[13] Cherian TS (2012). Brief Report: IRF5 systemic lupus erythematosus risk haplotype is associated with asymptomatic serologic autoimmunity and progression to clinical autoimmunity in mothers of children with neonatal lupus. Arthritis Rheum..

[14] Niewold TB, Hua J, Lehman TJ, Harley JB, Crow MK (2007). High serum IFN-alpha activity is a heritable risk factor for systemic lupus erythematosus. Genes Immun..

[15] Aberle T (2017). Clinical and serologic features in patients with incomplete lupus classification versus systemic lupus erythematosus patients and controls. Arthritis Care Res..

[16] Lu R (2016). Dysregulation of innate and adaptive serum mediators precedes systemic lupus erythematosus classification and improves prognostic accuracy of autoantibodies. J. Autoimmun..

[17] Feng X (2006). Association of increased interferon-inducible gene expression with disease activity and lupus nephritis in patients with systemic lupus erythematosus. Arthritis Rheum..

[18] Chiche L (2014). Modular transcriptional repertoire analyses of adults with systemic lupus erythematosus reveal distinct type I and type II interferon signatures. Arthritis Rheumatol..

[19] Kirou KA (2005). Activation of the interferon-alpha pathway identifies a subgroup of systemic lupus erythematosus patients with distinct serologic features and active disease. Arthritis Rheum..

[20] Siegal FP (1999). The nature of the principal type 1 interferon-producing cells in human blood. Science.

[21] Swiecki M, Colonna M (2015). The multifaceted biology of plasmacytoid dendritic cells. Nat. Rev. Immunol..

[22] Blasius AL, Beutler B (2010). Intracellular toll-like receptors. Immunity.

[23] Villadangos JA, Young L (2008). Antigen-presentation properties of plasmacytoid dendritic cells. Immunity.

[24] Cella M, Facchetti F, Lanzavecchia A, Colonna M (2000). Plasmacytoid dendritic cells activated by influenza virus and CD40L drive a potent TH1 polarization. Nat. Immunol..

[25] Yu CF (2010). Human plasmacytoid dendritic cells support Th17 cell effector function in response to TLR7 ligation. J. Immunol..

[26] Martin-Gayo E, Sierra-Filardi E, Corbi AL, Toribio ML (2010). Plasmacytoid dendritic cells resident in human thymus drive natural Treg cell development. Blood.

[27] Chen W, Liang X, Peterson AJ, Munn DH, Blazar BR (2008). The indoleamine 2,3-dioxygenase pathway is essential for human plasmacytoid dendritic cell-induced adaptive T regulatory cell generation. J. Immunol..

[28] Boasso A (2007). HIV inhibits CD4+ T-cell proliferation by inducing indoleamine 2,3-dioxygenase in plasmacytoid dendritic cells. Blood.

[29] Vermi W (2003). Recruitment of immature plasmacytoid dendritic cells (plasmacytoid monocytes) and myeloid dendritic cells in primary cutaneous melanomas. J. Pathol..

[30] Gerlini G (2007). Plasmacytoid dendritic cells represent a major dendritic cell subset in sentinel lymph nodes of melanoma patients and accumulate in metastatic nodes. Clin. Immunol..

[31] Conrad C (2012). Plasmacytoid dendritic cells promote immunosuppression in ovarian cancer via ICOS costimulation of Foxp3(+) T-regulatory cells. Cancer Res..

[32] Vincent IE (2011). Hepatitis B virus impairs TLR9 expression and function in plasmacytoid dendritic cells. PLoS ONE.

[33] Lo CC (2012). HIV delays IFN-alpha production from human plasmacytoid dendritic cells and is associated with SYK phosphorylation. PLoS ONE.

[34] Zuniga EI, Liou LY, Mack L, Mendoza M, Oldstone MB (2008). Persistent virus infection inhibits type I interferon production by plasmacytoid dendritic cells to facilitate opportunistic infections. Cell Host Microbe.

[35] Gilliet M, Cao W, Liu YJ (2008). Plasmacytoid dendritic cells: sensing nucleic acids in viral infection and autoimmune diseases. Nat. Rev. Immunol..

[36] Bave U (2003). Fc gamma RIIa is expressed on natural IFN-alpha-producing cells (plasmacytoid dendritic cells) and is required for the IFN-alpha production induced by apoptotic cells combined with lupus IgG. J. Immunol..

[37] Means TK (2005). Human lupus autoantibody-DNA complexes activate DCs through cooperation of CD32 and TLR9. J. Clin. Invest..

[38] Eloranta ML (2009). Regulation of the interferon-alpha production induced by RNA-containing immune complexes in plasmacytoid dendritic cells. Arthritis Rheum..

[39] Jin O (2008). Systemic lupus erythematosus patients have increased number of circulating plasmacytoid dendritic cells, but decreased myeloid dendritic cells with deficient CD83 expression. Lupus.

[40] Blanco P, Palucka AK, Gill M, Pascual V, Banchereau J (2001). Induction of dendritic cell differentiation by IFN-alpha in systemic lupus erythematosus. Science.

[41] Kwok SK (2008). Dysfunctional interferon-alpha production by peripheral plasmacytoid dendritic cells upon Toll-like receptor-9 stimulation in patients with systemic lupus erythematosus. Arthritis Res. Ther..

[42] Murayama G (2017). Enhanced IFN-alpha production is associated with increased TLR7 retention in the lysosomes of palasmacytoid dendritic cells in systemic lupus erythematosus. Arthritis Res. Ther..

[43] Cella M (1999). Plasmacytoid monocytes migrate to inflamed lymph nodes and produce large amounts of type I interferon. Nat. Med..

[44] Gary-Gouy H, Lebon P, Dalloul AH (2002). Type I interferon production by plasmacytoid dendritic cells and monocytes is triggered by viruses, but the level of production is controlled by distinct cytokines. J. Interferon Cytokine Res..

[45] Haque S (2013). Shortened telomere length in patients with systemic lupus erythematosus. Arthritis Rheum..

[46] Morand EF (2020). Trial of anifrolumab in active systemic lupus erythematosus. N. Engl. J. Med..

[47] Jacobi AM (2010). HLA-DRhigh/CD27high plasmablasts indicate active disease in patients with systemic lupus erythematosus. Ann. Rheum. Dis..

[48] Arce E (2001). Increased frequency of pre-germinal center B cells and plasma cell precursors in the blood of children with systemic lupus erythematosus. J. Immunol..

[49] Wang S (2018). IL-21 drives expansion and plasma cell differentiation of autoreactive CD11c(hi)T-bet(+) B cells in SLE. Nat. Commun..

[50] Choi JY (2015). Circulating follicular helper-like T cells in systemic lupus erythematosus: association with disease activity. Arthritis Rheumatol..

[51] Tucci M (2008). Glomerular accumulation of plasmacytoid dendritic cells in active lupus nephritis: role of interleukin-18. Arthritis Rheum..

[52] Vermi W (2009). Cutaneous distribution of plasmacytoid dendritic cells in lupus erythematosus. Selective tropism at the site of epithelial apoptotic damage. Immunobiology.

[53] Tomasini D (2010). Plasmacytoid dendritic cells: an overview of their presence and distribution in different inflammatory skin diseases, with special emphasis on Jessner’s lymphocytic infiltrate of the skin and cutaneous lupus erythematosus. J. Cutan. Pathol..

[54] Furie R (2019). Monoclonal antibody targeting BDCA2 ameliorates skin lesions in systemic lupus erythematosus. J. Clin. Invest..

[55] von Zglinicki T, Saretzki G, Docke W, Lotze C (1995). Mild hyperoxia shortens telomeres and inhibits proliferation of fibroblasts: a model for senescence?. Exp. Cell Res..

[56] Saretzki G, Von Zglinicki T (2002). Replicative aging, telomeres, and oxidative stress. Ann. N Y Acad. Sci..

[57] Saretzki G, Murphy MP, von Zglinicki T (2003). MitoQ counteracts telomere shortening and elongates lifespan of fibroblasts under mild oxidative stress. Aging Cell.

[58] Perl A (2013). Oxidative stress in the pathology and treatment of systemic lupus erythematosus. Nat. Rev. Rheumatol..

[59] Shodell M, Siegal FP (2002). Circulating, interferon-producing plasmacytoid dendritic cells decline during human ageing. Scand. J. Immunol..

[60] Stout-Delgado HW, Yang X, Walker WE, Tesar BM, Goldstein DR (2008). Aging impairs IFN regulatory factor 7 up-regulation in plasmacytoid dendritic cells during TLR9 activation. J. Immunol..

[61] Bokov A, Chaudhuri A, Richardson A (2004). The role of oxidative damage and stress in aging. Mech. Ageing Dev..

[62] Rodero MP (2017). Detection of interferon alpha protein reveals differential levels and cellular sources in disease. J. Exp. Med..

[63] Gall A (2012). Autoimmunity initiates in nonhematopoietic cells and progresses via lymphocytes in an interferon-dependent autoimmune disease. Immunity.

[64] Liao X (2015). Cutting edge: plasmacytoid dendritic cells in late-stage lupus mice defective in producing IFN-alpha. J. Immunol..

[65] Zhou Z (2016). Phenotypic and functional alterations of pDCs in lupus-prone mice. Sci. Rep..

[66] Macal M (2018). Self-renewal and toll-like receptor signaling sustain exhausted plasmacytoid dendritic cells during chronic viral infection. Immunity.

[67] Ah Kioon MD (2018). Plasmacytoid dendritic cells promote systemic sclerosis with a key role for TLR8. Sci. Transl. Med.

[68] Zhang H (2017). A distinct subset of plasmacytoid dendritic cells induces activation and differentiation of B and T lymphocytes. Proc. Natl Acad. Sci. USA.

[69] Alculumbre SG (2018). Diversification of human plasmacytoid predendritic cells in response to a single stimulus. Nat. Immunol..

[70] Miyara M (2005). Global natural regulatory T cell depletion in active systemic lupus erythematosus. J. Immunol..

[71] Comte D (2017). Brief report: CD4+ T cells from patients with systemic lupus erythematosus respond poorly to exogenous interleukin-2. Arthritis Rheumatol..

[72] Kammer GM (2005). Altered regulation of IL-2 production in systemic lupus erythematosus: an evolving paradigm. J. Clin. Invest.

[73] Kobayashi K (2002). IRAK-M is a negative regulator of Toll-like receptor signaling. Cell.

[74] Agrawal A (2007). Altered innate immune functioning of dendritic cells in elderly humans: a role of phosphoinositide 3-kinase-signaling pathway. J. Immunol..

[75] Duramad O (2003). IL-10 regulates plasmacytoid dendritic cell response to CpG-containing immunostimulatory sequences. Blood.

[76] Tilstra JS (2018). Kidney-infiltrating T cells in murine lupus nephritis are metabolically and functionally exhausted. J. Clin. Investig..

[77] Braunstein I, Klein R, Okawa J, Werth VP (2012). The interferon-regulated gene signature is elevated in subacute cutaneous lupus erythematosus and discoid lupus erythematosus and correlates with the cutaneous lupus area and severity index score. Br. J. Dermatol..

[78] LaFleur DW (2001). Interferon-kappa, a novel type I interferon expressed in human keratinocytes. J. Biol. Chem..

[79] Deng GM, Tsokos GC (2015). Pathogenesis and targeted treatment of skin injury in SLE. Nat. Rev. Rheumatol..

[80] Stannard JN (2017). Lupus skin is primed for IL-6 inflammatory responses through a keratinocyte-mediated autocrine type I interferon loop. J. Invest. Dermatol..

[81] Sarkar MK (2018). Photosensitivity and type I IFN responses in cutaneous lupus are driven by epidermal-derived interferon kappa. Ann. Rheum. Dis..

[82] Kuhn RM, Haussler D, Kent WJ (2013). The UCSC genome browser and associated tools. Brief. Bioinform.

[83] Dobin A (2013). STAR: ultrafast universal RNA-seq aligner. Bioinformatics.

[84] Karolchik D (2004). The UCSC Table Browser data retrieval tool. Nucleic Acids Res..

[85] Okonechnikov K, Conesa A, Garcia-Alcalde F (2016). Qualimap 2: advanced multi-sample quality control for high-throughput sequencing data. Bioinformatics.

[86] Adiconis X (2013). Comparative analysis of RNA sequencing methods for degraded or low-input samples. Nat. Methods.

[87] Li H (2009). The sequence alignment/map format and SAMtools. Bioinformatics.

[88] Robinson JT (2011). Integrative genomics viewer. Nat. Biotechnol..

[89] Liao Y, Smyth GK, Shi W (2013). The Subread aligner: fast, accurate and scalable read mapping by seed-and-vote. Nucleic Acids Res..

[90] Anders S, Huber W (2010). Differential expression analysis for sequence count data. Genome Biol..

[91] Love MI, Huber W, Anders S (2014). Moderated estimation of fold change and dispersion for RNA-seq data with DESeq2. Genome Biol..

[92] Kolde R, Laur S, Adler P, Vilo J (2012). Robust rank aggregation for gene list integration and meta-analysis. Bioinformatics.

[93] Yu G, Wang LG, Han Y, He QY (2012). clusterProfiler: an R package for comparing biological themes among gene clusters. OMICS.

[94] Yu G, He QY (2016). ReactomePA: an R/Bioconductor package for reactome pathway analysis and visualization. Mol. Biosyst..

[95] Kanehisa M, Goto S (2000). KEGG: kyoto encyclopedia of genes and genomes. Nucleic Acids Res..

[96] Luo W, Brouwer C (2013). Pathview: an R/Bioconductor package for pathway-based data integration and visualization. Bioinformatics.

[97] Kuhn A (2011). Photoprovocation in cutaneous lupus erythematosus: a multicenter study evaluating a standardized protocol. J. Invest Dermatol..

[98] Ruland V (2013). Updated analysis of standardized photoprovocation in patients with cutaneous lupus erythematosus. Arthritis Care Res..

