# Supplementary material for: Functionally impaired plasmacytoid dendritic cells and non-haematopoietic sources of type I interferon characterize human autoimmunity
Source: Nat Commun. 2020 Dec 1;11:6149. doi: 10.1038/s41467-020-19918-z (PMC7708979; doi:10.1038/s41467-020-19918-z)
Supplement: Supplementary file 1 — Supplementary Information [file 41467_2020_19918_MOESM1_ESM.pdf]

# SUPPLEMENTARY INFORMATION FILE

Functionally impaired plasmacytoid dendritic cells and non-haematopoietic sources of type I interferon dominate human autoimmunity

Antonios Psarras[1,2,3], Adewonuola Alase[1], Agne Antanaviciute[4], Ian M. Carr[4], Md Yuzaiful Md Yusof[1,2], Miriam Wittmann[1,2], Paul Emery[1,2], George C. Tsokos[3], Edward M. Vital[1,2]

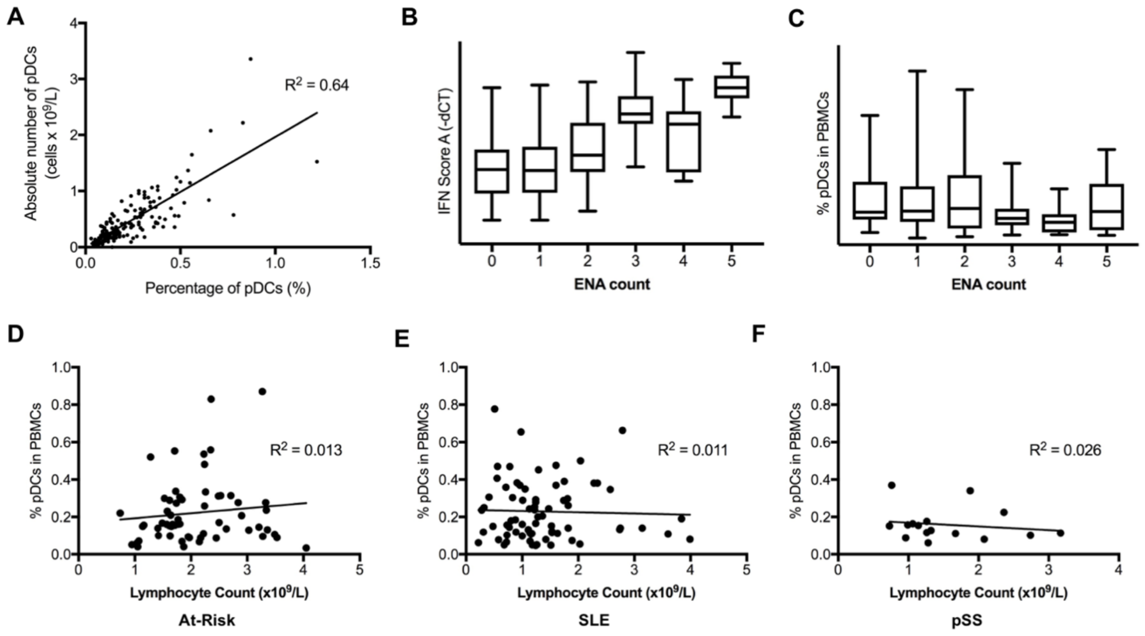


**Supplementary Figure 1.** Association of peripheral pDCs with other immunological parameters. (**A**) Percentage and absolute numbers of pDCs are closely correlated (results merged from healthy controls, At-Risk individuals, patients with SLE and pSS. (**B**) Higher IFN Score A was correlated to a higher ENA count. (**C**) The percentage of pDCs in peripheral blood showed no correlation with ENA count. (**D**) Association of percentage of pDCs in peripheral blood and lymphocyte count in At-Risk individuals. (**E**) Association of percentage of pDCs in peripheral blood and lymphocyte count in patients with SLE. (**F**) Association of percentage of pDCs in peripheral blood and lymphocyte count in patients with pSS. Data are represented as mean ± SEM. Nonlinear regression (**A-F**).

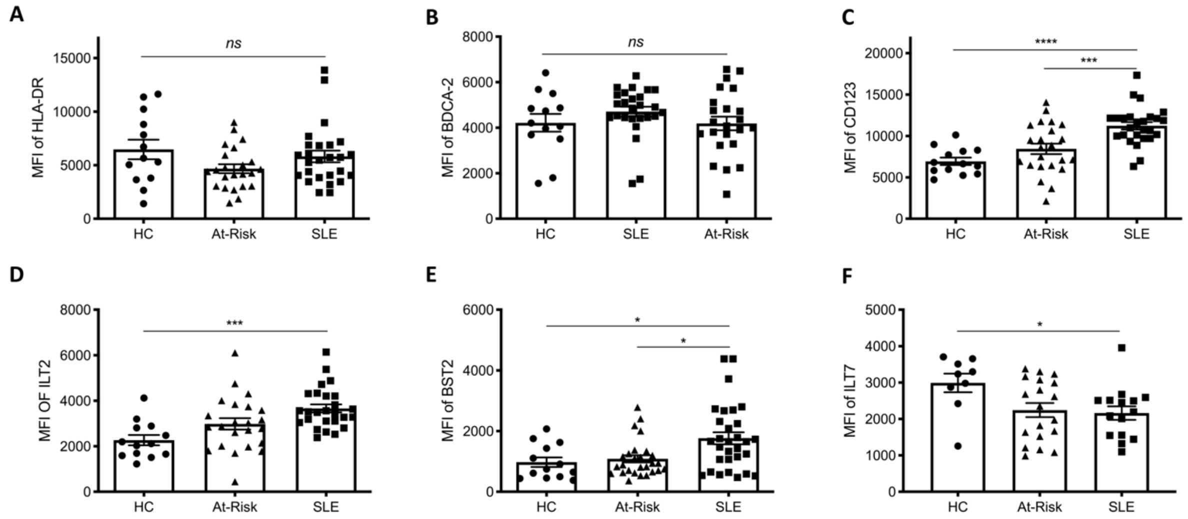


**Supplementary Figure 2.** Phenotyping peripheral pDCs in At-Risk individuals and patients with SLE in comparison with healthy controls (HC) for the expression of HLA-DR (**A**), BDCA-2 (**B**), CD123 (**C**), ILT2 (**D**), BST2 (**E**), and ILT7 (**F**). Data are represented as mean ± SEM. **P* < 0.05; ****P* <0.001. 2-way ANOVA (**A-F**).

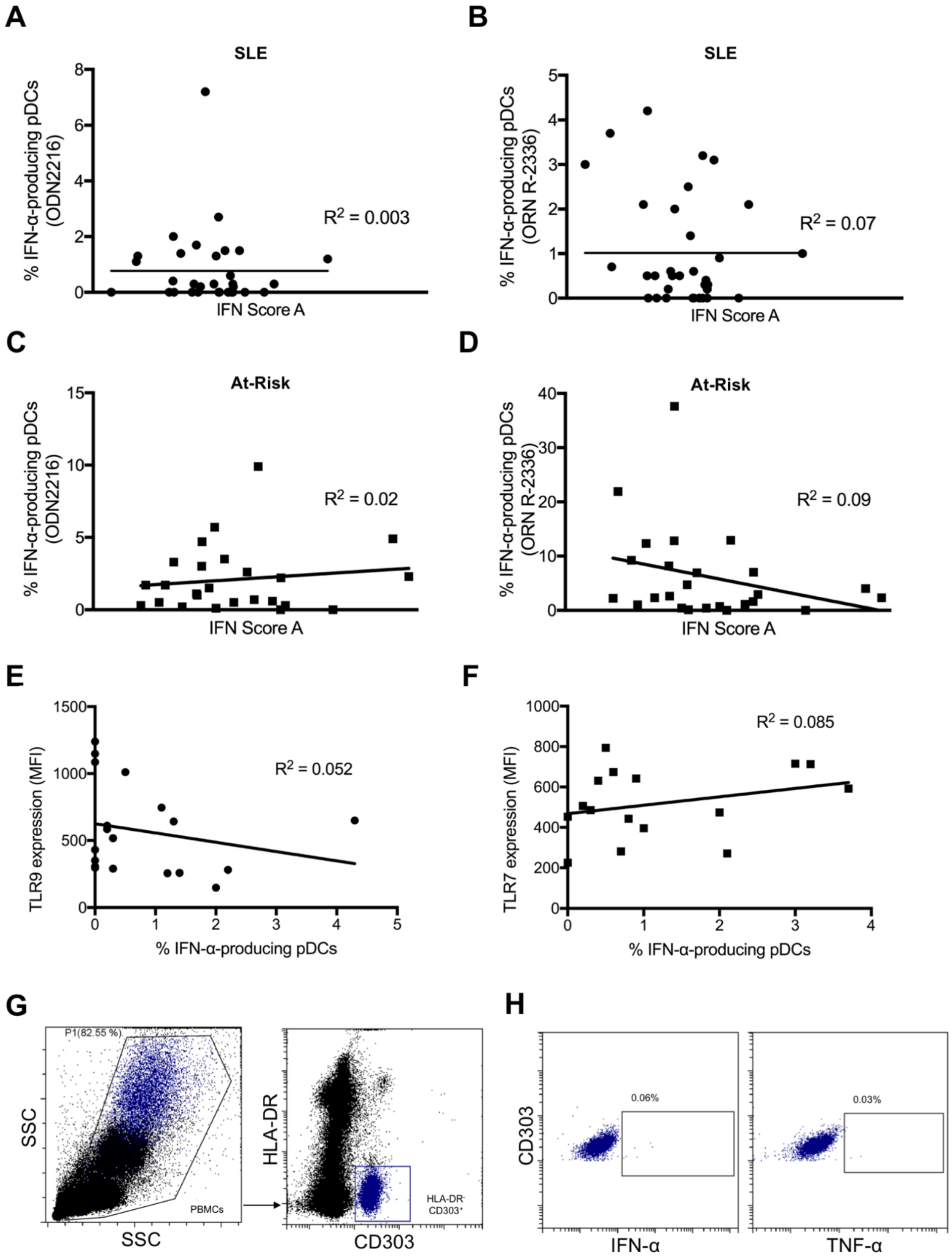


**Supplementary Figure 3.** (**A-D**) No association between TLR-mediated IFN-α production and IFN Score A in SLE patients and at-risk individuals. (**E**) Association of percentage of IFN-α production by pDCs and TLR9 expression in patients with SLE. (**F**) Association of percentage of IFN-α production by pDCs and TLR7 expression in patients with SLE. (**G**) Gating of HLA-DR$^-$CD303$^+$ cells from cultured PBMCs. (**H**) No production of IFN-α or TNF-α was detected by HLA-DR$^-$CD303$^+$ cells after TLR9 stimulation (ODN 2216). Data are represented as mean ± SEM. Nonlinear regression (**A-F**).

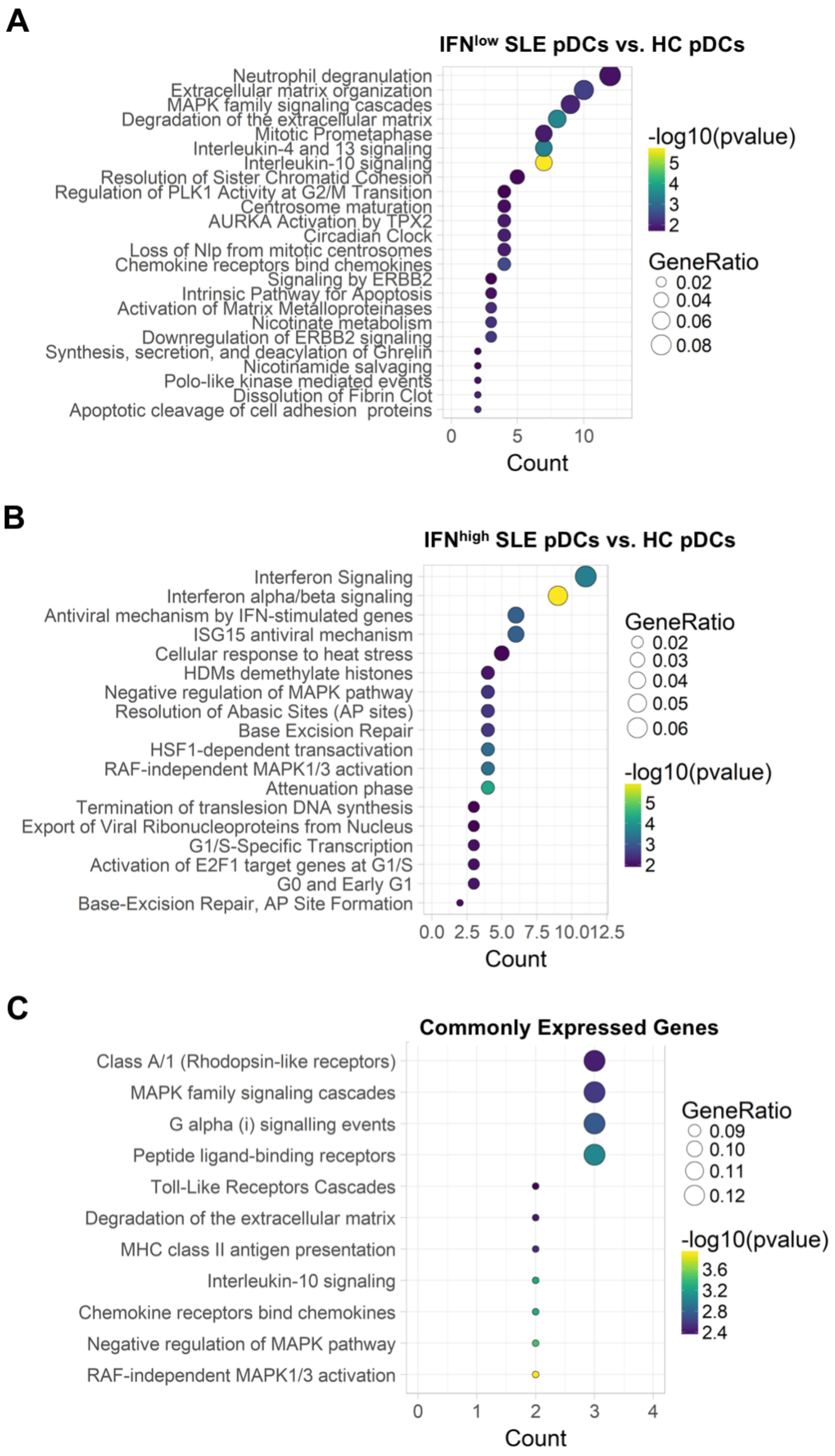


**Supplementary Figure 4.** Reactome Pathway Enrichment in differentially expressed genes in pDCs of IFN$^{low}$ SLE patients (**A**), IFN$^{high}$ SLE patients (**B**) and genes commonly expressed to both (**C**) in comparison with pDCs from healthy controls.

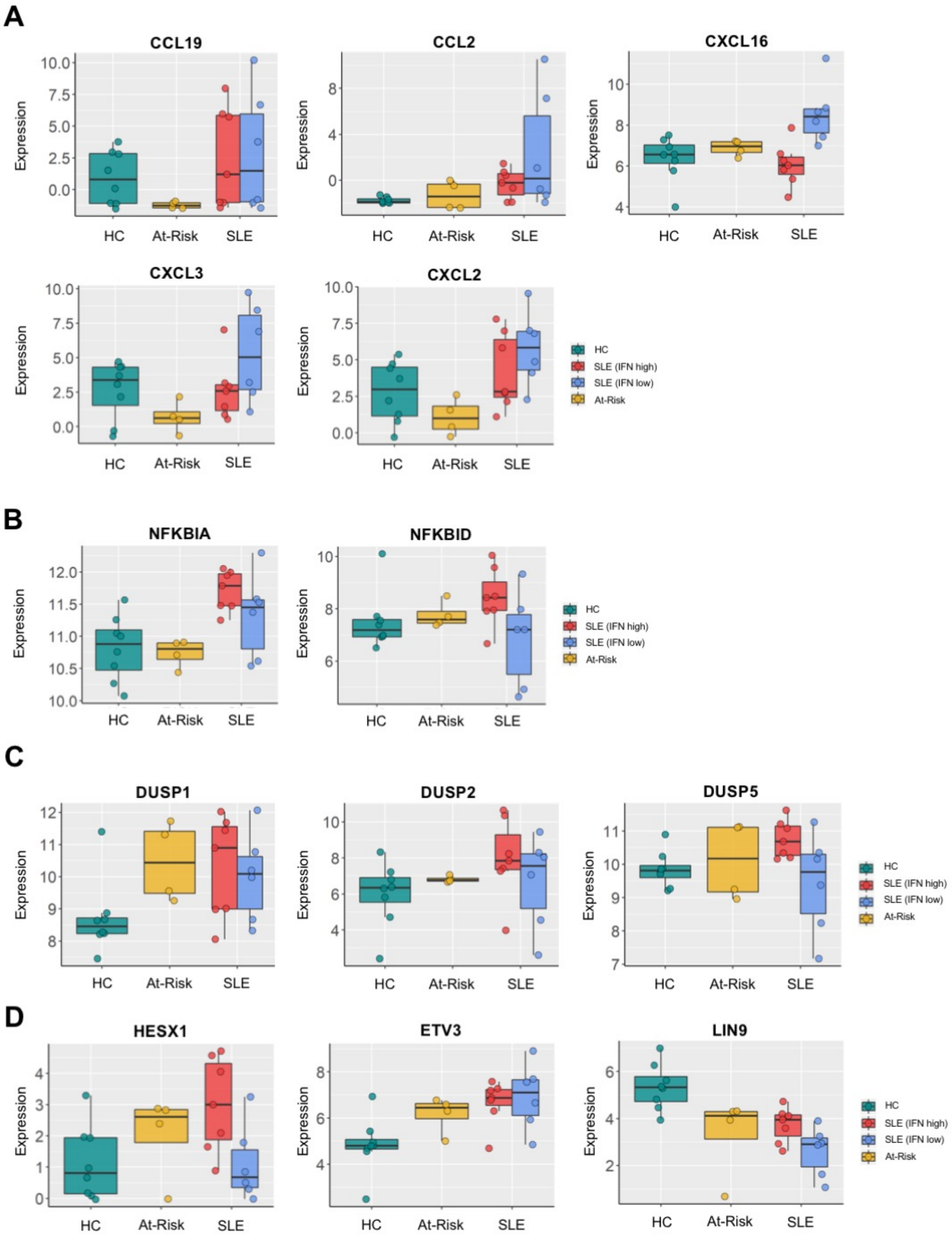


**Supplementary Figure 5.** Differentially expressed genes in pDCs of healthy controls (HC), at-risk individuals (At-Risk), IFN$^{low}$ SLE and IFN$^{high}$ SLE patients: (**A**) Chemokines; (**B**) NF-κB inhibitors; (**C**) Phosphatases; (**D**) Transcriptional repressors.

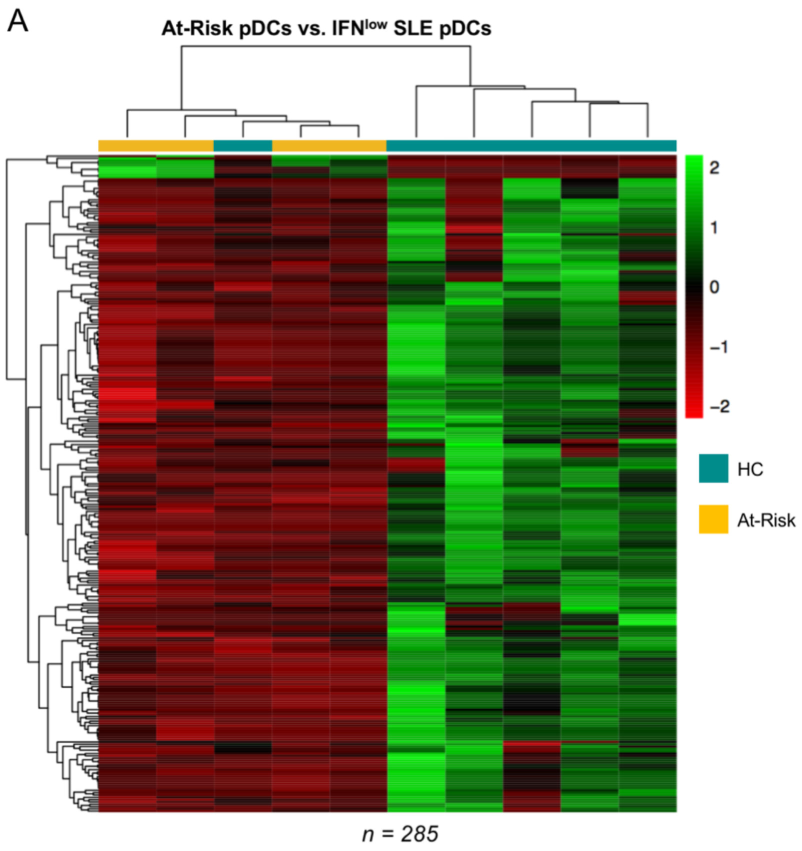


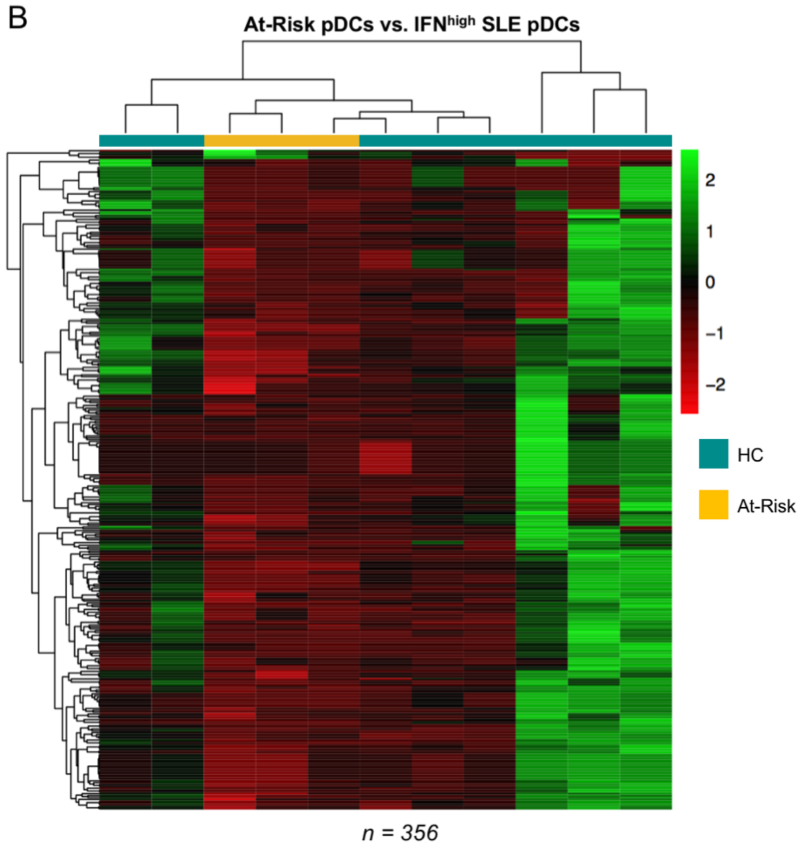


**Supplementary Figure 6.** Differentially expressed transcripts in At-Risk pDCs compared to IFN$^{low}$ SLE pDCs (**A**) and IFN$^{high}$ SLE pDCs (**B**).

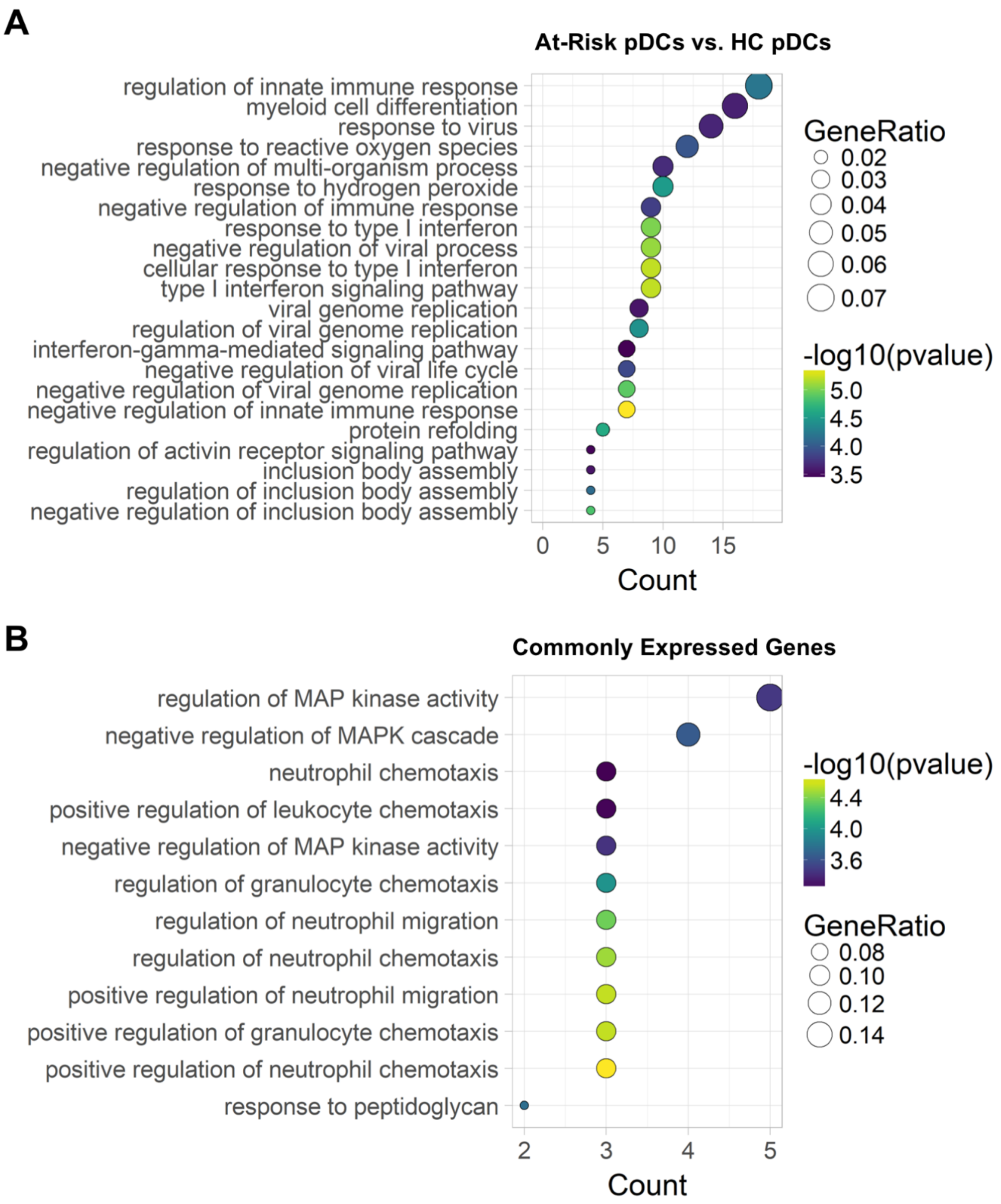


**Supplementary Figure 7.** Reactome Pathway Enrichment in differentially expressed genes in pDCs of At-Risk individuals in comparison with pDCs from healthy controls. (**A**), Enriched pathways for genes commonly expressed in pDCs of At-Risk individuals and SLE patients in comparison with pDCs from healthy controls.

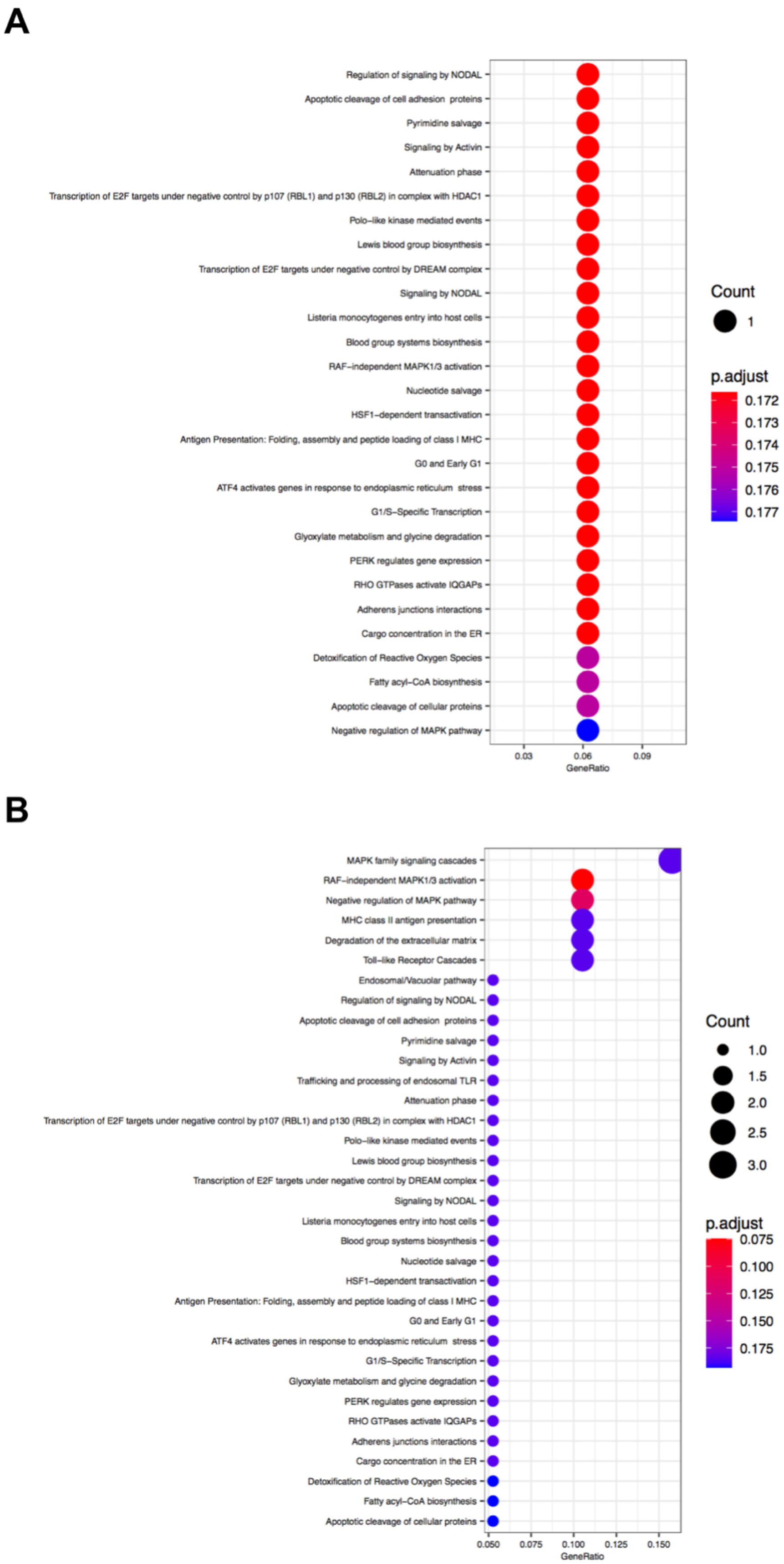


**Supplementary Figure 8.** Reactome Pathway Enrichment in the commonly differentially expressed genes in pDCs of IFN$^{low}$ SLE (**A**) and IFN$^{high}$ SLE patients (**B**) in comparison with pDCs from At-Risk individuals.

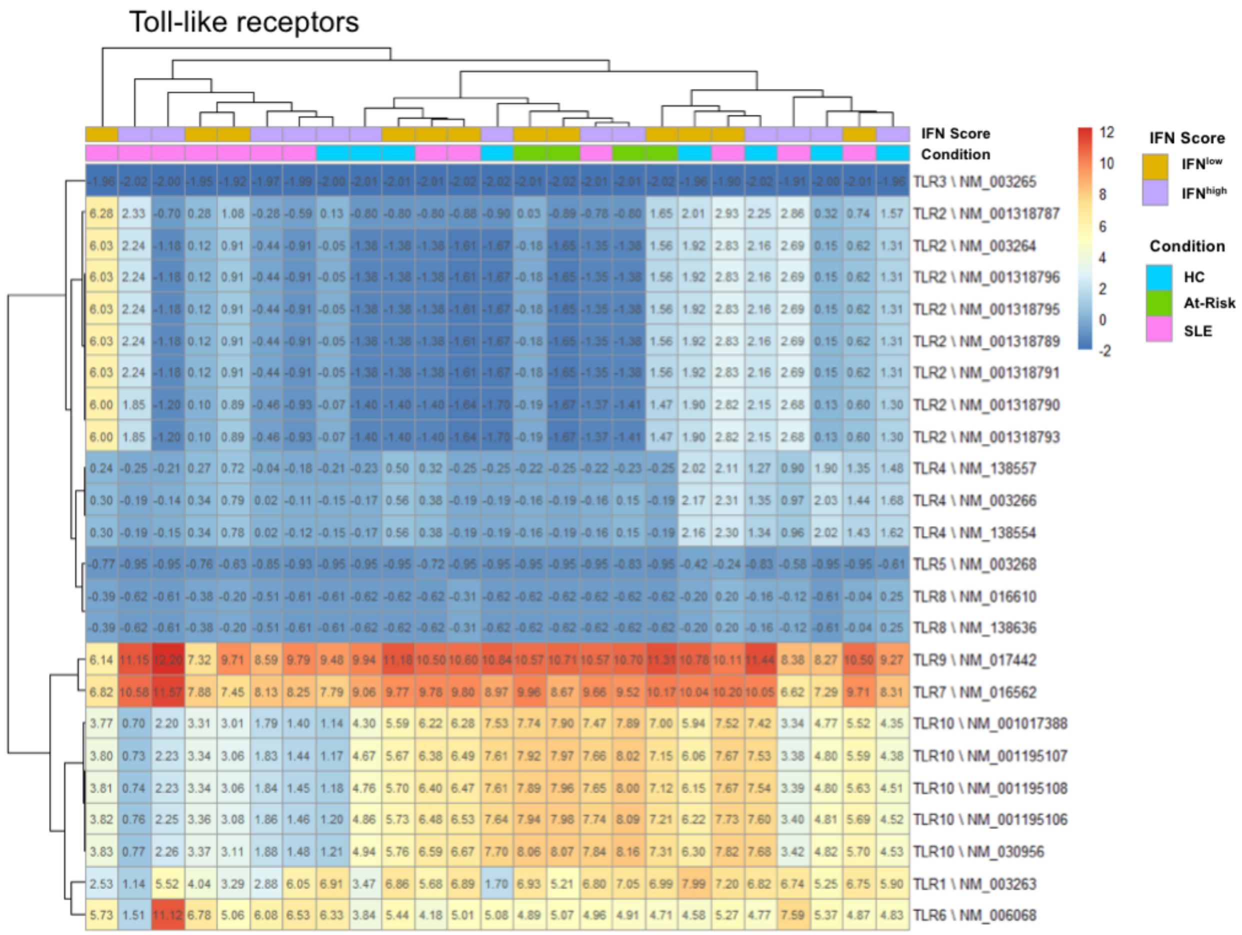


**Supplementary Figure 9.** Differential expression of Toll-like receptors (TLRs) in pDCs of healthy controls (HC), at-risk individuals (At-Risk), and SLE patients.

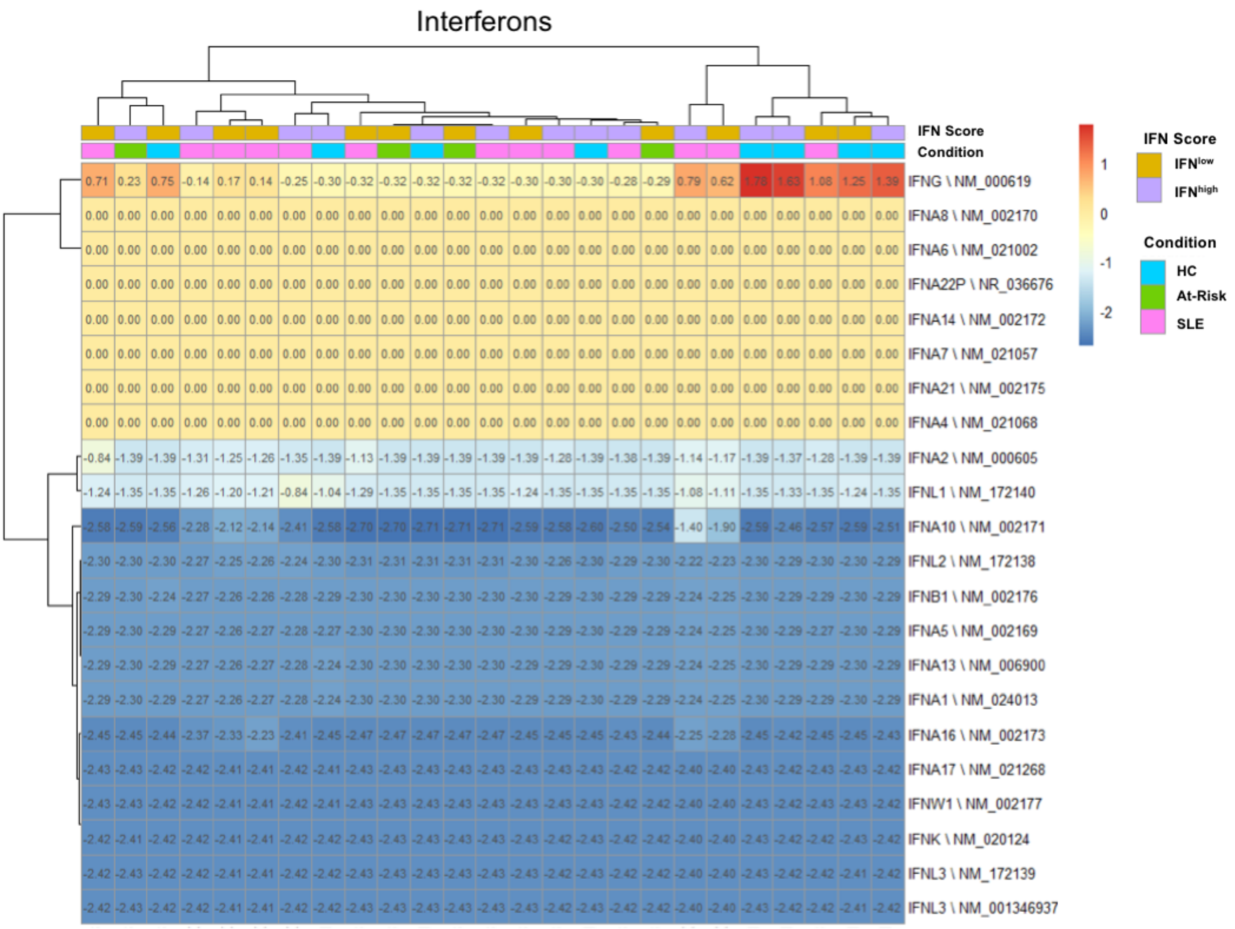


**Supplementary Figure 10.** Differential expression of type I, II, and III IFNs in pDCs of healthy controls (HC), At-risk individuals (At-Risk), and SLE patients.

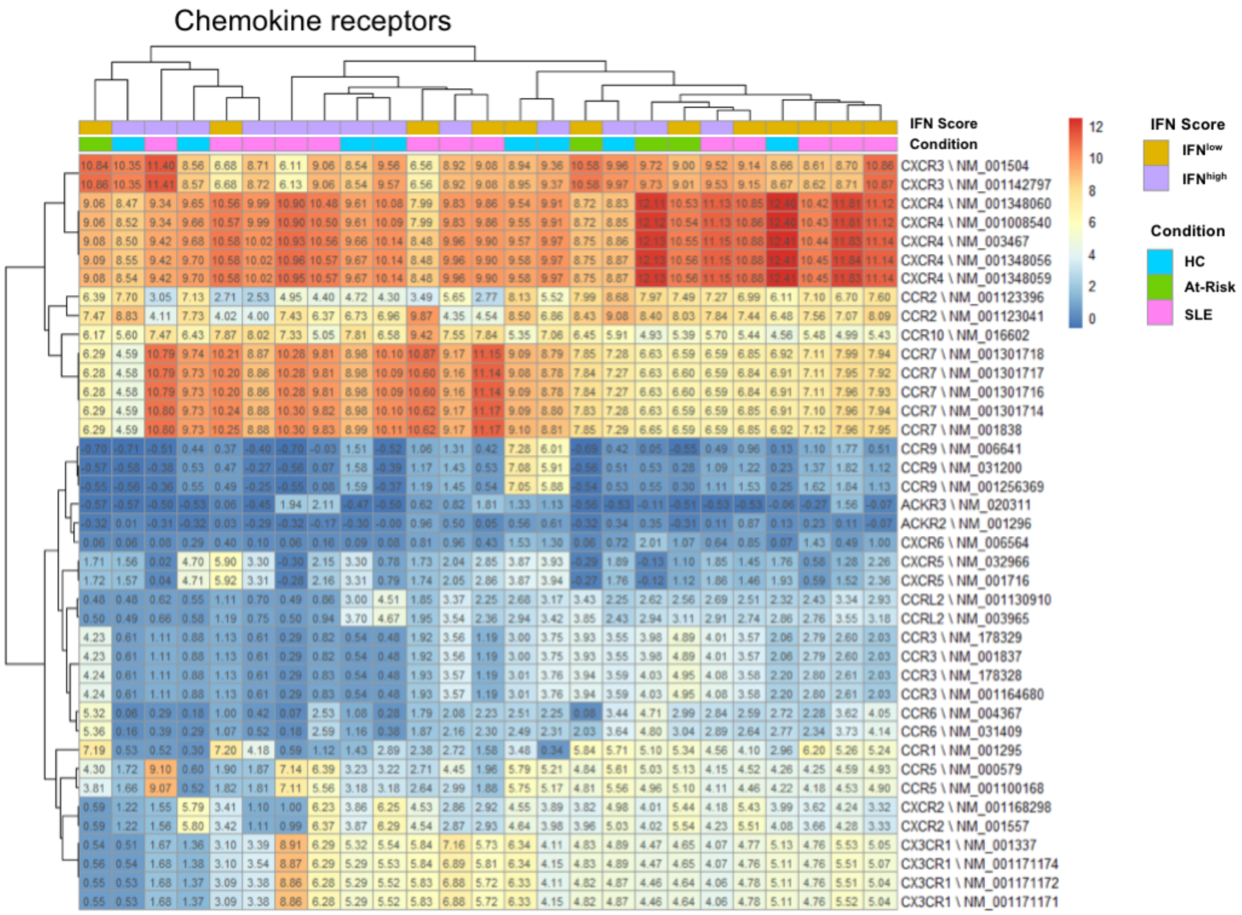


**Supplementary Figure 11.** Differential expression of chemokine receptors in pDCs of healthy controls (HC), At-risk individuals (At-Risk), and SLE patients.

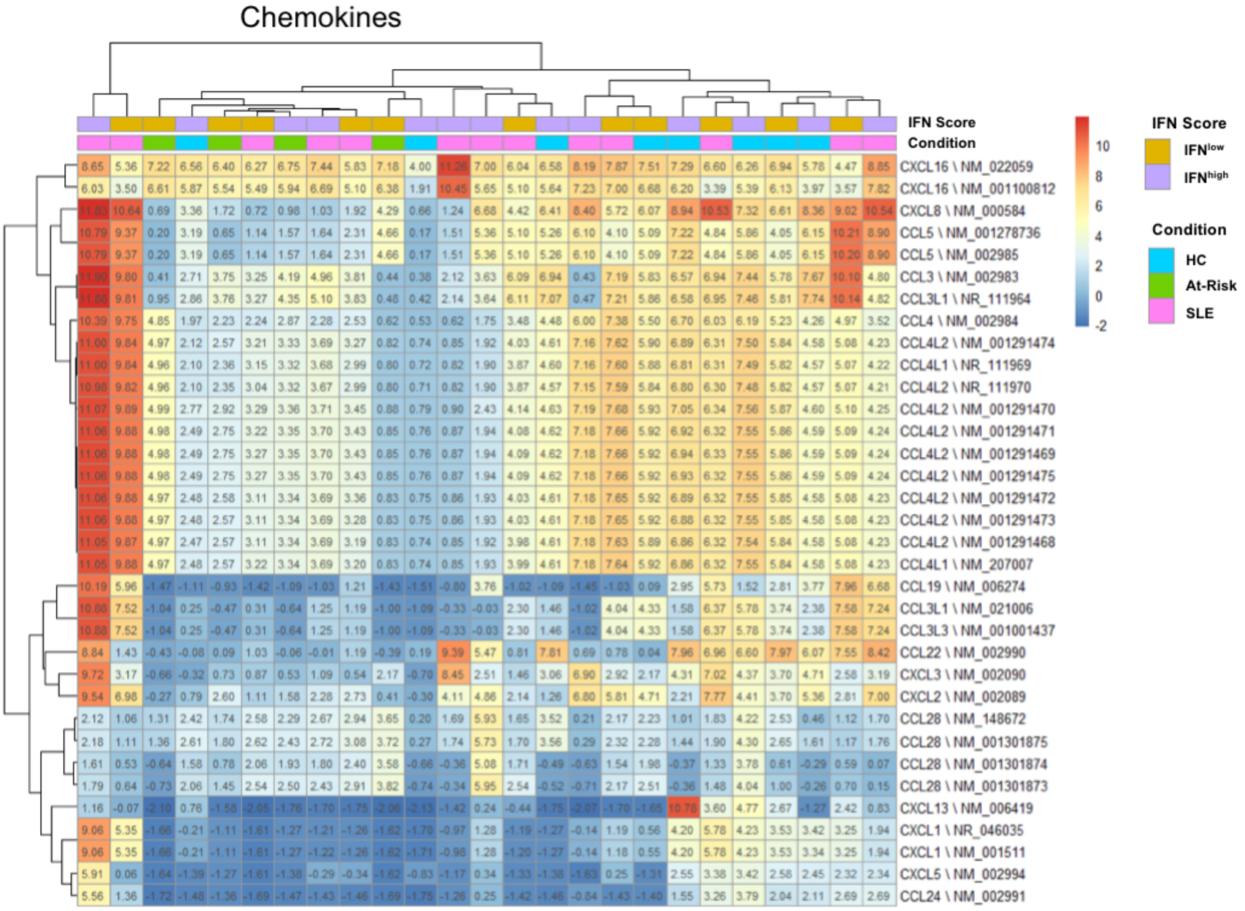


**Supplementary Figure 12.** Differential expression of chemokines in pDCs of healthy controls (HC), At-risk individuals (At-Risk), and SLE patients.

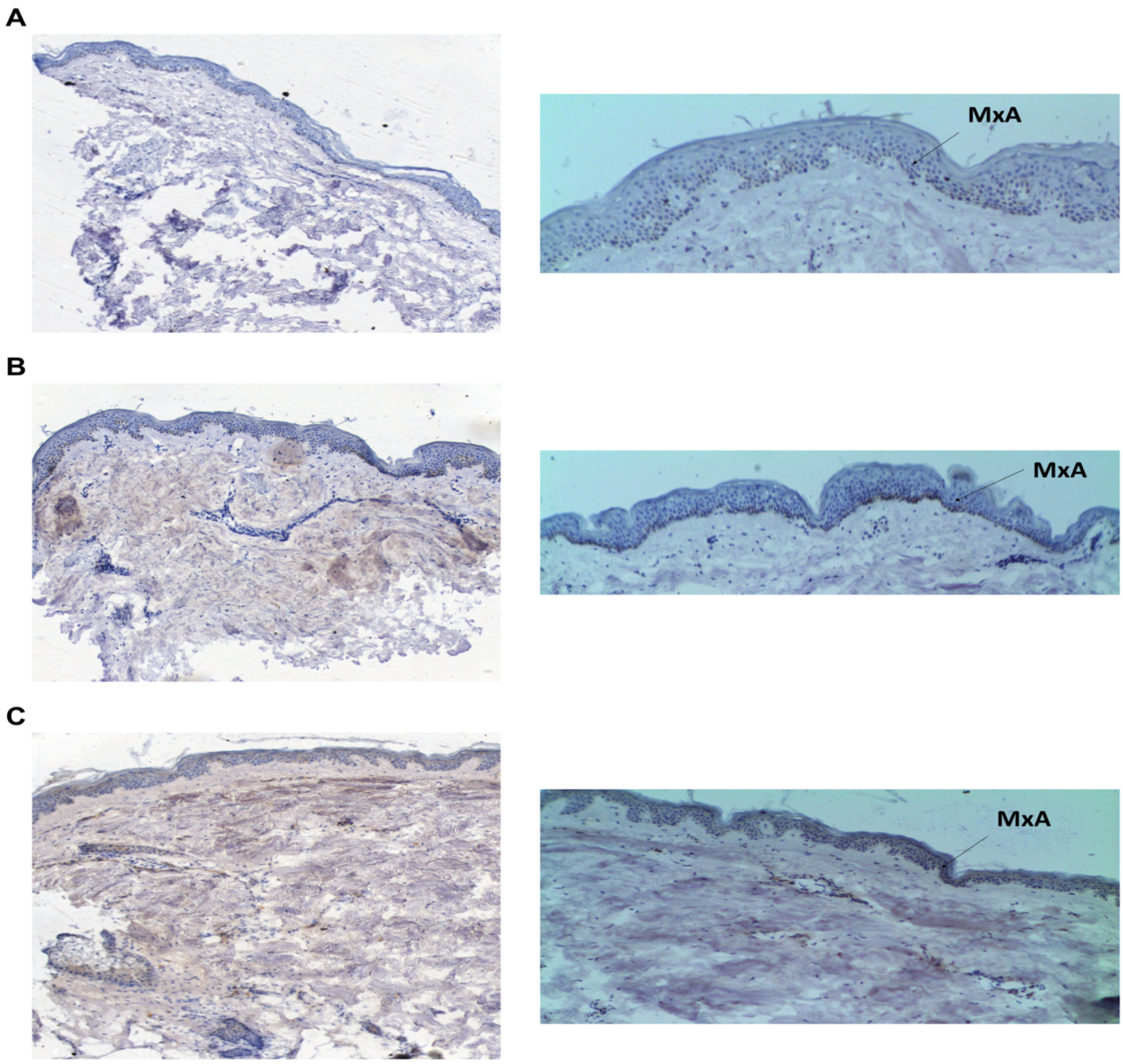


**Supplementary Figure 13.** Expression of interferon-induced GTP-binding protein MxA in the epidermis from skin biopsies of healthy controls (n = 3), skin biopsies of At-Risk individuals (n = 3), and non-lesional skin biopsies of SLE patients (n = 3) using immunohistochemistry (IHC). MxA positivity is indicated accordingly by the intensity of brown staining. Representative IHC images of: (**A**) healthy control, (**B**) At-Risk, and (**C**) SLE.

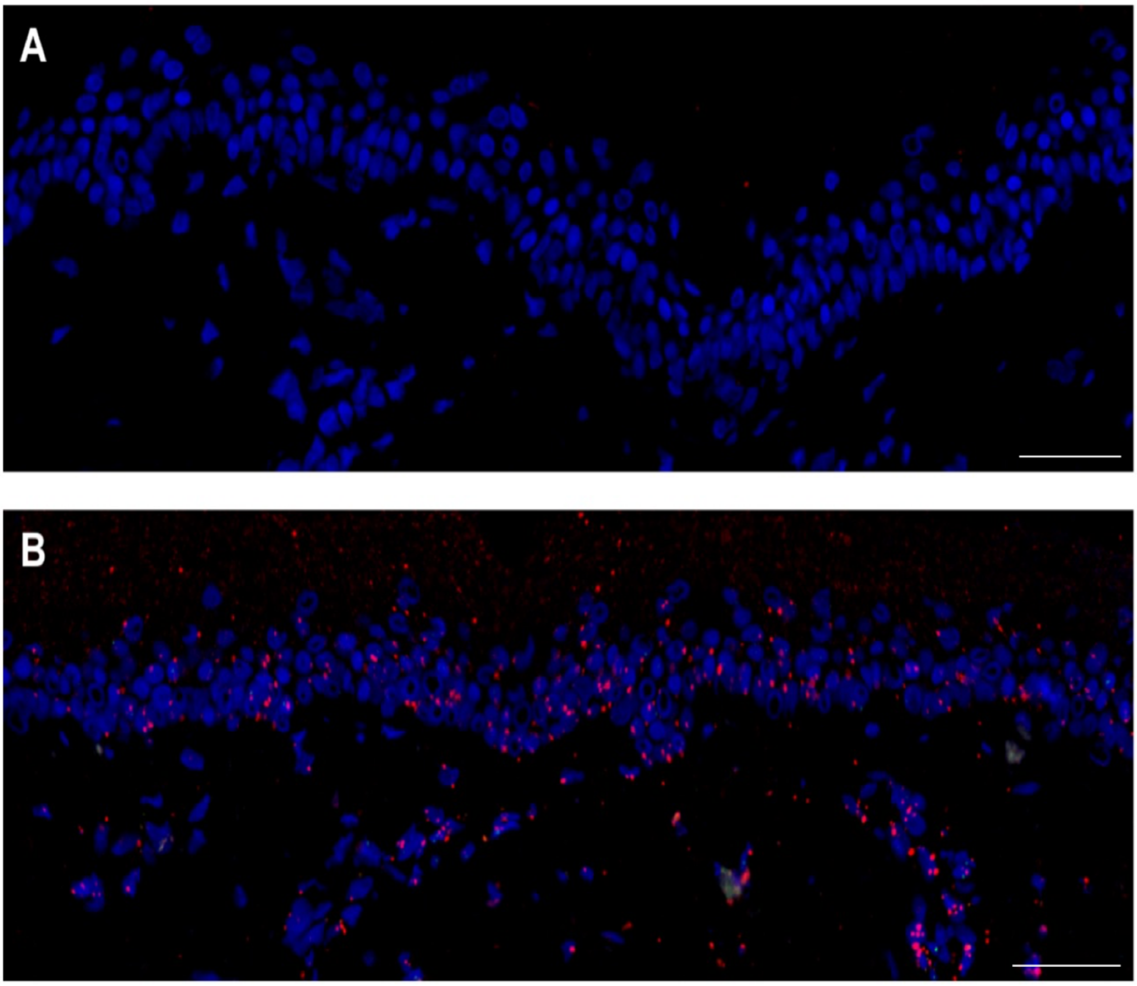


**Supplementary Figure 14.** Representative pictures of *in situ* hybridization using RNAscope Multiplex Fluorescent Reagent Kit v2 (Advanced Cell Diagnostics): (**A**) negative control; (**B**) positive control (proprietary probes provided by the manufacturer). Scale bars: 100 µm.

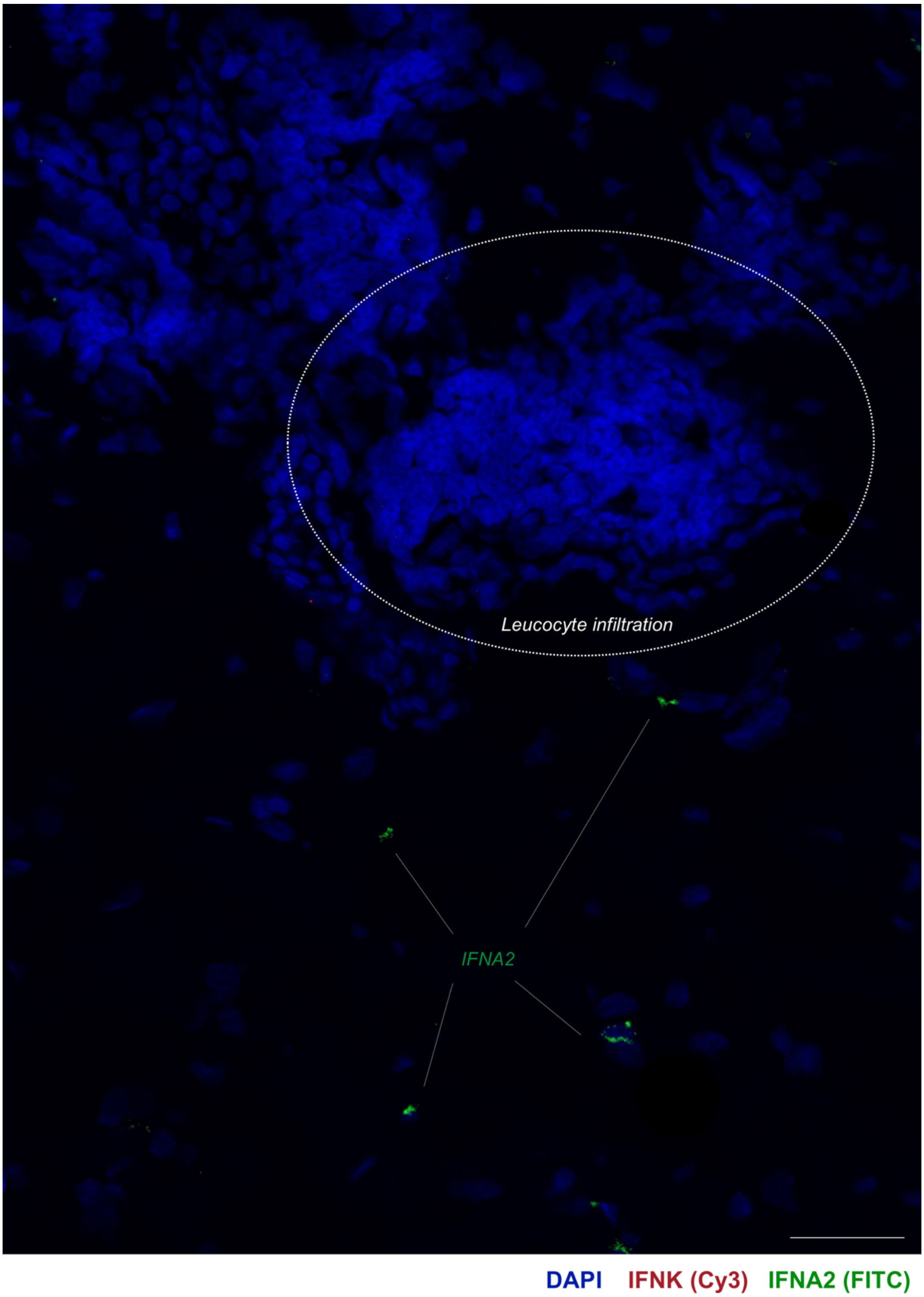


**Supplementary Figure 15.** Area of leucocyte infiltration and connective tissue of a patient with SLE with active skin lesion. Skin biopsies were hybridized using RNAscope *in situ* hybridization technology with custom-designed target probes for *IFNA2* and *IFNK*. Hybridization signals were amplified and detected using TSA Plus fluorescein (FITC) for IFNA2 and TSA Plus Cyanine 3 (Cy3) for *IFNK*. Nuclei were highlighted using DAPI. *IFNA2* expression was detected in cells within the connective tissue but not in infiltrating leucocytes. Scale bars: 100 µm.

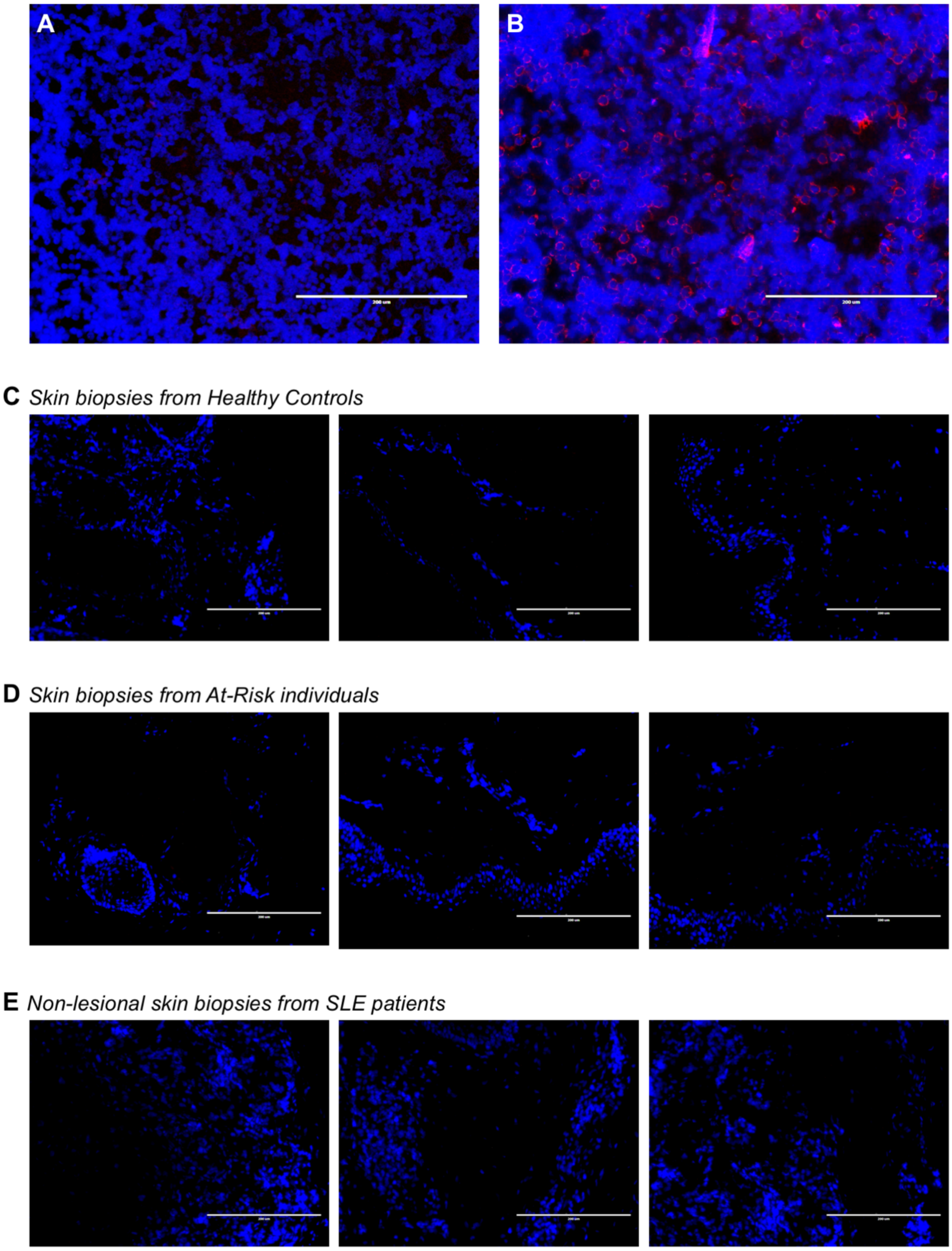


**Supplementary Figure 16.** Expression of BDCA-4 positive cells in skin biopsies from of healthy controls (n = 3), skin biopsies of At-Risk individuals (n = 3), and non-lesional skin biopsies of SLE patients (n = 3) using immunofluorescence. Isolated pDCs by negative selection were used as positive control: (**A**) unstained, (**B**) BDCA-4 stained. BDCA-4 positivity is indicated by the red fluorescence. No expression of BDCA-4 was identified in any of the skin biopsies acquired from healthy controls (**C**), At-Risk individuals (**D**), and SLE patients (**E**).

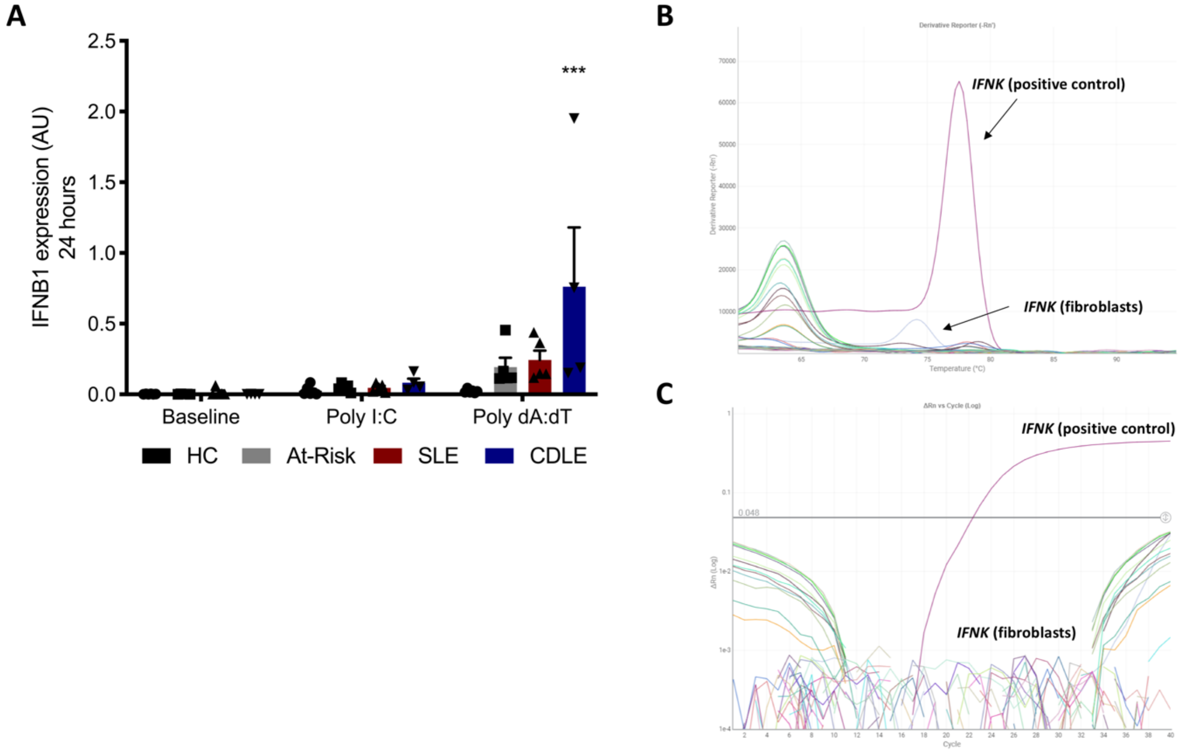


**Supplementary Figure 17.** Human dermal fibroblasts were isolated from fresh skin biopsies and were then cultured in the absence or presence of Poly I:C (1 µg/mL) or Poly dA:dT (100 ng/mL). (**A**) Expression level of *IFNB1* in dermal fibroblasts from healthy controls (HC), At-Risk individuals (At-Risk), SLE patients (SLE), and patients with cutaneous discoid lupus erythematosus (CDLE) after in vitro culture for 24 hours. (**B** and **C**) Raw qPCR data for IFNK expression of dermal fibroblasts from all samples; no expression detected. Data are represented as mean ± SEM. ***$P$ < 0.001. 2-way ANOVA (**A**).

| Age, median (range) years | 44 (18 -76) |
|---|---|
| Female patients (%) | 94 |
| Ethnicity (%) | |
| Caucasian | 74 |
| South Asian | 16 |
| East Asian | 5 |
| African/Caribbean | 5 |
| Clinical symptoms: BILAG Score A/B (%) | |
| Mucocutaneous | 23 |
| Musculoskeletal | 23 |
| Haematological | 3 |
| Renal | 16 |
| Neurological | 3 |
| Cardiorespiratory | 2 |
| Gastrointestinal | 3 |
| Opthalmic | 0 |
| General | 0 |
| Hydroxychloroquine (%) | 83 |
| Other immunosuppressants (%) | |
| Methotrexate | 14 |
| Azathioprine | 18 |
| Mycophenolate mofetil | 27 |
| Cyclophosphamide | 1 |
| Oral steroids (%) | 47 |

**Supplementary Table 1.** Clinical characteristics and treatment of SLE patients.

| Gene | IFN$^{low}$ SLE pDCs vs. HC pDCs Fold Change (log2) | *P* value | FDR |
|---|---|---|---|
| *ULBP2* | 19.757 | < 0.001 | < 0.001 |
| *C10orf35* | 18.302 | < 0.001 | < 0.001 |
| *TPSB2* | 18.228 | < 0.001 | < 0.001 |
| *TMEM216* | -4.981 | < 0.001 | < 0.001 |
| *CXCL2* | 6.379 | < 0.001 | < 0.001 |
| *PLA2G7* | 9.712 | < 0.001 | < 0.001 |
| *DNAJB4* | 1.965 | < 0.001 | 0.001 |
| *ZBP1* | -7.254 | < 0.001 | 0.001 |
| *LUCAT1* | 6.207 | < 0.001 | 0.006 |
| *LOC100861532* | 2.839 | < 0.001 | 0.007 |
| *LGALSL* | 6.161 | < 0.001 | 0.007 |
| *SNORD95* | 4.390 | < 0.001 | 0.007 |
| *LOC100008587* | 3.428 | < 0.001 | 0.007 |
| *BBC3* | 2.962 | < 0.001 | 0.011 |
| *SPR* | 6.792 | < 0.001 | 0.015 |
| *FN1* | 4.352 | < 0.001 | 0.015 |
| *PHF3* | 1.125 | < 0.001 | 0.015 |
| *HSPB1* | 1.527 | < 0.001 | 0.015 |
| *CLEC5A* | 8.287 | < 0.001 | 0.015 |
| *CKAP4* | -6.563 | < 0.001 | 0.015 |
| *IRAIN* | 5.493 | < 0.001 | 0.015 |
| *FAM157C* | 3.945 | < 0.001 | 0.015 |
| *DNAJB1* | 2.108 | < 0.001 | 0.015 |
| *ETV3* | 3.037 | < 0.001 | 0.017 |
| *TPSAB1* | 9.650 | < 0.001 | 0.020 |
| *SLC6A6* | 3.758 | < 0.001 | 0.020 |
| *NPAS1* | 5.880 | < 0.001 | 0.020 |
| *GADD45B* | 2.245 | < 0.001 | 0.021 |
| *IFITM1* | -3.619 | < 0.001 | 0.022 |
| *COCH* | -7.844 | < 0.001 | 0.022 |
| *ANO8* | 3.516 | < 0.001 | 0.022 |
| *LINC00623* | 3.718 | < 0.001 | 0.022 |
| *HSPA4* | -2.406 | < 0.001 | 0.023 |
| *AOAH* | -6.704 | < 0.001 | 0.023 |
| *HOXB3* | 4.793 | < 0.001 | 0.023 |
| *SLC8A2* | 5.243 | < 0.001 | 0.023 |
| *NPL* | 4.663 | < 0.001 | 0.025 |
| *ZNF678* | 4.063 | < 0.001 | 0.025 |
| *CNST* | 2.130 | < 0.001 | 0.025 |
| *RNY5* | 3.224 | < 0.001 | 0.025 |
| *PLEKHA8P1* | -4.947 | < 0.001 | 0.025 |
| *DUSP1* | 2.903 | < 0.001 | 0.027 |
| *LOC100008589* | 2.695 | < 0.001 | 0.028 |
| *NRP2* | 6.969 | < 0.001 | 0.028 |
| *EGR2* | 7.175 | < 0.001 | 0.029 |
| *FCGR2B* | -6.697 | < 0.001 | 0.033 |
| *NRG2* | 5.224 | < 0.001 | 0.033 |
| *LOC221946* | 5.139 | < 0.001 | 0.033 |
| *PLCXD1* | 3.230 | < 0.001 | 0.033 |
| *LACC1* | 4.975 | < 0.001 | 0.033 |

**Supplementary Table 2.** Top 50 genes that are differentially expressed in pDCs of IFN$^{low}$ SLE patients in comparison with pDCs from healthy controls (HC).

| | IFN$^{high}$ SLE pDCs vs. HC pDCs | | |
|---|---|---|---|
| **Gene** | **Fold Change (log2)** | ***P* value** | **FDR** |
| *IFI44* | 5.403 | < 0.001 | < 0.001 |
| *CAMP* | 7.261 | < 0.001 | < 0.001 |
| *OASL* | 6.812 | < 0.001 | < 0.001 |
| *SLC8A2* | 5.644 | < 0.001 | < 0.001 |
| *ATF3* | 5.870 | < 0.001 | < 0.001 |
| *CMPK2* | 7.710 | < 0.001 | < 0.001 |
| *BCL2* | 5.485 | < 0.001 | < 0.001 |
| *LGALSL* | 6.240 | < 0.001 | < 0.001 |
| *ORM1* | 8.011 | < 0.001 | < 0.001 |
| *NCMAP* | 4.931 | < 0.001 | < 0.001 |
| *HCG11* | -3.108 | < 0.001 | < 0.001 |
| *PARP14* | 3.402 | < 0.001 | < 0.001 |
| *MSL2* | 2.238 | < 0.001 | 0.001 |
| *DDX60L* | 6.470 | < 0.001 | 0.001 |
| *PPDPF* | 1.798 | < 0.001 | 0.001 |
| *IER2* | 2.552 | < 0.001 | 0.001 |
| *NR4A1* | 5.230 | < 0.001 | 0.002 |
| *TFB1M* | -2.213 | < 0.001 | 0.002 |
| *GFOD1* | 4.279 | < 0.001 | 0.002 |
| *TP53INP2* | 5.236 | < 0.001 | 0.002 |
| *TMEM177* | -3.452 | < 0.001 | 0.002 |
| *LIPT1* | -2.499 | < 0.001 | 0.002 |
| *JUND* | 2.486 | < 0.001 | 0.002 |
| *THG1L* | -2.089 | < 0.001 | 0.002 |
| *CCDC121* | 6.073 | < 0.001 | 0.002 |
| *SNORD14C* | 4.151 | < 0.001 | 0.002 |
| *C2orf74* | -2.833 | < 0.001 | 0.002 |
| *CISD1* | -2.392 | < 0.001 | 0.002 |
| *HIST1H4F* | -2.531 | < 0.001 | 0.002 |
| *SEMA7A* | 3.150 | < 0.001 | 0.002 |
| *ETV3L* | 3.865 | < 0.001 | 0.002 |
| *CEACAM1* | 5.448 | < 0.001 | 0.002 |
| *L3MBTL2* | -1.887 | < 0.001 | 0.002 |
| *ETV3* | 2.959 | < 0.001 | 0.002 |
| *NPAS1* | 5.469 | < 0.001 | 0.002 |
| *QRICH2* | 4.336 | < 0.001 | 0.002 |
| *LTC4S* | 5.089 | < 0.001 | 0.003 |
| *ARID5A* | 1.781 | < 0.001 | 0.003 |
| *EIF2AK2* | 2.358 | < 0.001 | 0.003 |
| *HES4* | 4.895 | < 0.001 | 0.003 |
| *LINC00847* | -2.487 | < 0.001 | 0.003 |
| *NSUN7* | 5.334 | < 0.001 | 0.003 |
| *DNAJB1* | 4.229 | < 0.001 | 0.003 |
| *KLF8* | -4.556 | < 0.001 | 0.003 |
| *RSAD2* | 4.959 | < 0.001 | 0.003 |
| *JUN* | 2.582 | < 0.001 | 0.003 |
| *MON1A* | -2.248 | < 0.001 | 0.003 |
| *IFI44L* | 3.195 | < 0.001 | 0.004 |
| *WDR87* | 6.155 | < 0.001 | 0.004 |
| *IRAIN* | 4.774 | < 0.001 | 0.004 |

**Supplementary Table 3.** Top 50 genes that are differentially expressed in pDCs of IFN$^{high}$ SLE patients in comparison with pDCs from healthy controls (HC).

| Gene | IFN$^{low}$ SLE pDCs vs. HC pDCs | | | IFN$^{high}$ SLE pDCs vs. HC pDCs | | |
|---|---|---|---|---|---|---|
| | Fold Change (log2) | *P* value | FDR | Fold Change (log2) | *P* value | FDR |
| *ETV3* | 3.037 | < 0.001 | 0.017 | 2.959 | < 0.001 | 0.002 |
| *ATF3* | 3.914 | < 0.001 | 0.052 | 5.209 | < 0.001 | 0.000 |
| *LIN9* | -3.716 | < 0.001 | 0.058 | -2.423 | < 0.001 | 0.031 |
| *LGALSL* | 6.161 | < 0.001 | 0.007 | 6.240 | < 0.001 | < 0.001 |
| *ZNF2* | -4.108 | < 0.001 | 0.058 | -2.907 | < 0.001 | 0.020 |
| *LIPT1* | -3.224 | < 0.001 | 0.050 | -2.499 | < 0.001 | 0.002 |
| *SEC24D* | -2.559 | < 0.001 | 0.051 | -1.605 | 0.001 | 0.038 |
| *CXCL2* | 6.379 | < 0.001 | < 0.001 | 4.163 | < 0.001 | 0.019 |
| *CBR4* | -3.260 | < 0.001 | 0.052 | -1.546 | < 0.001 | 0.011 |
| *DUSP1* | 2.903 | < 0.001 | 0.027 | 2.386 | < 0.001 | 0.021 |
| *LTC4S* | 5.509 | < 0.001 | 0.033 | 5.089 | < 0.001 | 0.003 |
| *SNORD95* | 4.390 | < 0.001 | 0.007 | 3.106 | < 0.001 | 0.016 |
| *PHF3* | 1.125 | < 0.001 | 0.015 | 1.531 | < 0.001 | 0.028 |
| *LOC441242* | -2.798 | 0.001 | 0.068 | -2.494 | < 0.001 | 0.015 |
| *FUT10* | -3.275 | 0.002 | 0.100 | -2.388 | < 0.001 | 0.016 |
| *LOC102724580* | 3.699 | 0.001 | 0.088 | 4.080 | < 0.001 | 0.004 |
| *CCL19* | 4.468 | < 0.001 | 0.057 | 3.828 | < 0.001 | 0.020 |
| *CTSL* | 6.460 | < 0.001 | 0.040 | 4.114 | < 0.001 | 0.020 |
| *PUDP* | -2.632 | < 0.001 | 0.058 | -2.752 | < 0.001 | 0.008 |
| *ATP7A* | -4.215 | < 0.001 | 0.035 | -2.853 | < 0.001 | 0.020 |
| *SAT1* | 1.642 | < 0.001 | 0.036 | 1.805 | < 0.001 | 0.009 |
| *MIR6087* | 2.376 | < 0.001 | 0.050 | 2.411 | < 0.001 | 0.028 |
| *ZFP91* | 2.619 | < 0.001 | 0.046 | 2.147 | 0.001 | 0.043 |
| *PAAF1* | -3.453 | 0.001 | 0.066 | -1.973 | < 0.001 | 0.033 |
| *DUSP8* | 6.050 | 0.001 | 0.085 | 6.392 | < 0.001 | 0.006 |
| *IRAK3* | 6.179 | < 0.001 | 0.045 | 4.152 | < 0.001 | 0.007 |
| *PLEKHA8P1* | -4.947 | < 0.001 | 0.025 | -2.810 | < 0.001 | 0.009 |
| *ACVR1B* | 5.196 | 0.001 | 0.059 | 4.169 | < 0.001 | 0.017 |
| *RN7SL1* | 2.867 | < 0.001 | 0.049 | 2.648 | < 0.001 | 0.034 |
| *RN7SL2* | 3.107 | < 0.001 | 0.033 | 2.806 | < 0.001 | 0.021 |
| *ATG14* | 2.520 | < 0.001 | 0.058 | 1.640 | < 0.001 | 0.028 |
| *REREP3* | 3.757 | < 0.001 | 0.046 | 3.672 | < 0.001 | 0.019 |
| *IRAIN* | 5.493 | < 0.001 | 0.015 | 4.774 | < 0.001 | 0.004 |
| *CDH1* | 3.795 | 0.001 | 0.061 | 2.895 | < 0.001 | 0.028 |
| *SCARNA21* | 2.549 | 0.001 | 0.074 | 2.980 | 0.001 | 0.041 |
| *C5AR1* | 6.724 | 0.002 | 0.098 | 5.210 | < 0.001 | 0.031 |
| *SLC8A2* | 5.243 | < 0.001 | 0.023 | 5.644 | < 0.001 | < 0.001 |
| *IER2* | 2.416 | < 0.001 | 0.050 | 2.552 | < 0.001 | 0.001 |
| *DNAJB1* | 2.108 | < 0.001 | 0.015 | 4.097 | < 0.001 | 0.005 |
| *NPAS1* | 5.880 | < 0.001 | 0.020 | 5.469 | < 0.001 | 0.002 |
| *LOC100008589* | 2.695 | < 0.001 | 0.028 | 2.969 | < 0.001 | 0.006 |
| *MIR3687-1* | 3.321 | 0.002 | 0.098 | 3.448 | < 0.001 | 0.007 |
| *MIR3687-2* | 3.321 | 0.002 | 0.098 | 3.448 | < 0.001 | 0.007 |
| *LOC100861532* | 2.839 | < 0.001 | 0.007 | 2.930 | < 0.001 | 0.005 |
| *WRB* | -2.306 | 0.001 | 0.068 | -2.027 | < 0.001 | 0.008 |
| **pDC-specific transcription factors** | | | | | | |
| *E2-2 (TCF4)* | 0.691 | 0.118 | 0.537 | 0.313 | 0.578 | 0.852 |
| *SPIB* | 0.657 | 0.279 | 0.719 | 0.235 | 0.744 | 0.922 |

**Supplementary Table 4.** Genes that are differentially expressed in pDCs of both IFN$^{low}$ and IFN$^{high}$ SLE patients in comparison with pDCs from healthy controls (HC).

| | At-Risk pDCs vs. HC pDCs | | |
|---|---|---|---|
| **Gene** | **Fold Change (log2)** | ***P* value** | **FDR** |
| *DNAJB4* | -1.938 | < 0.001 | 0.008 |
| *ATF3* | -2.990 | < 0.001 | 0.008 |
| *RNU6-1* | 2.960 | < 0.001 | 0.004 |
| *RNU6-9* | 2.957 | < 0.001 | 0.004 |
| *RNU6-7* | 2.960 | < 0.001 | 0.004 |
| *RNU6-8* | 2.960 | < 0.001 | 0.004 |
| *RNU6-2* | 2.957 | < 0.001 | 0.004 |
| *GSTM1* | 1.941 | < 0.001 | 0.005 |
| *GLRX2* | 2.736 | < 0.001 | 0.005 |
| *MIR4435-2HG* | 2.529 | < 0.001 | 0.006 |
| *STAT1* | -2.113 | < 0.001 | 0.004 |
| *COL4A4* | 2.891 | < 0.001 | 0.009 |
| *CYTOR* | 3.023 | < 0.001 | 0.002 |
| *ANKRD36BP2* | 3.771 | < 0.001 | < 0.001 |
| *CD38* | 2.778 | < 0.001 | 0.007 |
| *TXNDC15* | 2.401 | < 0.001 | 0.003 |
| *DUSP1* | -2.774 | < 0.001 | < 0.001 |
| *HIST1H2BB* | 3.194 | < 0.001 | < 0.001 |
| *HIST1H3A* | 2.916 | < 0.001 | 0.002 |
| *BET1* | 2.043 | < 0.001 | 0.010 |
| *SNORA109* | 3.523 | < 0.001 | 0.001 |
| *MECP2* | 1.951 | < 0.001 | 0.006 |
| *EIF1AY* | -3.234 | < 0.001 | 0.006 |
| *DDX3Y* | -4.419 | < 0.001 | < 0.001 |
| *KDM5D* | -3.804 | < 0.001 | < 0.001 |
| *CSKMT* | 3.363 | < 0.001 | < 0.001 |
| *EHD1* | 1.389 | < 0.001 | 0.010 |
| *RAB30* | 2.610 | < 0.001 | 0.005 |
| *ERBB3* | 3.265 | < 0.001 | 0.004 |
| *FKBP11* | 3.086 | < 0.001 | 0.002 |
| *CNPY2* | 2.266 | < 0.001 | 0.001 |
| *CKAP4* | 3.181 | < 0.001 | 0.006 |
| *C12orf65* | 2.252 | < 0.001 | 0.009 |
| *MRPS31* | 2.589 | < 0.001 | < 0.001 |
| *RPPH1* | 2.977 | < 0.001 | 0.001 |
| *FOS* | -3.826 | < 0.001 | 0.000 |
| *CRIP2* | 3.717 | < 0.001 | 0.000 |
| *SLCO3A1* | 3.129 | < 0.001 | 0.008 |
| *IGHV1OR15-1* | 3.725 | < 0.001 | < 0.001 |
| *LOC102724760* | 3.725 | < 0.001 | < 0.001 |
| *TNFRSF17* | 3.643 | < 0.001 | < 0.001 |
| *NPIPB8* | 3.034 | < 0.001 | 0.002 |
| *NPIPB9* | 3.073 | < 0.001 | 0.002 |
| *PHF23* | 2.419 | < 0.001 | 0.009 |
| *HEXIM1* | 2.342 | < 0.001 | 0.009 |
| *KANSL1-AS1* | 2.967 | < 0.001 | 0.002 |
| *LSR* | 2.955 | < 0.001 | 0.002 |
| *FTL* | 2.684 | < 0.001 | 0.003 |
| *BCL2L12* | 2.367 | < 0.001 | 0.003 |
| *SNORD12C* | 3.257 | < 0.001 | 0.003 |

**Supplementary Table 5.** Top 50 genes that are differentially expressed in pDCs of At-Risk individuals in comparison with pDCs from healthy controls (HC).

| Gene | At-Risk pDCs vs. IFN$^{low}$ SLE pDCs Fold Change (log2) | P value | FDR |
|---|---|---|---|
| *COL24A1* | 4.046 | < 0.001 | 0.002 |
| *CDK11A* | 4.130 | < 0.001 | 0.001 |
| *FAM72C* | 4.744 | < 0.001 | 0.003 |
| *LMNA* | 5.449 | < 0.001 | < 0.001 |
| *TNFRSF4* | 5.748 | < 0.001 | < 0.001 |
| *CDK11B* | 4.207 | < 0.001 | 0.001 |
| *RNU6-1* | 5.202 | < 0.001 | < 0.001 |
| *RNU6-9* | 5.196 | < 0.001 | < 0.001 |
| *RNU6-7* | 5.202 | < 0.001 | < 0.001 |
| *RNU6-8* | 5.202 | < 0.001 | < 0.001 |
| *RNU6-2* | 5.196 | < 0.001 | < 0.001 |
| *ID3* | 6.455 | < 0.001 | < 0.001 |
| *IFI6* | 4.682 | < 0.001 | 0.003 |
| *SNORA16A* | 5.022 | < 0.001 | 0.005 |
| *POU3F1* | 7.210 | < 0.001 | < 0.001 |
| *SNORD46* | 4.793 | < 0.001 | < 0.001 |
| *HIST2H3D* | 5.510 | < 0.001 | < 0.001 |
| *HIST2H3A* | 4.801 | < 0.001 | < 0.001 |
| *HIST2H3C* | 4.801 | < 0.001 | < 0.001 |
| *TNFAIP8L2* | 3.967 | < 0.001 | 0.001 |
| *LMNA* | 5.894 | < 0.001 | < 0.001 |
| *IGSF9* | 6.193 | < 0.001 | < 0.001 |
| *GAS5-AS1* | 4.061 | < 0.001 | 0.003 |
| *NPL* | 4.650 | < 0.001 | 0.003 |
| *FAM72A* | 4.297 | < 0.001 | 0.008 |
| *ID2* | 5.343 | < 0.001 | < 0.001 |
| *FBXO48* | 4.007 | < 0.001 | 0.003 |
| *ANKRD36BP2* | 4.160 | < 0.001 | 0.002 |
| *SCARNA5* | 5.077 | < 0.001 | 0.003 |
| *RSRC1* | 3.969 | < 0.001 | 0.002 |
| *CACNA1D* | 5.556 | < 0.001 | 0.002 |
| *TNFSF10* | -4.893 | < 0.001 | 0.001 |
| *HES1* | 5.766 | < 0.001 | < 0.001 |
| *TNK2-AS1* | 3.486 | < 0.001 | 0.002 |
| *PCDH10* | 7.397 | < 0.001 | < 0.001 |
| *ARHGAP10* | 5.957 | < 0.001 | < 0.001 |
| *SCARNA22* | 4.668 | < 0.001 | 0.007 |
| *MSX1* | 5.869 | < 0.001 | 0.001 |
| *CXCL3* | 6.537 | < 0.001 | < 0.001 |
| *PCDH10* | 7.397 | < 0.001 | < 0.001 |
| *ELL2* | 5.151 | < 0.001 | < 0.001 |
| *CCDC127* | 4.327 | < 0.001 | 0.001 |
| *BRD9* | 3.428 | < 0.001 | 0.006 |
| *PTGER4* | 4.681 | < 0.001 | < 0.001 |
| *CDC20B* | 7.824 | < 0.001 | < 0.001 |
| *ENC1* | 5.279 | < 0.001 | < 0.001 |
| *LUCAT1* | 5.804 | < 0.001 | < 0.001 |
| *EGR1* | 3.934 | < 0.001 | 0.001 |
| *SNORD95* | 5.194 | < 0.001 | < 0.001 |
| *NQO2* | 4.500 | < 0.001 | 0.001 |

**Supplementary Table 6.** Top 50 genes that are differentially expressed in pDCs of At-Risk individuals in comparison with pDCs from IFN$^{low}$ SLE patients.

| | At-Risk pDCs vs. IFN$^{high}$ SLE pDCs | | |
|---|---|---|---|
| **Gene** | **Fold Change (log2)** | ***P* value** | **FDR** |
| *NUP210L* | 4.718 | < 0.001 | 0.009 |
| *LMNA* | 5.127 | < 0.001 | 0.002 |
| *PCNX2* | 5.859 | < 0.001 | 0.001 |
| *PLEKHN1* | 4.941 | < 0.001 | 0.009 |
| *HES4* | 5.030 | < 0.001 | 0.002 |
| *TNFRSF4* | 4.667 | < 0.001 | 0.004 |
| *TNFRSF25* | 4.450 | < 0.001 | 0.007 |
| *TNFRSF9* | 6.082 | < 0.001 | 0.001 |
| *ID3* | 4.972 | < 0.001 | 0.001 |
| *NCMAP* | 4.261 | < 0.001 | 0.005 |
| *IFI6* | 6.506 | < 0.001 | 0.000 |
| *SNORA16A* | 5.387 | < 0.001 | 0.003 |
| *GBP2* | 5.083 | < 0.001 | 0.002 |
| *FAM46C* | 4.362 | < 0.001 | 0.003 |
| *LMNA* | 5.203 | < 0.001 | 0.001 |
| *FCGR2C* | 4.781 | < 0.001 | 0.007 |
| *SNORD74* | 4.855 | < 0.001 | 0.004 |
| *GLRX2* | 4.303 | < 0.001 | 0.008 |
| *LAX1* | 4.786 | < 0.001 | 0.009 |
| *DUSP10* | 4.680 | < 0.001 | 0.010 |
| *RNA5S4* | 4.762 | < 0.001 | 0.005 |
| *RNA5S2* | 4.762 | < 0.001 | 0.005 |
| *RNA5S3* | 4.762 | < 0.001 | 0.005 |
| *RNA5S1* | 4.762 | < 0.001 | 0.005 |
| *RNA5S15* | 4.762 | < 0.001 | 0.005 |
| *RNA5S16* | 4.762 | < 0.001 | 0.005 |
| *RNA5S14* | 4.762 | < 0.001 | 0.005 |
| *RNA5S5* | 4.762 | < 0.001 | 0.005 |
| *RNA5S12* | 4.762 | < 0.001 | 0.005 |
| *RNA5S6* | 4.762 | < 0.001 | 0.005 |
| *RNA5S8* | 4.762 | < 0.001 | 0.005 |
| *RNA5S13* | 4.762 | < 0.001 | 0.005 |
| *RNA5S7* | 4.762 | < 0.001 | 0.005 |
| *RNA5S10* | 4.762 | < 0.001 | 0.005 |
| *RNA5S9* | 4.736 | < 0.001 | 0.005 |
| *RNA5S11* | 4.762 | < 0.001 | 0.005 |
| *RNA5S17* | 4.762 | < 0.001 | 0.005 |
| *EXO1* | 6.374 | < 0.001 | < 0.001 |
| *RASGRP3* | 4.696 | < 0.001 | 0.005 |
| *EML6* | 5.493 | < 0.001 | 0.003 |
| *ITGA6* | 4.334 | < 0.001 | 0.005 |
| *TMEM198* | 5.427 | < 0.001 | 0.004 |
| *SERPINE2* | -4.011 | < 0.001 | 0.009 |
| *SP140* | 4.826 | < 0.001 | 0.002 |
| *RAMP1* | 5.191 | < 0.001 | 0.006 |
| *CENPA* | 5.367 | < 0.001 | 0.003 |
| *CDC42EP3* | 5.704 | < 0.001 | < 0.001 |
| *ANKRD36BP2* | 5.174 | < 0.001 | 0.001 |
| *IL1B* | 4.827 | < 0.001 | 0.007 |
| *TUBA4A* | 4.224 | < 0.001 | 0.002 |

**Supplementary Table 7.** Top 50 genes that are differentially expressed in pDCs of At-Risk individuals in comparison with pDCs from IFN$^{high}$ SLE patients.

| REAGENT or RESOURCE | SOURCE | IDENTIFIER |
|---|---|---|
| **Antibodies** | | |
| Anti-human CD3 clone BW264/56, VioBlue | Miltenyi Biotec | Cat# 130-094-363 |
| Anti-human CD3 clone BW264/56, VioGreen | Miltenyi Biotec | Cat# 130-096-910 |
| Anti-human CD4 clone M-T466, APC-Vio770 | Miltenyi Biotec | Cat# 130-100-457 |
| Anti-human CD19 clone LT19, VioBlue | Miltenyi Biotec | Cat# 130-098-598 |
| Anti-human CD14 clone TÜK4, VioBlue | Miltenyi Biotec | Cat# 130-094-364 |
| Anti-human CD56 clone B159, BV450 | BD Biosciences | Cat# 560360 |
| Anti-human CD11c clone MJ4-27G12, VioBlue | Miltenyi Biotec | Cat# 130-097-328 |
| Anti-human HLA-DR clone AC122, APC-Vio770 | Miltenyi Biotec | Cat# 130-104-200 |
| Anti-human CD123 clone AC145, PerCP-Vio700 | Miltenyi Biotec | Cat# 130-103-802 |
| Anti-human CD303 (BDCA-2) clone AC144, FITC | Miltenyi Biotec | Cat# 130-090-510 |
| Anti-human CD304 (BDCA-4) clone AD5-17F6, VioBright FITC | Miltenyi Biotec | Cat# 130-104-272 |
| Anti-human CD85g clone REA100, PE-Vio770 | Miltenyi Biotec | Cat# 130-099-009 |
| Anti-human CD85j clone GHI/75, PE-Vio770 | Miltenyi Biotec | Cat# 130-101-552 |
| Anti-human CD69 clone FN50, FITC | Miltenyi Biotec | Cat# 130-092-166 |
| Anti-human CD25 clone 4E3, PE | Miltenyi Biotec | Cat# 130-091-024 |
| Anti-human CD317 clone RS38E, PE | BioLegend | Cat# 348406 |
| Anti-human IFN-α clone LT27:295, APC | Miltenyi Biotec | Cat# 130-092-602 |
| Anti-human TNF-α cA2, PE-Vio770 | Miltenyi Biotec | Cat# 130-096-755 |
| Anti-human IL-6 clone MQ2-13A5, PE | Miltenyi Biotec | Cat# 130-096-086 |
| Anti-human IFN-γ clone 45-15, APC | Miltenyi Biotec | Cat# 130-091-640 |
| Anti-human IL-10 clone JES3-9D7, PE | Miltenyi Biotec | Cat# 130-096-043 |
| Anti-human TNF-α clone Mab11, APC/Cy7 | BioLegend | Cat# 502944 |
| Anti-human IFN-γ clone 4S.B3, PE/Cy7 | BioLegend | Cat# 502528 |
| Anti-human IL-17A clone BL168, APC | BioLegend | Cat# 512334 |
| Anti-human TLR9 clone eB72-1665, APC | BD Biosciences | Cat# 560428 |
| Anti-human TLR7 clone 533707, PE | R&D | Cat# IC5875P |
| Anti-human FoxP3 clone 3G3, APC | Miltenyi Biotec | Cat# 130-093-013 |
| Human TNF alpha antibody clone 1825 | R&D Systems | Cat# MAB210-SP |
| Anti-Mx1 rabbit polyclonal | Abcam | Cat# ab95926 |
| Anti-BDCA-4 rabbit monoclonal | Abcam | Cat# ab81321 |

| | | |
|---|---|---|
| Anti-rabbit polyclonal biotin-conjugated secondary antibody | Sigma-Aldrich | Cat# SAB3700930 |
| AffiniPure Donkey Anti-Rabbit IgG (H+L), PE | Jackson ImmunoResearch | Cat# 711-005-152 |
| **Chemicals, Peptides, and Recombinant Proteins** | | |
| ODN 2216 | Miltenyi Biotec | Cat# 130-100-243 |
| ORN R-2336 | Miltenyi Biotec | Cat# 130-104-431 |
| Human IL-3, premium grade | Miltenyi Biotec | Cat# 130-095-071 |
| Human TNF-α, premium grade | Miltenyi Biotec | Cat# 130-094-014 |
| Hydrogen peroxide solution [30% (w/w) in $H_2O_2$] | Sigma-Aldrich | Cat# H1009 |
| Prolong Gold Antifade Mountant | ThermoFischer Scientific | Cat# P36930 |
| Poly(I:C) LMW | InvivoGen | Cat# 31852-29-6 |
| Poly(dA:dT) | InvivoGen | Cat# 86828-69-5 |
| TaqMan Universal PCR master Mix | ThermoFischer Scientific | Cat# 4304437 |
| SYBR Green PCR Master Mix | ThermoFischer Scientific | Cat# 4309155 |
| T cell activation/expansion kit | Miltenyi Biotec | Cat# 130-091-441 |
| 7-AAD Staining Solution | Miltenyi Biotec | Cat# 130-111-568 |
| Annexin V-FTIC kit | Miltenyi Biotec | Cat# 130-092-052 |
| DQ Ovalbumin | Molecular Probes | Cat# D-12053 |
| Streptavidin peroxidase | Sigma-Aldrich | Cat# S2438 |
| **Critical Commercial Assays** | | |
| Diamond Plasmacytoid Dendritic Cell Isolation Kit II | Miltenyi Biotec | Cat# 130-097-240 |
| Naive $CD4^+$ T Cell Isolation Kit II | Miltenyi Biotec | Cat# 130-094-131 |
| Intracellular Fixation & Permeabilization Buffer Set | eBioscience | Cat# 88-8824-00 |
| FoxP3 Staining Buffer Set | Miltenyi Biotec | Cat# 130-093-142 |
| CellTrace Violet Cell Proliferation Kit | ThermoFischer Scientific | Cat# C34571 |
| Total RNA Purification Kit | Norgen Biotek | Cat# 17200 |
| PicoPure RNA Isolation Kit | ThermoFischer Scientific | Cat# KIT0204 |

| | | |
|---|---|---|
| Qubit RNA HS Assay Kit | ThermoFisher Scientific | Cat# Q32852 |
| SMART-Seq V4 ultra low Input RNA Kit | Takara Bio | Cat# 634888 |
| Nextera XT DNA Library Preparation Kit | Illumina | Cat# FC-131-1024 |
| Telomere PNA Kit/FITC for Flow Cytometry | Agilent | Cat# K532711-8 |
| Superfrost plus slides | ThermoFisher Scientific | Cat# J1800AMNT |
| RNAscope Multiplex Fluorescent Reagent Kit v2 | Advanced Cell Diagnostics | Cat# 323100 |
| TSA Cy 3, Cy 5, TMR, Fluorescein Evaluation Kit | Perkin Elmer | Cat# NEL760001KT |
| RevertAid First Strand cDNA Synthesis Kit | ThermoFisher Scientific | Cat# K1622 |
| QuantiFast SYBR Green PCR Kit | Qiagen | Cat# 204054 |
| Quick-RNA MiniPrep Kit | Zymo Research | Cat# R1055A |
| Human IFN alpha Platinum ELISA | Invitrogen | Cat# BMS216 |
| **Experimental Models: Cell Lines** | | |
| Human: 1301 cell line | Sigma-Aldrich | Cat# 0105161 |
| **Oligonucleotides** | | |
| U6snRNA Primer: Forward 5’-CTCGCTTCGGCAGCACA-3’Reverse 5’-AACGCTTCACGAATTTGC-3’ | Sigma-Aldrich | N/A |
| IFNK Primer | Qiagen | Cat# QT00197512 |
| IFNA Primer | Qiagen | Cat# QT00212527 |
| IFNB1 Primer | Qiagen | Cat# QT00203763 |
| IFNL1 Primer | Qiagen | Cat# QT00222495 |

| | | |
|---|---|---|
| **Software and Algorithms** | | |
| Prism 7 | Graphpad Software, Inc. | https://www.graphpad.com/ |
| FACS DiVA | BD Biosciences | http://www.bdbiosciences.com/ |
| CytExpert 2.0 | Beckman Coulter | https://www.beckman.com/ |
| Fluidigm Real Time PCR Analysis | Fluidigm | https://www.fluidigm.com/software |
| FastQC | BaseSpace Labs | https://www.illumina.com/ |

| | | |
|---|---|---|
| Cutadapt | Cutadapt | http://cutadapt.readthedocs.io/en/stable/index.html/ |
| Table Browser | UCSC Genome Browser | https://genome.ucsc.edu/ |
| QualiMap 2.0 | QualiMap | http://qualimap.bioinfo.cipf.es/ |
| Picard tools | Broad Institute | https://broadinstitute.github.io/picard/ |
| ReactomePA | Bioconductor | https://www.bioconductor.org/ |
| KEGG | Kyoto University | https://www.genome.jp/kegg/ |
| Nikon NIS Elements | Nikon Instruments | https://www.nikoninstruments.com/ |
| **Other** | | |
| 50mL Leucosep Tubes | Greiner Bio-One | Cat# 89048-938 |

**Supplementary Table 8.** Key resources.
