# Supplementary material for: Functionally impaired plasmacytoid dendritic cells and non-haematopoietic sources of type I interferon characterize human autoimmunity
Source: Nat Commun. 2020 Dec 1;11:6149. doi: 10.1038/s41467-020-19918-z (PMC7708979; doi:10.1038/s41467-020-19918-z)
Supplement: Supplementary file 2 — Reporting Summary [file 41467_2020_19918_MOESM2_ESM.pdf]

nature research

Corresponding author(s): Edward Vital
Last updated by author(s): Oct 1, 2020

Reporting Summary

Nature Research wishes to improve the reproducibility of the work that we publish. This form provides structure for consistency and transparency in reporting. For further information on Nature Research policies, see our Editorial Policies and the Editorial Policy Checklist.

Statistics

For all statistical analyses, confirm that the following items are present in the figure legend, table legend, main text, or Methods section.

n/a	Confirmed	
☐	☒	The exact sample size (n) for each experimental group/condition, given as a discrete number and unit of measurement
☐	☒	A statement on whether measurements were taken from distinct samples or whether the same sample was measured repeatedly
☐	☒	The statistical test(s) used AND whether they are one- or two-sided<br>*Only common tests should be described solely by name; describe more complex techniques in the Methods section.*
☒	☐	A description of all covariates tested
☒	☐	A description of any assumptions or corrections, such as tests of normality and adjustment for multiple comparisons
☐	☒	A full description of the statistical parameters including central tendency (e.g. means) or other basic estimates (e.g. regression coefficient) AND variation (e.g. standard deviation) or associated estimates of uncertainty (e.g. confidence intervals)
☒	☐	For null hypothesis testing, the test statistic (e.g. F, t, r) with confidence intervals, effect sizes, degrees of freedom and P value noted<br>*Give P values as exact values whenever suitable.*
☒	☐	For Bayesian analysis, information on the choice of priors and Markov chain Monte Carlo settings
☒	☐	For hierarchical and complex designs, identification of the appropriate level for tests and full reporting of outcomes
☒	☐	Estimates of effect sizes (e.g. Cohen's d, Pearson's r), indicating how they were calculated

Our web collection on statistics for biologists contains articles on many of the points above.

Software and code

Policy information about availability of computer code

Data collection	Microsoft Excel<br>Prism 7 Graphpad Software, Inc. https://www.graphpad.com/
Data analysis	Prism 7 Graphpad Software, Inc. https://www.graphpad.com/<br>FACS DiVA BD Biosciences http://www.bdbiosciences.com/<br>CytExpert 2.0 Beckman Coulter https://www.beckman.com/<br>Fluidigm Real Time PCR Analysis Fluidigm https://www.fluidigm.com/software<br>FastQC BaseSpace Labs https://www.illumina.com/<br>Cutadapt Cutadapt http://cutadapt.readthedocs.io/en/stable/index.html/<br>Table Browser UCSC Genome Browser https://genome.ucsc.edu/<br>QualiMap 2.0 QualiMap http://qualimap.bioinfo.cipf.es/<br>Picard tools Broad Institute https://broadinstitute.github.io/picard/<br>ReactomePA Bioconductor https://www.bioconductor.org/<br>KEGG Kyoto University https://www.genome.jp/kegg/<br>Nikon NIS Elements Nikon Instruments https://www.nikoninstruments.com/

For manuscripts utilizing custom algorithms or software that are central to the research but not yet described in published literature, software must be made available to editors and reviewers. We strongly encourage code deposition in a community repository (e.g. GitHub). See the Nature Research guidelines for submitting code & software for further information.

Data

Policy information about availability of data

All manuscripts must include a data availability statement. This statement should provide the following information, where applicable:

- Accession codes, unique identifiers, or web links for publicly available datasets
- A list of figures that have associated raw data
- A description of any restrictions on data availability

Please see the Source Data file for raw data for each figure. RNA-sequencing data are available in BioProject, https://www.ncbi.nlm.nih.gov/bioproject, accession number PRJNA645252. A key resources table is provided in Supplemental Table 8. Any other data are available on request from the authors.

Field-specific reporting

Please select the one below that is the best fit for your research. If you are not sure, read the appropriate sections before making your selection.

☒ Life sciences ☐ Behavioural & social sciences ☐ Ecological, evolutionary & environmental sciences

For a reference copy of the document with all sections, see nature.com/documents/nr-reporting-summary-flat.pdf

Life sciences study design

All studies must disclose on these points even when the disclosure is negative.

Sample size	No sample size calculation was undertaken
Data exclusions	No data were excluded from the analysis
Replication	We presented multiple replicates for all experiments. We provided confirmation by additional techniques for key findings
Randomization	Samples were collected randomly from individuals in known, specified diagnostic groups. There was no randomisation of individuals to treatment as this was not appropriate for the experimental design. Detailed analysis of various clinical subgroups has been shown in Figure 1.
Blinding	Lab analysis was undertaken blind to diagnostic group

Reporting for specific materials, systems and methods

We require information from authors about some types of materials, experimental systems and methods used in many studies. Here, indicate whether each material, system or method listed is relevant to your study. If you are not sure if a list item applies to your research, read the appropriate section before selecting a response.

Materials & experimental systems

n/a	Involved in the study	
☐	☒	Antibodies
☒	☐	Eukaryotic cell lines
☒	☐	Palaeontology and archaeology
☒	☐	Animals and other organisms
☐	☒	Human research participants
☐	☒	Clinical data
☒	☐	Dual use research of concern

Methods

n/a	Involved in the study	
☒	☐	ChIP-seq
☐	☒	Flow cytometry
☒	☐	MRI-based neuroimaging

Antibodies

Antibodies used

Anti-human CD3 clone BW264/56, VioBlue Miltenyi Biotec Cat# 130-094-363
Anti-human CD3 clone BW264/56, VioGreen Miltenyi Biotec Cat# 130-096-910
Anti-human CD4 clone M-T466, APC-Vio770 Miltenyi Biotec Cat# 130-100-457
Anti-human CD19 clone LT19, VioBlue Miltenyi Biotec Cat# 130-098-598
Anti-human CD14 clone TÜK4, VioBlue Miltenyi Biotec Cat# 130-094-364
Anti-human CD56 clone B159, BV450 BD Biosciences Cat# 560360
Anti-human CD11c clone MJ4-27G12, VioBlue Miltenyi Biotec Cat# 130-097-328
Anti-human HLA-DR clone AC122, APC-Vio770 Miltenyi Biotec Cat# 130-104-200
Anti-human CD123 clone AC145, PerCP-Vio700 Miltenyi Biotec Cat# 130-103-802
Anti-human CD303 (BDCA-2) clone AC144, FITC Miltenyi Biotec Cat# 130-090-510
Anti-human CD304 (BDCA-4) clone AD5-17F6, VioBright FITC Miltenyi Biotec Cat# 130-104-272

Anti-human CD85g clone REA100, PE-Vio770 Miltenyi Biotec Cat# 130-099-009
Anti-human CD85j clone GHI/75, PE-Vio770 Miltenyi Biotec Cat# 130-101-552
Anti-human CD69 clone FN50, FITC Miltenyi Biotec Cat# 130-092-166
Anti-human CD25 clone 4E3, PE Miltenyi Biotec Cat# 130-091-024
Anti-human CD317 clone RS38E, PE BioLegend Cat# 348406
Anti-human IFN-α clone LT27:295, APC Miltenyi Biotec Cat# 130-092-602
Anti-human TNF-α cA2, PE-Vio770 Miltenyi Biotec Cat# 130-096-755
Anti-human IL-6 clone MQ2-13A5, PE Miltenyi Biotec Cat# 130-096-086
Anti-human IFN-γ clone 45-15, APC Miltenyi Biotec Cat# 130-091-640
Anti-human IL-10 clone JES3-9D7, PE Miltenyi Biotec Cat# 130-096-043
Anti-human TNF-α clone Mab11, APC/Cy7 BioLegend Cat# 502944
Anti-human IFN-γ clone 4S.B3, PE/Cy7 BioLegend Cat# 502528
Anti-human IL-17A clone BL168, APC BioLegend Cat# 512334
Anti-human TLR9 clone eB72-1665, APC BD Biosciences Cat# 560428
Anti-human TLR7 clone 533707, PE R&D Cat# IC5875P
Anti-human FoxP3 clone 3G3, APC Miltenyi Biotec Cat# 130-093-013
Human TNF alpha antibody clone 1825 R&D Systems Cat# MAB210-SP

Validation: See supplementary information

Human research participants

Policy information about studies involving human research participants

Population characteristics: Peripheral blood and skin biopsies were obtained from healthy individuals and patients from different disease groups (SLE, pSS, At-Risk). Patients were recruited based on 2012 SLICC classification criteria for SLE, 2016 ACR/EULAR classification criteria for pSS, while At-Risk individuals were classified as ANA positive,1 SLE clinical criterion, symptom duration <12 months and treatment-naïve. Supplemental Table 1 summarizes the characteristics and treatment of SLE patients.

Recruitment: All participants attending the clinic during the study were invited to participate. Recruitment flow chart for the At Risk individuals has previously been published (PMID: 29929956)

Ethics oversight: Leeds East – National Research Ethics Committee (REC 10/H1306/88)

Note that full information on the approval of the study protocol must also be provided in the manuscript.

Clinical data

Policy information about clinical studies

All manuscripts should comply with the ICMJEguidelines for publication of clinical research and a completedCONSORT checklist must be included with all submissions.

Clinical trial registration: This was not a clinical trial.

Study protocol: The study protocol is available from the corresponding author.

Data collection: Participants attending Leeds Teaching Hospitals Connective Tissue Disease clinic with the relevant diagnoses between 2015 - 2018 were invited to participate. Recruitment flow chart for the At Risk individuals has previously been published (PMID: 29929956)

Outcomes: This was not a clinical trial and did not have primary and secondary clinical outcome measures.

Flow Cytometry

Plots

Confirm that:

- [x] The axis labels state the marker and fluorochrome used (e.g. CD4-FITC).
- [x] The axis scales are clearly visible. Include numbers along axes only for bottom left plot of group (a 'group' is an analysis of identical markers).
- [x] All plots are contour plots with outliers or pseudocolor plots.
- [x] A numerical value for number of cells or percentage (with statistics) is provided.

Methodology

Sample preparation: Human PBMCs were separated from whole blood by a density gradient centrifugation method using Leucosep tubes (Greiner Bio-One). pDCs were purified from freshly isolated PBMCs by negative selection using the Diamond Plasmacytoid Dendritic Cell Isolation Kit II (Miltenyi Biotec). Naïve CD4+ T cells were purified by negative selection using the Naive CD4+ T Cell Isolation Kit II (Miltenyi Biotec). Pre-enriched pDCs were sorted using an antibody to BDCA-4 (Miltenyi Biotec). Cell sorting was carried out at the SCIF Flow Cytometry and Imaging Facility of the Wellcome Trust Brenner Building, University of Leeds, with a BD Influx 6 Way Cell Sorter (BD Biosciences). Cells were cultured in RPMI medium 1640 with GlutaMAX supplement

(ThermoFisher Scientific) containing 10% (vol/vol) FBS and 100 U/ml penicillin/streptomycin. For cytokine production, PBMCs were stimulated with 2 µM class A CpG (ODN 2216; Miltenyi Biotec) or 2 µM ORN R-2336 (Miltenyi Biotec). For pDC/T-cell co-culture, purified pDCs (1 x 105) were cultured with autologous or allogeneic naïve CD4+ T cells (5 x 105) for 5 days in the absence or presence of anti-CD3/CD28 beads (T cell activation/expansion kit; Miltenyi Biotec) at a bead-to-cell ratio of 1:2. Cytokine production was measured by intracellular staining.

Instrument

LSRII (BD Biosciences) or Cytoflex S (Beckman Coulter)

Software

FACS DiVA (BD Biosciences) or CytExpert (Beckman Coulter)

Cell population abundance

To investigate disease-associated transcriptional changes in pDCs, we purified pDCs from healthy controls (n = 8), At-Risk individuals (n = 4) and SLE patients (n = 13) by negative selection then sorted the cells to achieve purity >99% based on CD304 (BDCA-4) expression.

Gating strategy

pDCs are characterized by the lack of lineage markers CD3 (T cells), CD19 (B cells), CD56 (NK cells), CD14 (monocytes) and CD11c (conventional DCs), intermediate to high expression of HLA-DR (MHC-II), high expression of CD123 (IL-3R) and other markers such as CD303 (BDCA-2) and CD304 (BDCA-4). pDCs were gated as lineage-HLA-DR+CD123+CD303+ cells

☒ Tick this box to confirm that a figure exemplifying the gating strategy is provided in the Supplementary Information.
